# Coupling traction force patterns and actomyosin wave dynamics reveals mechanics of cell motion

**DOI:** 10.15252/msb.202110505

**Published:** 2021-12-13

**Authors:** Elisabeth Ghabache, Yuansheng Cao, Yuchuan Miao, Alex Groisman, Peter N Devreotes, Wouter‐Jan Rappel

**Affiliations:** ^1^ Department of Physics University of California, San Diego La Jolla CA USA; ^2^ Department of Cell Biology School of Medicine Johns Hopkins University Baltimore MD USA

**Keywords:** chemotaxis, computational modeling, migration modes, signaling components, traction force microscopy, Cell Adhesion, Polarity & Cytoskeleton

## Abstract

Motile cells can use and switch between different modes of migration. Here, we use traction force microscopy and fluorescent labeling of actin and myosin to quantify and correlate traction force patterns and cytoskeletal distributions in *Dictyostelium discoideum* cells that move and switch between keratocyte‐like fan‐shaped, oscillatory, and amoeboid modes. We find that the wave dynamics of the cytoskeletal components critically determine the traction force pattern, cell morphology, and migration mode. Furthermore, we find that fan‐shaped cells can exhibit two different propulsion mechanisms, each with a distinct traction force pattern. Finally, the traction force patterns can be recapitulated using a computational model, which uses the experimentally determined spatiotemporal distributions of actin and myosin forces and a viscous cytoskeletal network. Our results suggest that cell motion can be generated by friction between the flow of this network and the substrate.

## Graphical abstract

A combination of imaging and computational modeling is used to investigate the traction force patterns and the distribution of actin and myosin in three different modes of migration in *Dictyostelium discoideum* cells.

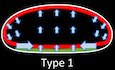

## Introduction

Eukaryotic cells can move using different modes of migration. For example, amoeboid cells move through the extension of randomly placed actin‐filled pseudopods, fish keratocytes move with a near‐constant morphology in a persistent fashion, neuronal cells use filopodia for migration, and some cells display oscillatory motion during which the basal surface undergoes periodic variations (Webb & Horwitz, 2003; Chan & Odde, 2008; Charras & Paluch, 2008; Keren *et al*, 2008; Bosgraaf & Van Haastert, 2009; Chan *et al*, 2013). These different modes and morphologies are often used to characterize cell types. However, cells of the same type can exhibit multiple modes and can easily switch between them. The ability of cells to change their migration mode, depending on external or internal cues, has been implicated in diseases, including cancer metastasis (Yilmaz & Christofori, 2010; Friedl & Alexander, 2011; Kim *et al*, 2021).

The different modes of migration are correlated with waves of signal transduction and cytoskeletal components propagating along the cell cortex and responsible for contraction and protrusion (Weiner *et al*, 2007; Case & Waterman, 2011; Allard & Mogilner, 2013; Inagaki & Katsuno, 2017). The waves originate from the excitable dynamics of the signaling network and can be triggered spontaneously or by a sufficiently large stimulus. The resulting wave can then continue to propagate outward and away from the initiation site or can fail to propagate further, resulting in a spatially restricted excitation and protrusion (Miao *et al*, 2017). In addition, the excitable system can produce oscillatory initiation of symmetric waves, leading to periodic flattening. Furthermore, oscillatory signaling dynamics can result in polarized waves that push the membrane on the one side of the cell forward with a constant speed (Cao *et al*, 2019a, 2019b). The dramatically different migration modes displayed by the same cell type can be traced to slight shifts in the strength of feedback loops within the underlying signaling system, which controls the cell protrusions and contractions.

The distinct migration modes have in common that the various protrusions and contractions can only generate motion through the exertion of forces onto the extracellular environment. These forces can be measured using traction force microscopy (TFM), which enables real‐time spatially resolved measurements of forces exerted onto the substrate (Plotnikov *et al*, 2014; Style *et al*, 2014; Roca‐Cusachs *et al*, 2017). Earlier studies revealed that the traction force maps differ significantly for different cells. Gliding fish keratocyte cells, for example, exert large traction forces at two foci at the posterior end, and these foci are persistent and nearly symmetric with respect to the longitudinal axis of the keratocyte (Fournier *et al*, 2010; Barnhart *et al*, 2015; Sonoda *et al*, 2016). In contrast, chemotactic *Dictyostelium* cells and neutrophils migrating in the amoeboid mode were shown to have two traction force poles, near the front and near the back (Del Alamo *et al*, 2007, 2013; Lombardi *et al*, 2007; Delanoe‐Ayari *et al*, 2010; Alvarez‐Gonzalez *et al*, 2014). A general understanding of the role of the different force patterns in cell migration is, however, still lacking.

## Results

**Figure 1:**
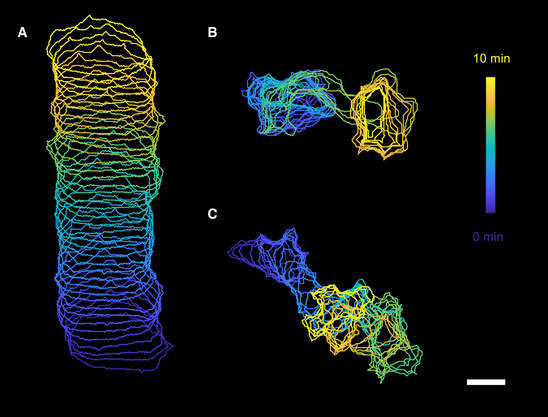
Three different migration modes A–CCell outlines at different times for the three distinct cell migration modes: fan‐shaped cells (A), oscillatory cells (B), and amoeboid cells (C; scale bar: 10 μm).

Here, we determined how cell migration, signaling, and traction forces are coupled in different modes of migration by quantifying the traction force maps using thin, soft silicone gel substrates with tracer particles attached to the gel surfaces (Gutierrez *et al*, 2011; Han *et al*, 2015). We use cells of the social amoeba *Dictyostelium discoideum*, which display a variety of migration modes when starved under low cell density conditions or when synthetically altered to have decreased phosphatidylinositol‐4,5‐bisphosphate levels or increased Ras/Rap‐related activities (Asano *et al*, 2004; Miao *et al*, 2017; Cao *et al*, 2019a). These modes consist of a keratocyte‐like mode, an oscillatory mode, and an amoeboid mode (Fig 1A–C). Each of these modes has its own wave dynamics, which determines their morphology and migration properties (Miao *et al*, 2017; Cao *et al*, 2019b). The fan‐shaped cells contain a broad and stable traveling wave of cytoskeletal components, including actin, which moves at a constant speed in a persistent direction. Oscillatory cells display an actin wave that originates at the basal surface of the cell and reaches the entire cell perimeter simultaneously. Finally, the pseudopods of amoeboid cells result from waves that expand narrowly and originate at random locations.

### Stable traveling waves result in fan‐shaped cells

**Figure 2:**
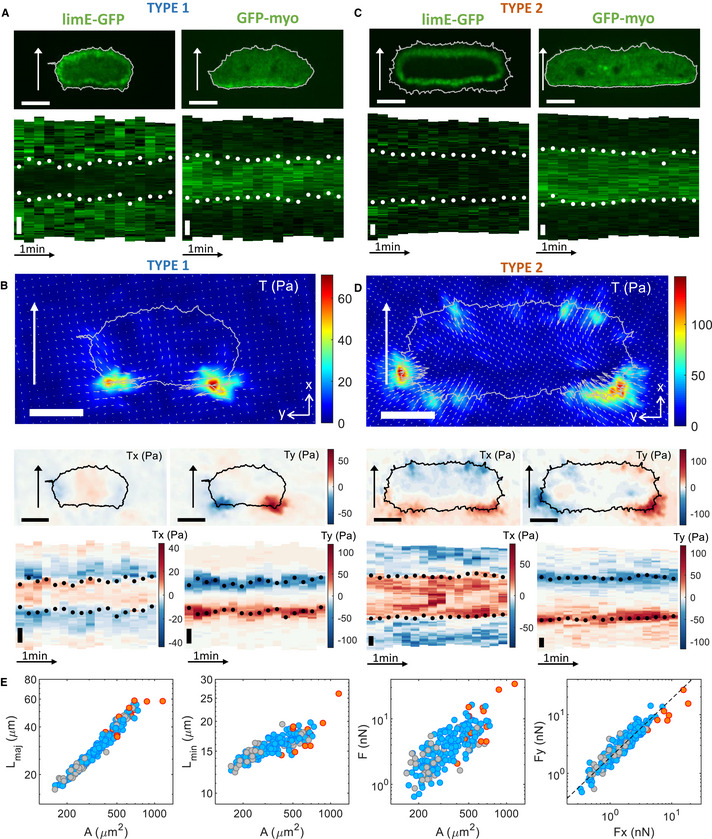
Traction force maps and distributions of signaling components in fan‐shaped cells Snapshots of LimE‐GFP, GFP‐myo, and corresponding kymographs for type 1 cells. Corresponding speeds are 10.8 µm/min and 9.4 µm/min. White dots in the fluorescent kymographs indicate the location of the two poles of force at each time point, as extracted from the corresponding stress kymographs.Stress maps quantifying the magnitude of the force per area using a color scale with blue/red corresponding to small/large stresses and the direction of forces using vectors. Fan‐shaped cells were rotated so that the vertical (*x*) axis is the direction of motion (see Materials and Methods). Shown are the overall stress *T*, the stress in the direction of motion *T*_x_, and the stress perpendicular to motion *T_y_* for type 1 cell with the LimE marker, and the corresponding *T*_x_ and *T_y_* kymographs along the cell's outlines.Snapshots of LimE‐GFP, GFP‐myo, and corresponding kymographs for type 2 cells. Corresponding speeds are 5.6 and 8.7 µm/min. White dots in the fluorescent kymographs indicate the location of the two poles of force at each time point.Stress maps of *T*, *T*_x_ and *T_y_* for type 2 cell with the LimE marker, and corresponding *T*_x_ and *T_y_* kymographs along the cell's outlines.Left three panels: Major and minor axes of the cells, *L*_maj_ and *L*_min_, and total force F as a function of the cell area A. Right panel: Force perpendicular to the motion, *F_y_*, as a function of the force parallel to the motion, *F_x_*. The dashed line represents a linear fit with a slope of 1.87 (*r* ^2^ = 0.88). The plots present averaged values for each cell based on the duration of each recording (cell type 1: blue markers, cell type 2: orange markers, and less stable cells: gray markers [see Materials and Methods]). The basal area and speed of type 2 cells was larger than for type 1 cells: 628 (502/692) μm^2^ and 6.0 (5.4/8.2) μm/min (*N* = 12 biological replicates) vs. 326 (258/461) μm^2^ and 10.8 (9.4/12.3) μm/min (*N* = 161 biological replicates; *P* = 1.9 × 10^−6^ and 2.6 × 10^−7^), while the median ratio between the pole–pole distance and the cell's length was 0.75 (0.70/0.79, *N* = 161 biological replicates) for type 1 cells and 0.84 (0.77/0.90, *N* = 12 biological replicates) for type 2 cells (*P* = 2.2 × 10^−3^). Data information: The arrows indicate the direction of motion, and black dots in the kymographs correspond to the location of maximum stress. All scale bars in the figure: 10 μm.

We first determined how the key cytoskeletal components actin and myosin were distributed near the substrate in fan‐shaped cells (Fig 2). As expected, the cytoskeletal distributions were stationary with the cell's frame of reference (Fig 2A, Movie EV1 and EV2). Surprisingly, however, we observed two qualitatively different patterns, which we will call type 1 and type 2. For type 1 cells, the distribution of freshly polymerized filamentous actin (F‐actin), measured using LimE‐GFP (Materials and Methods), formed a ring that is positioned at the membrane of the front of the cell and slightly ahead of the back of the cell (Fig 2A, upper left panel). This ring is propagating as a wave with constant shape and speed, resulting in a near‐constant cell morphology (Fig 1A). The distribution of the contractile protein myosin II, visualized using GFP‐myo, showed an elevated band parallel to the back of the cell (Fig 2A, upper right panel), consistent with earlier results (Asano *et al*, 2004). Double labeling with GFP‐myo and LimE‐RFP showed that this myosin band was positioned between the rear membrane and the actin ring and that the location where the LimE‐GFP ring detached from the membrane coincided with the two ends of the myosin region (Appendix Fig S1A). Furthermore, labeling cells with lifeAct‐GFP, a marker for all the F‐actin in the cell (Riedl *et al*, 2008), revealed that F‐actin is also present at the rear membrane of the cell (Appendix Fig S1B).

To further quantify the distributions of the cytoskeletal components, we computed kymographs, which represent the fluorescent intensity along the membrane as a function of time. Consistent with our experimental observations, the kymograph that represents the LimE‐GFP distribution along the membrane showed elevated fluorescence levels everywhere, except at the posterior edge of the cell (Fig 2A, lower left panel), while the kymographs of the fluorescent intensity of GFP‐myo (Fig 2A, lower right panel) showed a region of high fluorescence that corresponds to the back of the cell.

**Figure EV1:**
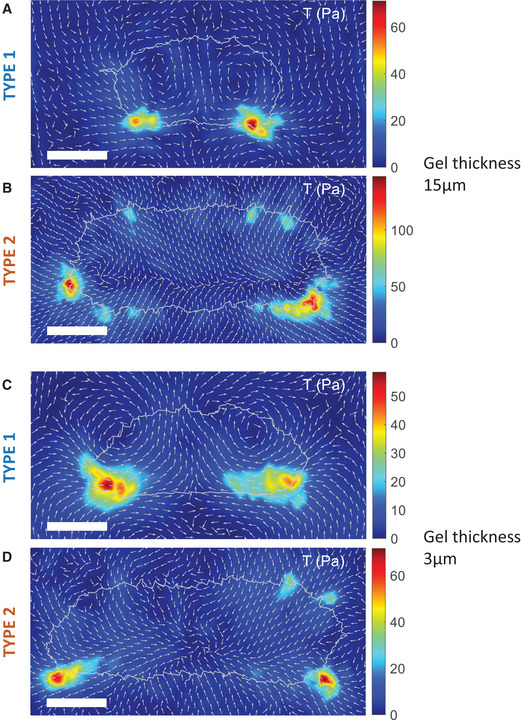
Detailed traction force maps for different gel thickness A, BDetailed TFMs of type 1 and type 2 fan‐shaped cells of Fig 2 using a gel with a thickness of 15 μm.C, DAs (A) and (B) but now for a gel with a thickness of 3 μm. Markers only indicate the direction of the stress, regardless of the magnitude, so that the direction is visible even for low stresses. Data information: The arrows indicate the traction force. All scale bars in the figure: 10 μm.

We next computed the traction force maps of these type 1 fan‐shaped cells from the bead displacement map (Appendix Fig S2A; see Materials and Methods). The resulting stress map revealed that the stress was largest at the posterior corners (Fig 2B, Movie EV3, and Fig EV1A). Interestingly, however, the forces in the front half of these cells were in the forward direction, indicating that the force exerted onto the substrate is directed forward. In other words, the cell–substrate forces in the front half of the cell are pointing in the direction of motion. Furthermore, as can also be seen from the more detailed map in Fig EV1A, the traction force map displayed two counter‐rotating vortices, located in the left and right part of the cell.

The kymograph of the stress in the direction of motion, *T*_x_, also clearly showed the forward‐oriented forces at the anterior edge of the cell: *T*_x_ was positive at the middle and front of the cell and changed sign at the sides and posterior corners of the cell (Fig 2B, left‐middle panel). The y‐component of the stress, *T_y_*, was largest in the two posterior corners and was directed toward the midline of the cell (Fig 2B right‐middle panel). We have verified that this traction force map remains qualitatively unaltered when using a different reconstruction method (Appendix Fig S2B) (Butler *et al*, 2002). Furthermore, we found that the location in the posterior corner where. *T*_x_ changed sign corresponded to the location of maximum stress, as indicated by the black dots in the kymographs. This maximum stress occurred at locations of maximum gradient intensity of the fluorescent signal and remained approximately at the same location relative to the cell (Fig 2B). Therefore, both the area and the total force, calculated by integrating the absolute stress within the cell's basal plan, remained roughly constant during the movement of the cell (Appendix Fig S1C–E). The change in the direction of forces can also be seen when integrating the stress *T*_x_ in the direction of motion and plotting it as a function of y (Appendix Fig S3A). Finally, we computed the cell speed as a function of the cell area and total force, and the pressure (force per area) as a function of the cell area. Both quantities were found to be largely independent of the cell area (Appendix Figs S4A and B, and S5A).

The actin distribution of type 2 fan‐shaped cells also revealed a traveling wave with constant shape and speed. There was, however, a subtle difference in type 1 and 2 cells as the type 2 distributions formed a ring that is positioned away from the membrane (Fig 2C, upper row). Consistent with these observations, the LimE‐GFP kymograph did not show any distinct spatial or temporal features (Fig 2C, lower row). The distribution of GFP‐myo was identical to type 1 cells and showed an elevated band parallel to the back of the cell (Fig 2C, upper row). Furthermore, the GFP‐myo kymographs showed a region of high fluorescence at the back of the cell (Fig 2C, lower row), thus indicating a clear symmetry breaking and polarization in the cell.

The difference between the two different types of fan‐shaped cells was most striking when examining the traction force maps (Fig 2B and D). The computed stress map for a type 2 cell reveals two large force poles at the posterior corners (see Fig 2D and for more detail Fig EV1B; see also Movie EV4). At the back of the cell, *T*_x_ was positive, which means forces are in the direction of the motion. However, and in sharp contrast to the pattern for type 1, in the front half of the cell, *T*_x_ was negative, indicating that the force exerted onto the substrate was directed backward (Fig 2D, left‐middle panel). *T_y_* was largest in the two posterior corners and was directed toward the midline of the cell (Fig 2D right‐middle panel). As for the type 1 cell, we also integrated the traction forces in the direction of motion and plotted it as a function of *y* (Appendix Fig S3B) and computed the cell speed as a function of the cell area and total force (Appendix Fig S4A and B). Again, the cell speed was largely independent of these parameters but was found to be smaller than the speed of type 1 cells. Furthermore, the pressure is also independent of the basal area (Appendix Fig S5A).

The kymographs of *T*_x_ and *T_y_* showed that, as is the case for type 1 cells, the two force poles at the posterior corners remained present for the entire duration of migration (Fig 2D, lower row), resulting in a nearly constant area and total force during their migration (Appendix Fig S1C–E). The kymograph of *T*_x_ clearly showed the change in direction of *T*_x_ along the cell outline, occurring at the locations of maximal stress *T* (black dots, Fig 2D). As for type 1 cells, simultaneous measurement of the traction force pattern and the myo‐GFP distribution revealed that this maximum stress occurred at the locations of maximum gradient intensity of myo‐GFP (white dots, Fig 2C). Finally, to verify that our traction force patterns are not affected by the thickness of the gel, which could potentially introduce long‐range effects in bead displacements (Merkel *et al*, 2007), we repeated the experiments for thinner gels (3 vs. 15 μm). For these thin gels, the traction force pattern for type 1 and type 2 cells was qualitatively unchanged (Fig EV1C and D).

To determine whether the morphology of the two types differed, we fitted the basal surface morphologies to an ellipse (Materials and Methods). The major and minor axes as a function of area are shown in Fig 2E, where the different cell type and morphology stability are indicated with different colors. A more detailed graph, indicating the different cell strains and generation methods, is presented in Appendix Fig S6. For both cell types, the major and minor axes of the fitted ellipse increased for increasing basal surface area, but the basal area of type 2 cells was on average larger than that of type 1 cells. Thus, the force pattern seemed to be mostly determined by the cell size and not by the method employed to obtain fan‐shaped cells. In addition, the total force was found to increase for increasing area (Fig 2E, third panel). This dependence of the force on the area has also been observed in migrating keratocytes (Sonoda *et al*, 2016). We also determined the total force in the direction of motion, *F_x_*, and perpendicular to the motion, *F_y_* (see Materials and Methods). A plot of *F_x_* vs. *F_y_* showed a linear dependence with a slope larger than 1, indicating that the forces perpendicular to the direction of motion were larger than in the direction of the motion (Fig 2E, right panel).

### Target waves lead to oscillatory cells

**Figure 3:**
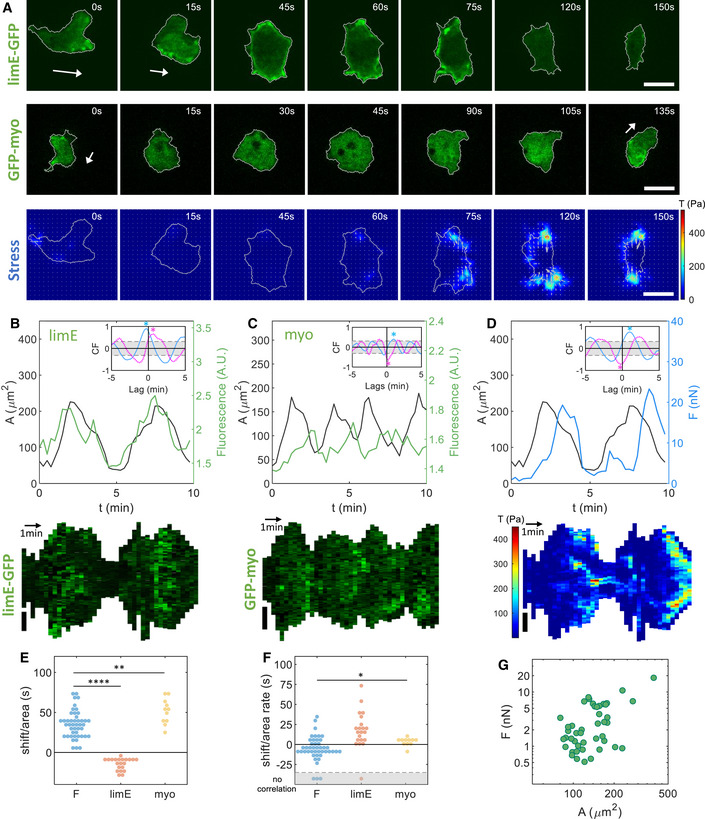
Traction force maps and distributions of signaling components in oscillatory cells Sequence of snapshots of the LimE‐GFP distribution and stress *T* for an oscillatory cell together with snapshots of the GFP‐myo distribution for a separate oscillatory cell. Corresponding speeds are 7.0 and 4.6 µm/min. The sequences show an entire cycle of oscillation (spreading and contraction). White arrows represent the direction of motion before the cell spreads and contracts during which no net motion exists.Top: Basal area (black line) and LimE fluorescent intensity (green line) as a function of time, for the same cell as in (A). Inset: Temporal CF of the area and the LimE fluorescent intensity (blue) and of the area change rate and the LimE fluorescent intensity (magenta). Bottom: Kymograph of the LimE‐GFP intensity along the cell outline.Top: Basal area (black line) and myosin fluorescent intensity (green line) as a function of time for a separate cell. Inset: Temporal CF of the area and the myosin fluorescent intensity (blue) and the area change rate and the myosin fluorescent intensity (magenta). Bottom: Kymograph of the GFP‐myo intensity along the cell outline.Top: Basal area (black line) and total force F (blue line) as a function of time, for the same cell as in (A). Inset: Temporal cross‐correlation function (CF) of the area and *F* (blue) and of the area change rate and *F* (magenta). Bottom: Kymograph of *T* along the cell outline.Median shift in the CF of the area and *F* (33.8 (22.5/42.2) s; *N* = 45 biological replicates), the area and the LimE‐GFP intensity (−11.2 [−19.6/−10.3] s; *N* = 21 biological replicates), and the area and the GFP‐myo intensity (52.5 [41.3/62.8] s; *N* = 11 biological replicates).Median shift in the CF of the area change rate and F (−3.7 [−11.2/3.8] s; *N* = 42 biological replicates), the area change rate and the LimE‐GFP intensity (16.9 [7.5/30.0] s; *N* = 20 biological replicates), and the area change rate and the GFP‐myo intensity (3.8 [0/7.5] s; *N* = 11 biological replicates). Data bellow the dashed line indicate cells for which no significant correlation was found.Time‐averaged total force F as a function of the cell area A. Data information: (B–D) The peaks in the CF are indicated by star symbols, the 95% confidence interval is gray‐shaded, and the sign of the peak in the CF defined whether the quantities were correlated (largest peak occurred for positive CF values) or were anticorrelated (largest peak occurred for negative CF values; see Materials and Methods). (E, F) *P*‐values higher than 0.05 are considered not significant, *P < 0.05, **P < 0.01, and ****P < 0.0001 as determined by the Wilcoxon–Mann–Whitney test using the rank sum function in MATLAB. All scale bars in the figure: 10 μm.

**Figure EV2:**
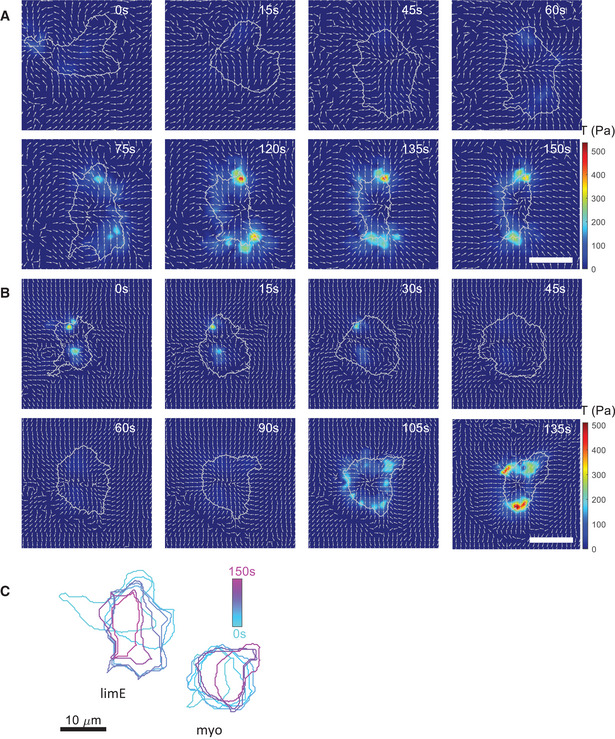
Detailed traction force maps for oscillatory cells Detailed traction force map for the oscillatory cell with LimE‐GFP presented in Fig 3.Detailed traction force map of the second cell of Fig 3, expressing GFP‐myo. Arrows indicate the stress direction, regardless of the magnitude.Outlines of the cells presented in Fig 3, at the corresponding times. Data information: The arrows indicate the traction force. All scale bars in the figure: 10 μm.

The LimE‐GFP distribution corresponding to an oscillatory cell is consistent with an F‐actin wave that was initiated in the basal plane at the start of the spreading phase (Miao *et al*, 2017) (upper row Fig 3A and Movie EV5). This target wave then traveled along the surface of the cell, and the basal plane expanded when it reached the periphery. As we have shown earlier, the actin wave disappeared from the basal plane by moving up on the cell's side (Cao *et al*, 2019a). Snapshots of the GFP‐myo distribution during an oscillatory cycle are presented in the middle row of Fig 3A, which show that the fluorescent intensity decreased when the cell expanded and increased when the cell's area shrank (Movie EV6). The traction force map of an oscillatory cell for one complete cycle shows that throughout the spreading and contraction cycle, the force onto the substrate was pointing inward, toward the center of mass of the cell (see Fig 3A and for more detail Fig EV2A for the LimE‐GFP cell and Fig EV2B for the GFP‐myo cell). Furthermore, the force and stress were higher during the retraction phase than the expansion phase.

The periodic nature of the cytoskeletal waves and basal area size is illustrated in Fig 3B (upper panel), where we plot the area and the average LimE‐GFP fluorescence within the cell outline as a function of time. The area changed more than fourfold during a cycle, while the difference between the maximum and minimum fluorescent intensity was almost twofold. We computed the autocorrelation of the area, which can be well fitted with a damped sinusoidal function, indicating that the area dynamics is strongly periodic (Appendix Figs S7 and S8, Appendix Table S1). Furthermore, the period of this oscillation is not strongly dependent on the time‐averaged basal area (Appendix Fig S7D).

To determine how the cytoskeletal components are correlated with morphology changes, we next computed the correlation function (CF) between the cell area, as well as the area change rate (the time derivative of the area), and the intensity of the fluorescent signals. The area and the LimE‐GFP fluorescence intensity were significantly correlated (blue line and symbol, inset upper panel Fig 3B), and the F‐actin activity was maximal before the cell reached its maximal expansion (Fig 3E and Appendix Table S2). Furthermore, the maximum increase in area occurred before the maximum of LimE (magenta line and symbol, inset upper panel Fig 3B and F). Finally, the average GFP‐myo intensity showed oscillatory behavior with the same period as the area (upper panel Fig 3C). We found a positive median shift between the area and this intensity (Fig 3E), indicating that the maximum of myosin fluorescence intensity occurred after the maximum expansion with a considerable delay. The CF between the area change rate and the fluorescent intensity (magenta line, inset upper panel Fig 3C) revealed that the myosin activity was maximal slightly after the maximal decrease in area (Fig 3F).

As expected, the total force also showed oscillations with the same period as the area and cytoskeletal fluorescent intensities (upper panel Fig 3D). The CFs revealed that the area and the total force were correlated, with the area leading the total force (blue line and symbol, inset upper panel Fig 3D and E), while the area change rate and the total force revealed were anticorrelated (magenta line and symbol, inset upper panel Fig 3D and F). Thus, the total force was maximal slightly before the maximal decrease in area. Finally, the temporal evolution of the force, the area, and the cell‐averaged LimE and myosin are summed up schematically in Appendix Fig S9A.

We also determined the kymographs of oscillatory cells (lower panels Fig 3B–D), which clearly showed the oscillatory nature of the cell area, as the length of the boundary oscillates between a maximum and minimum value. The kymograph of the LimE‐GFP‐labeled cell showed that the fluorescent intensity along the membrane is elevated only during the protrusion part of the cycle (lower panel Fig 3B). Conversely, the kymograph of the GFP‐myo‐labeled cell revealed that myosin was present along the membrane mostly during the contraction but not during expansion (lower panel Fig 3C). The kymograph of the total force along the boundary on the cell in Fig 3D showed periods of high forces, corresponding to contraction, alternating with periods of very low forces, associated with expansion (lower panel Fig 3D). In addition, we computed the time‐averaged total force as a function of the cell area, which revealed that larger cells exert a larger total force (Fig 3G). A more detailed graph, where data points for different cell strains are shown by different symbols, is presented in Appendix Fig S10A.

### Amoeboid cells are associated with unstable waves

**Figure 4:**
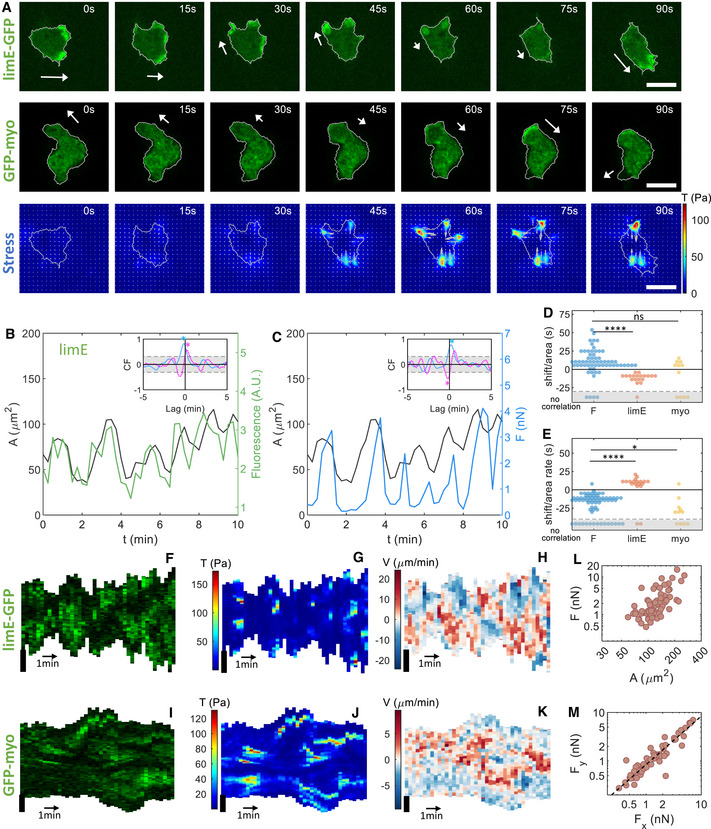
Traction force maps and distributions of signaling components in amoeboid cells ASequence of snapshots of the LimE‐GFP distribution and stress *T* for an amoeboid cell and the GFP‐myo distribution for a separate amoeboid cell. Corresponding speeds are 7.4 and 4.5 µm/min. The sequences show both the phases of protrusion and retraction. White arrows represent the direction of motion.BSpatially averaged LimE‐GFP intensity as a function of time (green line), together with the basal area (black line) for the first cell in (A). Inset: CF of the area and LimE‐GFP intensity (blue) and of the area change rate and the LimE‐GFP intensity (magenta).CBasal area (black line) and total force *F* (blue line) as a function of time corresponding to the first cell in (A). Inset: Temporal cross‐correlation function (CF) of the area and the total force *F* (blue) and of the area change rate and *F* (magenta). The correlation within the shaded region is below the 95% confidence interval.DMedian shift in the CF of the area and *F* (blue symbols, *N* = 66 biological replicates), the LimE‐GFP intensity (brown symbols, *N* = 18 biological replicates), and the GFP‐myo intensity (yellow symbols, *N* = 7 biological replicates). Values of these shifts were determined as 11.3 (3.8/15) s, −11.2 (−15.0/‐7.5) s, and 7.5 (1.9/12.2) s.EMedian shift in the CF of the area change rate and *F* (blue symbols, *N* = 52 biological replicates), the LimE‐GFP intensity (brown symbols, *N* = 17 biological replicates), and the GFP‐myo intensity (yellow symbols, *N* = 7 biological replicates). Data below the dashed line indicate cells for which no significant correlation was found. Shift values are −11.2 (−15.0/‐9.4) s, 11.3 (7.5/11.3) s, and −26.3 (−30.0/‐14.1) s.F–HKymographs for LimE‐GFP intensity, *T*, and edge velocity along the cell outlines for the first cell of (A).I–KKymographs for GFP‐myo intensity, *T*, and edge velocity along the cell outlines for the second cell of (A).L, MTime‐averaged total force *F* as a function of the cell's area and time‐averaged *F_y_* as a function of time‐averaged *F_x_* (slope of fit shown as a dashed line: 0.86, *r* ^2^ = 0.94). Markers indicate the different strains: AX2 LimE‐GFP (>), AX2 GFP‐myo (<), AX2 lifeAct‐GFP (^), and engineered cells (o). Data information: (B, C) The peaks in the CF are indicated by star symbols, the 95% confidence interval is gray‐shaded, and the sign of the peak in the CF defined whether the quantities were correlated (largest peak occurred for positive CF values) or were anticorrelated (largest peak occurred for negative CF values; see Materials and Methods). (D, E) *P*‐values higher than 0.05 are considered not significant, *P < 0.05 and ****P < 0.0001 as determined by the Wilcoxon–Mann–Whitney test using the rank sum function in MATLAB. All scale bars in the figure: 10 μm. Arrows indicate direction of motion.

Consistent with a large body of work (see, e.g., Iwadate & Yumura, 2008), LimE‐GFP appeared as waves close to the membrane that resulted in bright patches located at random locations (upper row Fig 4A and Movie EV7). When these waves reached the membrane, they extended the membrane, creating pseudopods (top row Fig 4A). However, these waves are unstable, are unable to propagate further, and have a limited spatial extent (Miao *et al*, 2017; Cao *et al*, 2019b). As a consequence, the fluorescent intensity of the patches decreased and the pseudopods retracted. The distribution of GFP‐myo in an amoeboid cell also changed as the cell underwent a protrusion and retraction cycle (Movie EV8). During the protrusion of a pseudopod, the fluorescent intensity of GFP‐myo was relatively low and non‐localized (middle row Fig 4A). The retraction of a pseudopod, however, was associated with an accumulation of myosin at the location of the pseudopod (79–90 s; Fig 4A), as observed in previous studies (Iwadate & Yumura, 2008).

**Figure EV3:**
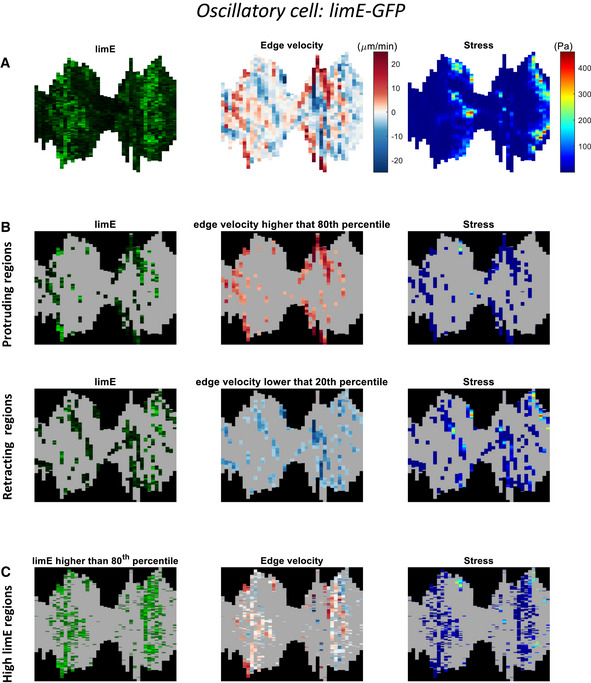
Kymographs of an oscillatory cell expressing LimE AKymographs along an engineered oscillatory cell's outline for the LimE‐GFP intensity, edge velocity, and stress corresponding to the cell expressing LimE in Fig 3A.B, CKymographs as in (A) with masks applied to only display pixels in the protruding and retracting regions (B) or in the region of high fluorescence (C).

The traction force map corresponding to the LimE‐GFP labeled cell showed cycles of expansion due to randomly placed protruding pseudopods (0–30 s), followed by the contraction of these pseudopods (45–90 s; Fig 4A, lower row; for a more detailed map of this cell and of the GFP‐myo‐expressing cell, see Fig EV3). Large stresses only occurred during the contraction phase and were located mainly underneath retracting pseudopods, while the traction forces during the protrusive phase were small. In contrast to the fan‐shaped and oscillatory cells, the forces were transiently associated with each protrusion rather than broadly near the front of the cell. In agreement with previous studies, traction forces were directed inward at all times (Del Alamo *et al*, 2007; Lombardi *et al*, 2007).

The area of the cell presented in Fig 4A showed cyclic changes, with the area changing approximately twofold during a cycle (black curve Fig 4B). The autocorrelation of the area (Appendix Fig S7B) showed that the cell's behavior can be classified as pseudo periodic (see Materials and Methods) with a median pseudoperiod of 2.0 (1.5/3.8) min (*N* = 69), in good agreement with earlier reports (Del Alamo *et al*, 2007; Delanoe‐Ayari *et al*, 2008) (Appendix Fig S7C). The cell‐averaged fluorescence intensity of LimE‐GFP also showed quasi‐periodic dynamics (green curve, Fig 4B) and was significantly correlated with the area (blue line and symbol, inset Fig 4B) with a negative median shift identical to the one found for amoeboid cells (Fig 4D, brown symbols). The area change rate and LimE‐GFP intensity were also correlated (magenta line and symbol, inset Fig 4B) with a positive median shift (Fig 4E, brown symbols). Together, this means that maximal actin polymerization occurs before the cell area has reached its maximum value but after the maximal increase in area.

In contrast to LimE‐GFP, GFP‐myo showed less pronounced localized areas of elevated fluorescence. Therefore, the cell‐averaged GFP‐myo intensity as a function of time was quite noisy for some cells (Appendix Fig S11A) and the CF of the area and the myosin signal displayed a significant correlation in only ~2/3 of the cells (7/11, see Appendix Fig S11B for such an example) with a positive shift (Fig 4D, yellow symbols). In contrast, the area change rate and the fluorescent signal were anticorrelated with a negative shift (Fig 4E, yellow symbols). Thus, on average the peak of myosin fluorescent intensity occurs slightly after the maximum area and before the maximal decrease in area. Comparing the time shift of the CF of the area and force to time shift of the CF of the area and myosin reveals that that the peak of myosin occurs slightly before the peak of force.

The total force as a function of time showed quasi‐periodic dynamics, oscillating between small values during expansion and much larger values during a decrease in the basal area (blue curve Fig 4C). The area and the total force were significantly correlated (inset Fig 4C) with a positive median time shift (Fig 4D, blue symbols), consistent with an earlier study (Delanoe‐Ayari *et al*, 2008). In contrast, the area change rate and the total force were anticorrelated, with a negative median shift (inset Fig 4C and E, blue symbols). Thus, the maximum total force is achieved after the maximum area but before the maximal decrease in area. The temporal evolution of the force, the area, and the cell‐averaged LimE and myosin are schematically summarized in Appendix Fig S9B. Just as for the other cell types, the speed and pressure are largely independent of the time‐averaged cell area and total force (Appendix Figs S4E and F, and S5C).

To gain further insights into how signaling components, force generation, were correlated, we next constructed kymographs of fluorescent intensity, traction force along the membrane, and the cell's edge velocity, defined as the normal velocity of the membrane (Machacek & Danuser, 2006) (Fig 4F, G, I and J). For the cell expressing LimE‐GFP, regions of F‐actin polymerization were observed in the kymograph (Fig 4F). These regions, however, were not colocalized with regions of elevated stress, which were much smaller in extent (Fig 4G). This is in contrast to the cell that expressed GFP‐myo, where patches of GFP‐myo were mostly correlated with regions of high stress (Fig 4I and J). Furthermore, and as expected, a comparison between the fluorescence and edge velocity kymographs revealed that negative edge velocities were associated with high myosin intensity, whereas positive edge velocities corresponded to LimE‐GFP patches (Fig 4H and K).

We then computed the total force, averaged over time, as a function of the cell area (Fig 4L). As expected, since the total force is the integral of the absolute value of stress over the area, it increased for increasing areas. We also determined the time‐averaged total force in the direction of motion, *F_x_*, and perpendicular to the motion, *F_y_* (Materials and Methods). Contrary to the fan‐shaped cells, where the ratio *F_y_*/*F_x_* was close to 2, the ratio *F_y_*/*F_x_* for amoeboid cells is close to 1 (Fig 4M). Thus, the total force in the direction of motion is approximately the same as the total force perpendicular to the motion. This ratio is much smaller than for chemotactic amoeboid cells where the ratio was found to be approximately a half, indicating that the axial stresses, along the direction of motion, are larger than the lateral (Bastounis *et al*, 2014). A more detailed presentation of the data in Fig 4L and M, with different symbols for different strains, is shown in Appendix Fig S10B and C.

### Comparison between the 3 modes of migration

**Figure EV4:**
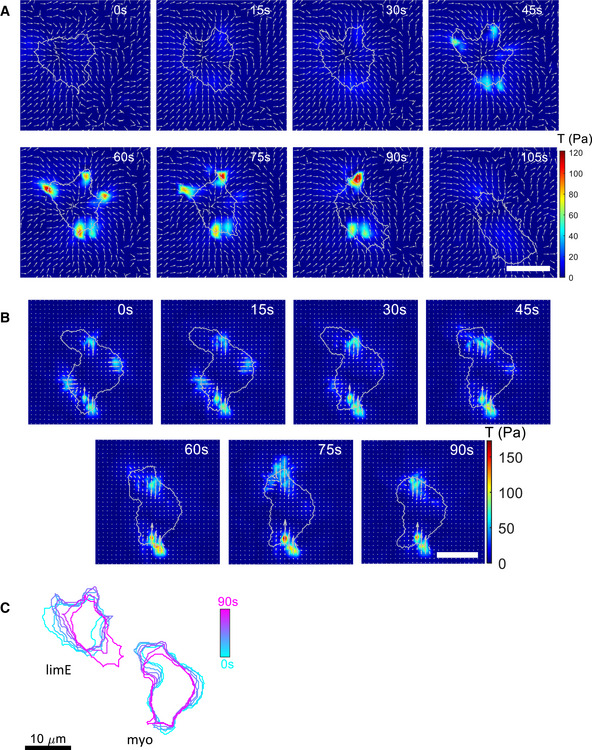
Stress maps of an amoeboid cell Stress maps of the same amoeboid cell as in Fig 4A top row. The arrows indicate only the direction of the stress, regardless of the magnitude. Forces are oriented inward at all times for the amoeboid and oscillatory modes.Stress map of the second cell of Fig 4A, expressing GFP‐myo. The arrows are proportional to the local stress.Outlines of the cells presented in Fig 4, at the corresponding times. Data information: All scale bars in the figure: 10 μm.

**Figure 5:**
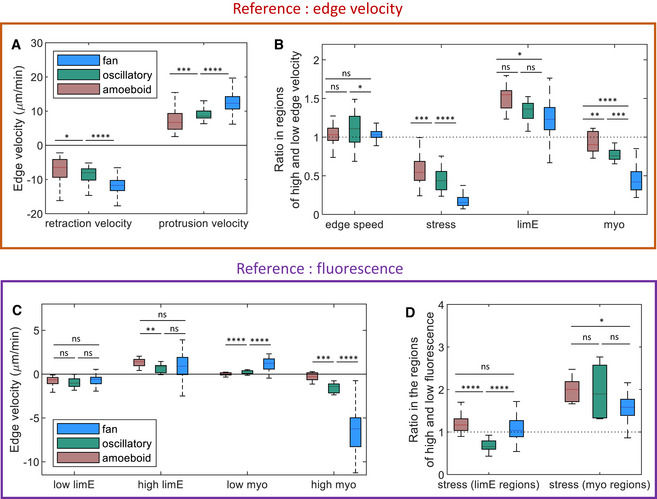
Comparison between different migration modes Protrusion and retraction speed for amoeboid, oscillatory, and fan‐shaped cells, defined as the average of the pixels with the 20% lowest and highest membrane speed.Ratio between the edge velocity, stress, LimE‐GFP, and GFP‐myo intensity in membrane regions identified as protrusions and retractions.Average edge velocity in regions of low and high LimE‐GFP and GFP‐myo fluorescence. High fluorescence was defined as the 20% brightest LimE‐GFP and GFP‐myo pixels in the kymographs, while low fluorescence consisted of the remaining 80% pixels.Ratio between the stress in regions of high and low LimE‐GFP and GFP‐myo fluorescence for the three modes of migration. The ratio was significantly different for all modes and was found to be much smaller in the fan‐shaped cells, which have large traction force poles at the back of the cell. Data information: *P*‐values higher than 0.05 are considered not significant, *P < 0.05, **P < 0.01, ***P < 0.001, and ****P < 0.0001 as determined by the Wilcoxon–Mann–Whitney test using the rank sum function in MATLAB. The box plots were created using the boxplot function in MATLAB, with the line indicating the median, the bottom and top edges of the box indicating the 25^th^ and 75^th^ percentiles, respectively, and the whiskers extending to the most extreme data points not considered outliers. Values and number of biological replicates are listed in Appendix Table S3. (B, C) The dotted line indicates a ratio equal to 1.

Since kymographs include both spatial and temporal information, we computed edge velocity kymographs for all migration modes and correlated them with the force and fluorescent kymographs (Fig EV4 and Appendix Figs S12–S20 and Materials and Methods). We first determined the protrusion and retraction speeds (see Materials and Methods) and found that the fan‐shaped cells exhibited the largest edge velocity for both protrusions and retractions, whereas the amoeboid cells displayed the lowest edge velocity (Fig 5A). Furthermore, for all modes of migration, we found that the protrusion and retraction speed did not differ significantly and that the ratio of their absolute value was close to 1 (Fig 5B and Appendix Table S3). This result is obvious for the fan‐shaped cells, since their morphology does not change, but is less intuitive in the case of the more complex amoeboid morphologies. These results suggest that the speed of retraction and protrusion determine the overall cell speed, which was found to be highest for fan‐shaped cells and lowest for amoeboid cells (Appendix Fig S21).

Next, we computed the ratio between the stress in the protruding regions and the stress in the retracting regions and found it to be smaller than 1 for all modes: The stress in the retracting regions is always larger than in the protruding regions (Fig 5B). The ratio was significantly different for all modes and was much smaller in the fan‐shaped cells (Appendix Table S3). We also computed the ratio of LimE to myosin fluorescence intensity in the protruding and retracting regions (Fig 5B, Appendix Table S3). As expected, LimE‐GFP was brighter in the protruding regions, resulting in a ratio that was larger than 1 for all migration modes. Furthermore, this ratio was smaller than 1 for myosin, indicating that myosin is localized in the retracting regions for all three migration modes (Appendix Table S3). This is especially true for the fan‐shaped cells, where myosin is exclusively localized at the back (retracting) side. For amoeboid cells, on the contrary, a negative edge velocity can sometimes be caused by an active pseudopod at the front that pulls the cell body forward without a marked increase in myosin in the retracting region. As a result, the ratio for amoeboid cells is closer to 1.

We then computed the average edge velocity for the 20% brightest LimE‐GFP and myo‐GFP pixels (high fluorescence regions), as well as the edge velocity for the remaining 80% of the pixels (low fluorescence regions). The average edge velocity was found to be positive in the high LimE fluorescence regions but negative in the remaining low LimE‐GFP fluorescence regions for all three migration modes (Fig 5C, Appendix Table S3). In other words, protrusions occurred predominantly in regions for high LimE‐GFP fluorescence. Conversely, regions of high myosin were associated with negative edge velocities, whereas the remaining regions exhibited near‐zero or positive edge velocities (Fig 5C). Thus, for all migration modes, membrane regions of high LimE‐GFP fluorescence are associated with protrusion and regions of high myosin fluorescence correspond to retractions.

Next, we computed the time‐averaged ratio between stresses in regions of low and high fluorescence (Fig 5D, Appendix Table S3) and found that the computed ratio between stresses in regions of high and low myosin fluorescence was similar and larger than 1 for all three modes. Thus, the stress is higher in regions where myosin is recruited (Fig 5D). The only significant qualitative difference we found was for the stress ratio in regions of high and low LimE‐GFP fluorescence (Fig 5D). For the amoeboid and the fan‐shaped modes, this stress ratio was found to be close to 1. In other words, the stress at membrane locations where F‐actin polymerized was similar to the average stress along the rest of the cell's membrane. For oscillatory cells, however, this ratio was significantly smaller than 1, indicating that the stress at F‐actin polymerization sites was lower than in the remaining sites. This is expected since for these cells, only the expansion, which is associated with low stresses, results in regions of high LimE‐GFP fluorescence. In the case of amoeboid cells, expansion and retraction phases are not well separated as pseudopods are generated randomly in time and space. Consequently, regions of high fluorescence can occur contemporaneously with retracting pseudopods, resulting in a stress ratio close to 1. For the fan‐shaped cells, the high LimE‐GFP region does encompass not only the front but also the part of the boundary close to the two force poles (Fig 3). Thus, even though the stress is low at the front of the cell, the stress ratio is close to 1.

### Computational modeling can explain force patterns

**Figure 6:**
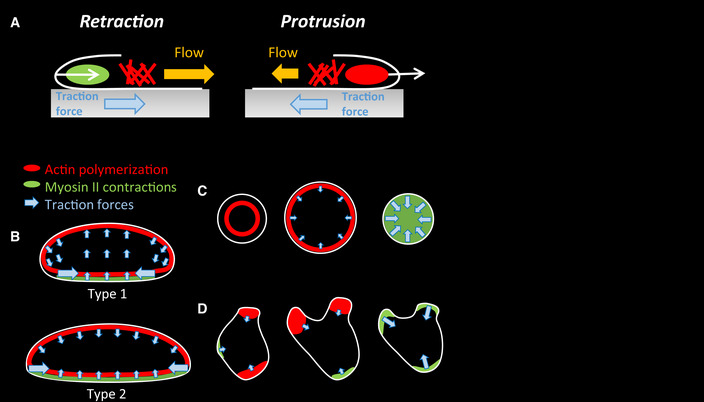
Schematic summary of experimental results ABoth myosin‐mediated contraction and actin‐generated protrusion result in flow of the cytoskeleton and inward‐directed traction force.B–DSchematic representation of the actin (red) and myosin distribution (green), along with the resulting traction force direction (blue arrows with size indicating magnitude) for the three different modes. Shown here are the two different fan‐shaped morphologies (B), and the oscillatory (C) and amoeboid cell (D) at three distinct times.

Our experiments suggest the following scenario, shown schematically in Fig 6. Actin polymerization is responsible for membrane protrusions and is controlled by the wave dynamics: stable waves propagating with the speed of the cell for fan‐shaped cells (Fig 6B), target waves propagating outwardly for oscillatory cells (Fig 6C), and unstable waves in the case of amoeboid cells (Fig 6D). For all migration modes, once an actin wave reaches the cell membrane, it “pushes off” against it, generating a cytoskeletal flow that is directed inward. Due to friction with the substrate, this flow creates traction forces that are also directed inward (Fig 6A). Myosin is responsible for contraction and pulls on the membrane. As a result, traction forces are generated that are also pointing inward (Fig 6B).

For fan‐shaped cells, myosin is along most of the nearly straight membrane at the back of the cell (Fig 6C). Since myosin contracts along this entire band, the traction forces are largest at the end points, located at the rear corners of the cell. The generated cytoskeletal flow created by the contractile myosin and the protrusive actin then leads to the cell‐wide traction force patterns that is different for the two types of cells. Specifically, when myosin is dominant, contractile forces generate a swirling flow pattern and push the cytoskeleton forward in the entire cell (type 1 cell). For type 2 cells, myosin creates forward‐directed flow at the rear while actin polymerization results in backward‐oriented flow at the front. For oscillatory cells, the contractile forces generated by myosin start after the actin ring has moved away from the basal plane, contracting the cell at the basal surface (Fig 6C). Finally, for amoeboid cells, myosin is creating contractions that retract pseudopods, which result in traction forces at the base of pseudopods (Fig 6D).

To test this scenario, we developed a mathematical model with as aim to reproduce the traction force patterns for all three migration modes, including the two fan‐shaped cell types, by simply changing the wave dynamics and spatial location of actin and myosin. In our model, detailed in Materials and Methods, the cytoskeletal interior of the two‐dimensional cell is modeled as a compressible fluid, which is actively driven by actin polymerization, representing protrusion and myosin contraction (Keren *et al*, 2009; Rubinstein *et al*, 2009). The fluid flow interacts with the substrate through friction, resulting in traction forces. Since we are interested in modeling traction force patterns, we do not include any explicit polarization mechanisms. Instead, we specify actin, as visualized using LimE, and myosin distributions and wave dynamics, which allows us to compute the traction force patterns related to a different cytoskeletal organization. Our actin distribution represents freshly polymerized actin, visualized in the experiments using LimE, but we assume that actin filaments are distributed over the entire cell, providing a substrate for myosin. Finally, the cell's morphology and its motion are determined by a force balance equation, which involves membrane tension, cell–substrate friction, and forces due to fluid flow. Our model is implemented using the phase‐field method, which eliminates the need for explicit boundary tracking and which is therefore ideally suitable for so‐called free boundary problem methodology (Lowengrub *et al*, 2009; Shao *et al*, 2010, 2012; Ziebert *et al*, 2012; Moure & Gomez, 2016, 2017; Cao *et al*, 2019a, 2019b, 2019c; Flemming *et al*, 2020; Moreno *et al*, 2020).

**Figure 7:**
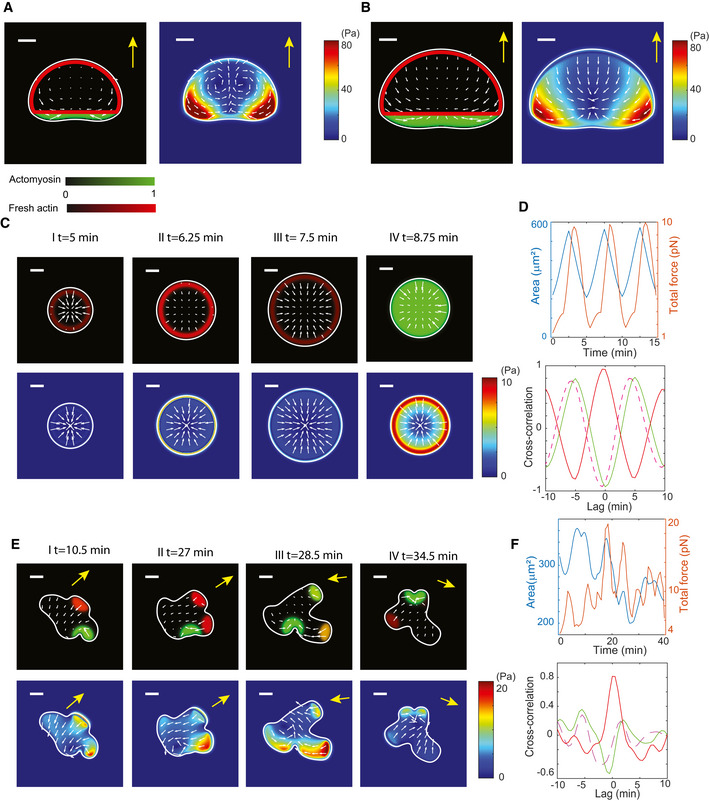
Computation model reproduces experimental results Actin (using a green color scale) and myosin distribution (using a red color scale) in a simulated type 1 cell, obtained for contractile strength parameter *η_m_* = 200 pNμm, with corresponding traction force map. The speed of the cell, and thus the edge speed, was 10.2 μm/min.As in A, but for a type 2 cell. The protrusive strength parameter in this simulation was *η_m_* = 80 pNμm, and the cell speed was 5.9 μm/min.Simulated traction force patterns for oscillatory cells (lower row) together with the corresponding distribution of actin and myosin (upper row). The maximum edge velocity was approximately 4 μm/min.Upper panel: area (blue) and total traction force (red) as a function of time for the computational oscillatory cell. Lower panel: Corresponding correlation function between the area change rate and total force (dashed line), actin (red), and myosin (green).Actin and myosin distribution in a computational amoeboid cell (upper row), along with the resulting traction force patterns and maps for four different times (lower row). The edge velocity of the protrusions was approximately 5.6 μm/min.Upper panel: area (blue) and total traction force (red) as a function of time for the computational amoeboid cell in panel E. Lower panel: Corresponding correlation function (CF) between the area change rate and total force (dashed line), actin (red), and total myosin (green). Data information: White arrows indicate the traction force, and magnitude is displayed using the color map. Yellow arrow indicates direction of motion in all panels. Scale bar in all panels: 5 μm.

We first simulated the motion of fan‐shaped cells. Based on our experimental observations that actin and myosin are spatially excluded and remain fixed over time (Fig 2A and C), we implemented mutually exclusive distributions of actin and myosin that propagate as a stable traveling wave with constant speed. Moreover, since myosin is only elevated near the back of the cells, we take myosin to be restricted to a narrow band at the rear of the cell (Fig 7A and B). In our simulations for the fan‐shaped cells, we kept the parameter that determines the protrusive strength, *η_a_*, fixed, and varied the contractile strength, parameterized by *η_m_*. The values for these and other model parameters can be found in Table EV1.

In a first set of simulations, the contractile strength parameter *η_m_* was chosen to be large, such that the contractile force dominates. The resulting morphology, traction force pattern, and stress pattern are shown in Fig 6A and Movie EV9. The morphology of the cell was consistent with the fan‐shaped cells in the experiments with an arched front and a near‐straight back. Furthermore, the traction force pattern was qualitatively similar to the pattern of a type 1 cell: The largest forces were located in the posterior corners, while the traction forces in the front of the cell were pointing in the direction of motion. Furthermore, as in the experiments (Fig 2B) the traction force pattern displayed two clear rotating vortices, located near these posterior corners, with a sink present at the center of these vortices.

In a second set of simulations, we reduced the value of *η_m_* so that the protrusive force dominates. Decreasing the strength of the contractile force did not change the morphology of the computational cell but resulted in larger cells. Furthermore, this cell showed a distinctly different traction force pattern that was in qualitative agreement with a type 2 cell (Figs 2D and 7B and Movie EV10). The largest forces were still located in the posterior corners, and the maximal forces occurred at locations where both the actin and myosin gradient were large. The traction forces in the front of the cell, however, were now directed opposite from the motion direction. These results suggest that the difference between a type 1 and 2 cell can be explained by the balance between protrusive and contractile forces: Contractile forces dominate in type 1 cells, while protrusive forces dominate in type 2 cells. Consistent with experiments, we found that the speed of type 1 cells was larger than that of type 2 cells (Fig 7). Furthermore, the patterns of stress in the direction of motion *T*_x_ and of the integrated traction forces as a function of y were similar to the corresponding patterns in our experiments (Fig 3B) for both cell types (Appendix Fig S22).

Next, we addressed the traction force patterns in oscillatory cells. Following our experimental results (Fig 3A), we modeled actin to be present within a thin annulus that borders the cell membrane and myosin to be present within the entire computational domain. Both distributions were taken to be spatially homogeneous and were oscillating out of phase. As a result, the cell membrane remained circular and the area was oscillating between a minimum and maximum value (Fig 7C and Movie EV11). We should note, however, that spatially non‐homogeneous distributions that are synchronized in time can also produce oscillatory morphologies that are consistent with the experiments (Appendix Fig S23). The resulting traction forces are consistent with the experimental results: The forces always pointed inward, toward the center of the cell (Fig 7C), and the largest forces were present during the contraction phase (Fig 7D). Furthermore, the computed CFs for these simulations are fully consistent with the experimentally determined CFs (Fig 7D, lower panel).

Lastly, we simulated the traction force patterns arising from amoeboid motion. As in our experiments, we restricted actin polymerization to small, randomly located patches on the boundary, resulting in localized protrusions. Myosin patches accumulated at the pseudopod location in a delayed fashion and in a random location of the cell (Materials and Methods). Simulation snapshots, along with the distribution of actin and myosin and resulting traction forces, show that all forces were pointing inward, consistent with the experiments (Fig 7E and Movie EV12). Also consistent with the experiments (cf. Fig 2), the retraction of pseudopods resulted in larger forces than the protrusion of pseudopods (lower row Fig 7E), and both the area and total force as a function of time showed quasi‐periodic dynamics (Fig 7F, upper panel). Finally, the CFs between the area change rate and total force, actin, and total myosin qualitatively agree with the experimental results (Fig 7F, lower panel).

## Discussion

Our results show that diverse cell migration modes in *Dictyostelium* cells are characterized by distinct traction force patterns and that each of these modes corresponds to specific wave dynamics and spatial distributions of the key cytoskeletal components, F‐actin and myosin. The temporal correlation between the spatially cell‐averaged cytoskeletal components and traction force, however, was conserved across the different modes, suggesting that the modes employ the same migration mechanisms. Furthermore, quantifying the ratio between membrane properties in high and low intensity and edge velocity regions also revealed qualitatively similar results for the three migration modes. The sole exception was the stress ratio in regions of high and low LimE‐GFP fluorescence, which was close to 1 for amoeboid and fan‐shaped cells but significantly smaller than 1 for the oscillatory cells (Fig 5D). We also show that a computational model, which uses the wave dynamics as input and that computes traction forces arising from friction between the cytoskeletal fluid flow and the substrate, is able to reproduce all experimentally observed patterns.

In our TFM experiments, we used relatively thin gel substrates (3–15 µm), with fluorescent beads attached to the top surface. This approach has multiple advantages: gels are stable, do not shrink or swell, and have excellent optical properties. In addition, when all tracer particles are in the same plane, the precision and spatial resolution of TFM are maximized (Driscoll & Danuser, 2015). Furthermore, using thinner gel substrates compared with conventional substrates ensures that substrate deformations resulting from cell traction forces decay over short distances, typically in the order of the thickness of the gel. As a result, the reference (i.e., zero traction force) positions of the tracer particles, which are needed to compute their displacements, can be identified at short distances in front and behind migrating cells, greatly facilitating the dynamic tracking of the traction force distributions along the cell migration trajectory. Most importantly, however, the short decay distance enables the distinction between nearby force foci and, thus, more accurate traction force maps.

Surprisingly, our TFM revealed two distinct traction force patterns for fan‐shaped cells. While both patterns display large forces in the posterior corners of the cells, they differ in their traction force direction at the front of the cell. The force maps for type 2 cells are qualitatively similar to the ones found in migrating keratocytes: two large force poles at the posterior corners, with forces in the front part of the cell pointing opposite from the direction of motion (Fournier *et al*, 2010). In keratocytes, this pattern is believed to be due to the retrograde flow of the protrusive actin network (Fournier *et al*, 2010), which transmits forces to the substrate using adhesive focal adhesion complexes that are formed at the front of the cells, mature, and are released at the back of the cell (Gardel *et al*, 2010). Our results suggest for the type 2 cells the observed pattern is also due to cytoskeletal flow and that the traction force map mimics the flow pattern. Contrary to keratocytes, however, *Dictyostelium* cells do not exhibit stable focal adhesion complexes linked to stress fibers. Like neutrophils, they display transient adhesions marked with paxillin, although a specific integrin–extracellular matrix interaction has not been identified. *Dictyostelium* cells can adhere to a wide variety of surfaces (Bukharova *et al*, 2005; Loomis *et al*, 2012), and it is believed that non‐specific van der Waals and electrostatic interactions play a role (Loomis *et al*, 2012; Tarantola *et al*, 2014). Therefore, it is likely that these forces, together with cytoskeletal flow, provide the required traction forces.

The keratocyte‐like force maps were found in a minority of cells, distinguishable by their larger size. Most cells, however, display a traction force pattern that is at odds with retrograde flow generating traction forces. Specifically, the forces at the front of this type 1 cell point in the direction of motion instead of that in the retrograde direction and the two counter‐rotating vortices are present. This pattern suggests that contractile forces at the back of the cell propel the cell forward. This dominance of contractile forces would also explain why these type 1 cells are smaller than the type 2 cells, where protrusive forces are mostly responsible for motion. Although we have never observed a transition between the two cell types, we cannot rule it out since we can only follow cells for up to approximately 10 min.

For the oscillatory cells, our data suggest a sequence of events that start with an expansion phase during which an actin wave pushes the membrane outward (Cao *et al*, 2019b). As a result, the actin network is being dragged inward, presumably again by retrograde flow (Watanabe & Mitchison, 2002), resulting in traction forces that point toward the center of the cell. The LimE‐GFP intensity reaches a maximum before the maximum area has been achieved, after which accumulation of myosin pulls the membrane inward. Again, the resulting flow of the cytoskeleton network results in inward‐directed traction forces. Since the membrane forces are occurring along the entire membrane, the total traction force is always close to zero. Furthermore, the contraction phase was associated with a peak in traction force, whereas forces were found to be weaker during expansion.

In the case of amoeboid cells, expansion and retraction phases are not well separated or periodic as pseudopods are generated randomly in time and space by short‐lived actin waves with limited spatial extent. Correlating the observed traction force patterns with the actin and myosin distributions, however, allowed us to determine how these cytoskeletal components contribute to morphology changes and locomotion. Since the correlations between both the cytoskeletal molecules and force and the morphology are qualitatively identical to the ones for the oscillatory cell, the migration mode may be described in a similar fashion. Specifically, F‐actin polymerization moves the membrane forward while pushing off against the substrate, generating forces on the substrate that point away from the membrane. Myosin‐mediated contraction occurring at a distant site will then also result in inwardly directed traction forces, which are balanced by the protrusive, actin‐mediated traction forces.

Our numerical model was able to duplicate all observed traction force patterns. As critical input into the model, we used the observed wave dynamics and distributions of actin and myosin. These distributions were then used to generate protrusive and contractile forces, which, together with area conservation and membrane tension, determined the movement and morphology of the cell. Thus, our modeling approach is different from previous studies that solve reaction–diffusion equations to obtain the distributions of signaling components (Cao *et al*, 2019a, 2019b; Moreno *et al*, 2020). However, since these previous studies have demonstrated that the essential wave dynamics of these distributions can be obtained using computational models we are able to use them as inputs (Cao *et al*, 2019a, 2019b; Moreno *et al*, 2020). Future work could include combining these models with the framework we have presented here. A further extension of the model that could potentially verify some of our results is to render cells as three‐dimensional objects, as was carried in recent studies (Cao *et al*, 2019a; Winkler *et al*, 2019). Also note that we have not incorporated the explicit dynamics of adhesion bonds as in some previous results (Shao *et al*, 2012; Reeves *et al*, 2018). Instead, the interior of the deformable computational cell consisted of a compressible viscous fluid, representing the actin cytoskeleton, and the friction of the flow of this fluid with the substrate then generated traction, as in other computational models (Barnhart *et al*, 2015; Allen *et al*, 2020). Note that in this model, just as in some similar (Rubinstein *et al*, 2009; Shao *et al*, 2012), the flow is derived from the cytosolic interior of the cell and not from the membrane (Fogelson & Mogilner, 2014).

The assumption of network friction‐mediated traction in our model is reasonable for *Dictyostelium* cells. Aside from the abovementioned non‐specific cell substrate (Loomis *et al*, 2012), this assumption is also consistent with the flow patterns in the two different types of fan‐shaped cells. In our model, and as a consequence of the friction in our model, the direction of the traction force at a particular location is determined by the direction of the flow at the same location and our simulations predict retrograde actin flow at the front of type 2 cells and more complicated, vortex‐like patterns in type 1 cell. Note that a mechanism in which the membrane is firmly attached to the substrate is unlikely to generate the vortex pattern in type 1 cells. Nevertheless, our results do not rule out additional mechanisms, including adhesion patterns that are dynamically regulated.

By changing the distributions of actin, responsible for protrusive forces, and myosin, responsible for contractile forces, our model was able to recapitulate all traction force patterns. Specifically, for the amoeboid and oscillatory modes, all traction forces were pointed inward. Furthermore, by placing the myosin distribution spatially opposite from the actin distribution in amoeboid cells, it was able to recapitulate patterns observed in the experiments. Finally, by varying the relative strength of the myosin and actin forces, it generated both type 1 and type 2 cells. Taken together, our numerical results suggest that the traction force patterns in *Dictyostelium* cells are primarily due to friction between cytosolic flow and substrate and different patterns are generated by different distributions and wave dynamics of actin and myosin.

## Materials and Methods

### Cells and plasmids

We used wild‐type AX2 cells, amiB‐null AX2 cells, and engineered AX2 cells in our experiments. Wild‐type and amiB‐null were transformed with the plasmid expressing LimE‐delta‐coil‐GFP. Engineered cells were transformed with the plasmid expressing LimE‐YFP. In addition, wild‐type and engineered cells were transformed with the plasmid pBig‐myo, expressing GFP‐myoII and wild‐type cells were transformed with the plasmid pEXP‐4 carrying lifeAct‐GFP.

Wild‐type and fluorescently labeled AX2 cells were kept in an exponential growth phase in a shaker at 22°C in HL5 media. For cells expressing LimE‐GFP, HL5 was supplemented with hygromycin (50 μg/ml), while for cells expressing GFP‐myosinII, it was supplemented with G418 (10 μg/ml). To obtain amoeboid cells, 10^5^ cells were plated on the soft silicone gel substrate used for traction force measurements (see below) in HL5. Recordings started 15 min after plating for up to 3 h. For fan‐shaped cells, cells were diluted to a low concentration (1–2 × 10^5^ cells/ml) to stop exponential growth on the day before the experiment and kept in a shaker at 22°C in HL5 media. After 15–18 h, the cell concentration reached 2–5 × 10^5^ cells/ml and 10^5^ cells were plated in 7 ml DB (5 mM Na_2_HPO_4_, 5 mM KH_2_PO_4_, 200 μM CaCl_2_, 2 mM MgCl_2_, pH = 6.5) on the soft gel substrate. Recording started 4–5 h after plating for up to 3–4 h. Up to 50% of cells prepared in this way were fan‐shaped.

AmiB‐null cells were grown in HL5 in petri dishes and harvested when they reached 50–70% confluency. To obtain fan‐shaped cells, 10^5^ cells were plated in 7 ml DB on the soft gel substrate. Recording started 4–5 h after plating for up to 3–4 h, after which 30–50% of cells were fan‐shaped (Cao *et al*, 2019b).

AX2 cells, including fluorescently labeled ones, were engineered to clamp phosphatidylinositol‐4,5‐bisphosphate (PtdIns(4,5)P2) at low levels, as described previously (Miao *et al*, 2017). Briefly, this was achieved by expressing the yeast PtdIns(4,5)P2‐specific phosphatase Inp54p and recruiting it to the cell membrane through a chemically inducible dimerization system. Cells were grown in HL5, supplemented with hygromycin (50 μg/ml) and G418 (20 μg/ml) in petri dishes and harvested when they reached 50–70% confluency. Vegetative cells carrying mCherry‐FRB‐Inp54p (pB18) and N150‐FKBP‐FKBP (pDM358) were plated on our soft gel substrate in DB. After 15 min, 1.6 μM rapamycin was added and recording was started after an additional 15 min for 4 h. As described before, adding rapamycin results in a sizable fraction of amoeboid cells converting to fan‐shaped or oscillatory motion. Engineered oscillatory cells were also obtained from a second batch of cells expressing mCherry‐FRB‐Inp54p (pB18) and N150‐FKBP‐FKBP/LimE‐YFP (pDM358) and a third batch expressing N150‐Inp54p (pDM358), N150‐FKBPBP (pCV5), and GFP‐myosinII (pBig‐myo).

### Traction force microscopy

As is customary for TFM, cells were plated on a deformable substrate that contained small fluorescent tracer particles (Sabass *et al*, 2008; Style *et al*, 2014). The spatial map of displacements of these particles (relative to their positions with no cells on the substrate) was measured (Appendix Fig S2A) and converted, using computational algorithms, to a spatial map of cell traction forces. Specific details are described in the following.

#### Silicone gels

A thin layer (3 or 15 μm) of soft silicone gel was spread in 50‐mm round dishes with a glass‐bottom coated with 40‐nm fluorescent beads. The exact preparation steps are described below. Young's modulus was measured to be ~1 kPa, using a centrifugal rheometer (Appendix Fig S24).

#### Glass preparation

47‐mm round coverslips from WillCo‐dish® Kit glass‐bottom dishes were cleaned with ethanol and plasma‐treated for 15 s to activate the glass surface. The surface was functionalized with a vapor deposition of (3‐aminopropyl)trimethoxysilane (APTMS) and 3‐(trimethoxysilyl)propylmethacrylate (Sigma‐Aldrich) for 10 min at 170°C. The glass bottom was then assembled into the WillCo petri dish with dedicated sticker. 40‐nm carboxylate‐modified red or yellow‐green fluorescent beads (580/605, Molecular Probe F8793 or 505/515, Molecular probe F8795) were diluted 40,000 times in a buffer with pH 8 (20 μl HEPES/ml, 10 mM NaOH in DI water), and incubated with 0.5 mg/ml 1‐ethyl‐3‐(3‐dimethylaminopropyl)carbodiimide (EDC, Sigma‐Aldrich) on the glass bottom for 2 min before washing with DI water. Dishes were dried for 1 h at 65°C and cooled down before the silicone gel deposition.

#### Functionalized silicone gel deposition

Soft gels were prepared using the curer CY52‐276A and base CY52‐276B (Dow Corning Toray) with a weight ratio 1.2:1.0 (total weight 11 g) to achieve a Young modulus of 1 kPa. The silicone gel was functionalized in bulk with (20–25% aminopropylmethylsiloxane)‐dimethylsiloxane copolymer (APTES‐PDMS, Gelest, Inc.). To delay the viscosity increase, we also used QSIL PLE (Quantum Silicones QSI). 2.5 μl of stock solution containing 10 ml ethanol, 10 μl APTES‐PDMS, and 25 μl QSIL PLE was added for each 1 g of gel. Ingredients were mixed for 3 min using an overhead stirrer (Heidolph RZR1) and centrifuged for 1min to remove bubbles. 500 μl of gel mixture was poured into a glass‐bottom dish and spread with a spin coater for 30 s at 4,000 rpm (for 15‐μm‐thick gels) or for 300 s at 7,500 rpm (for 3‐μm gels). The gel layer was baked for 8 h at 65°C.

#### Surface coating

Each dish was incubated with 40‐nm carboxylate‐modified red or yellow‐green fluorescent beads diluted 1:1,000 in a HEPES buffer with pH 8 for 3 min with 0.5 mg/ml EDC. Excess beads were washed off by carefully flowing DI water over the dish, ensuring that the gel was never exposed to air. 0.3 mg type I collagen (PureCol 3 mg/ml, Advanced BioMatrix) diluted in 2 ml water with 0.5 mg/ml EDC was added to each sample. After 3 min, the solution was washed off with DI water and replaced by DB buffer. Dishes were stored at 4°C for up to 1 week. Gel thickness was measured using confocal microscopy and the two layers of beads. Results were corrected by the ratio of the glass refractive index to the gel refractive index (*n* = 1.4).

### Imaging

DIC and fluorescent images (561‐nm excitation, for the red fluorescent beads, and 488 nm for the GFP probes and yellow‐green beads) were captured every 15 s with a 63× oil objective on a spinning‐disk confocal Zeiss Axio Observer inverted microscope equipped with a Roper Quantum 512SC camera. Autofocus was set on the fluorescent beads at the surface of the gel so that all images were recorded in the basal plane.

### Data analysis

#### Image analysis

To visualize and analyze the cell's surface area, we used the fluorescent data of mCherry‐FRB‐Inp54p, LimE‐GFP, or GFP‐myoII. Alternatively, for non‐fluorescent cells we used DIC images. Pixels within this boundary were detected using a custom MATLAB algorithm, which created a binary image. For fluorescent images, this binarization was performed by applying a threshold automatically determined using the Ridler–Calvard method (Ridler & Calvard, 1978). Then, outlier pixels were removed (using the function bwareaopen), followed by image dilation, the filling of holes, and image erosion. For DIC images, the following steps were performed before binarization: A blurred background was created from images that did not contain the cell. This background was subtracted from the images containing the cell. Shadows from DIC imaging were turned into bright spots by taking the absolute value after subtracting the background. A Gaussian blur was then applied to the images, after which binarization was carried as for fluorescent images.

The binary image was then used as input to the MATLAB function regionprops to determine the basal surface area, average fluorescent intensity inside the basal plane, cell morphology, and fit to ellipse. The cell outline was determined using the MATLAB function bwboundaries, from which we constructed kymographs of fluorescent intensity and forces and computed the cell's center of mass. Cell tracks and cell velocity were determined by tracking the centroid of each cell in each frame.

#### Assignment of migratory modes

Assignment of migratory modes followed the method described by Miao *et al* (2017). Briefly, oscillatory cells were defined as cells that displayed a large coefficient of variation of the area. For the remaining cells, fan‐shaped cells were defined as cells that migrated perpendicular to their long axis. All other cells were defined as amoeboid cells. Less stable fan‐shaped cells were defined as cells that did not keep a constant area (coefficient of variation [COV] > 0.075) or speed (COV > 0.4). For these less stable cells, the force patterns were not clear, making the assignment between types 1 and 2 problematic.

#### Statistics

Experiments were performed on at least two or three different days for each type of cells and for each type of motion. For data that were not normally distributed, data are reported as median (interquartile 1/interquartile 3) and the significance was evaluated with the Wilcoxon–Mann–Whitney test using the rank sum function in MATLAB. *P*‐values higher than 0.05 are considered not significant, * corresponds to 0.05 > *P* > 0.01, ** to 0.01 > *P* > 0.001, *** to 0.001 > *P* > 0.0001, and **** to *P* < 0.0001.

#### Force computation

Bead displacements and force reconstruction were computed using an open source MATLAB algorithm (R2018a; The MathWorks) (Han *et al*, 2015), which is based on the boundary element method (Dembo & Wang, 1999). Beads were tracked by subpixel correlation by image interpolation (SCII), and traction force reconstruction was accomplished using the boundary element method and L1 regularization. Typical bead density detected by our code was 1.2/μm^2^. Resolution of the resulting traction force was approximately 1 μm, and the noise level was about 15 Pa. Total force was defined as the sum of absolute value of all local stresses, *T*(*x*, *y*), multiplied by the local area ∆*A*: *F*_tot_ = ∑|*T*(*x*, *y*)|∆*A*. To capture the entire cell, the cell outline was dilated with the MATLAB function imdilate using a disk of radius 8 pixels as a structural element, resulting in an outline that was approximately about 1.3 μm larger in each direction. The local area depended on the bead density but was approximately 1 μm^2^. Please note that this quantity is not the net force from the cell on the substrate, which can be obtained by summing the vector force field.

In our force maps, cells appear to exert traction forces in areas outside their physical boundaries. This appearance of non‐zero forces outside the cells is due to the finite spatial resolutions of both the tracer particle displacement map and the conversion of the displacement map into the traction force map and is inherent to force reconstruction methods that do not have any constraints on where traction forces are exerted. Thus, unlike some methods explicitly postulating that traction forces are only applied at the adhesion complexes within the cell footprint, e.g., traction reconstruction with point forces (Sabass *et al*, 2008), our procedure will always result in traction force maps with non‐zero forces just outside of the cell footprint. We should also point out that a different computational technique of obtaining the traction force map, the Fourier transform traction cytometry method (FTTC) (Dembo & Wang, 1999), gives qualitatively similar results (Appendix Fig S2B).

#### Rotation of stress maps and fluorescent images

In order to define the stress maps along the direction of motion *T*_x_ and the stress perpendicular to motion, *T_y_*, the stress vectors were rotated for the amoeboid and fan‐shaped modes. The angle of rotation is based on the cell's trajectory obtained from the center of mass coordinates. For the fan‐shaped mode, the trajectory is linear so a single angle can be extracted for the whole trajectory. For the amoeboid mode, however, the trajectory is random and was rotated each time frame. For this, an angle *φ*(*t*) is defined for each frame (time *t*) between the vector connecting the center of mass position at time *t *− 1 and *t* + 1 and the *x*‐axis. A rotation matrix *R*(*t*) is then defined using this angle: $R\left(t\right)=\left[\begin{array}{cc} \cos\left(\varphi \left(t\right)\right) & ‐\sin\left(\varphi \left(t\right)\right)\\ \sin\left(\varphi \left(t\right)\right) & \cos\left(\varphi \left(t\right)\right) \end{array}\right].$

The original measurement obtained from TFM provides us with the components of the traction stress ($T_{x_{0,i}},T_{y_{0,i}}$) measured at position *i* ($x_{0,i},y_{0,i}$ ). These components can be transformed using the rotation matrix to obtain rotated values: $\left(\begin{array}{c} T_{x,i}\\ T_{y,i} \end{array}\right)=R\left(t\right)*\left(\begin{array}{c} T_{x_{0,i}}\\ T_{y_{0,i}} \end{array}\right)$. The position vector ($x_{0,i},y_{0,i}$) can be rotated in a similar fashion. Repeating this for each position *i*, we obtained a rotated stress map. Note that this procedure can be efficiently carried out by a single matrix multiplication. The total force in the direction of and perpendicular to the motion is then defined as *F_x_* = ∑ |*T*_x_ (*x*, *y*)|∆*A* and *F_y_* = ∑ |*T_y_* (*x*, *y*)|∆*A* where the sum is over all points of the stress map.

To obtain kymographs of fan‐shaped cells (e.g., Fig 2), the rotation was also applied to the cell's outlines, using the same rotation matrix and the fluorescent images were rotated using the MATLAB function *imrotate*. The rotated stresses were interpolated on a regular grid with the same resolution as the fluorescent images (a camera pixel: 212 nm). Finally, pixels along the rotated cell's outlines could be extracted from the rotated fluorescent images and from the interpolated stress maps.

### Correlations

#### Temporal correlations

##### Autocorrelations

The autocorrelation function (ACF) for the area was computed in MATLAB, using the function *autocorr*. For the oscillatory mode, the period P of the oscillations was obtained by fitting the area ACF with a damped cosine function *Ae* ^−^ *^t^* ^/^ *^τ^* cos (2*πt*/P) (Appendix Figs S7 and S8). For the amoeboid mode, exhibiting a weakly periodic behavior, a pseudoperiod was extracted from the position of the first peak in the area ACF as no significant result could be obtained using a damped cosine fit. As expected, for oscillatory cells, the position of the first peak of the area ACF and the period obtained from the damped cosine fit give very close results (see Appendix Table S1). Note that the period of oscillation could also be defined using the ACF of the total force or the strain energy. However, the ACF based on the total force or on the strain energy is less well fitted by a damped cosine fit than the area ACF. Therefore, the period of oscillations reported in the main text is based on the area ACF.

##### Cross‐correlations

Cross‐correlation functions (CFs) between area or area change rate and fluorescent signals or total force were computed in MATLAB using the function *crosscorr*. For positively correlated signals, we computed the time shift as the difference between the maximum value of the CF (as these data are positively correlated) and the origin of time (see, e.g., blue line in inset of Fig 4B). The time shift between the area and the fluorescent signal (LimE‐GFP or GFP‐myoII) or the total force was computed as the shift between the maximum value of the CF (as these data are positively correlated) and the origin of time. The correlation between the area change rate and LimE‐GFP was also positive, so we use the definition of the delay. For signals that were anticorrelated, e.g., the area change rate and the total force for amoeboid cells (Fig 4C), this shift was defined as the time difference between the minimum value of the CF and the origin of time. For both the CF and ACF, dashed lines in the plots represent the 95% confidence interval (CI). Correlation is significant only if the CF or ACF has larger values than this interval. The limits of the CI are defined as ± sqrt(2)erf^−1^ (0.95)/sqrt(*L*), with *L* the size of the sample.

#### Spatiotemporal correlation

The second kind of correlation is based on kymographs created from the values of the stress, the fluorescence, and the edge velocity on the cell's boundary. For the correlation using the edge velocity as a reference, protruding and retracting regions refer to pixels of the edge velocity kymograph with values respectively higher than the 80^th^ percentile and lower than the 20^th^ percentile. Once these regions are identified (Appendix Figs S11–S20), the corresponding regions in the fluorescent and stress kymographs are determined. Average values of the fluorescence and of the stress in the protruding and retracting regions are then computed for each cell. The protruding and retracting velocities are defined as the average edge velocity in these areas. For the correlation based on the level of fluorescence, the 20% brightest pixels are selected from the fluorescent kymographs and denoted as high fluorescence regions. The remaining 80% of the pixels define the regions of low fluorescence. The corresponding regions are determined on the stress and edge velocity kymographs, so that their average value in regions of high and low fluorescence can be computed.

##### Fan‐shaped cells

We computed the cell's edge velocity for type 1 and 2 cells expressing LimE (Appendix Figs S12 and S13) and myosinII (Appendix Figs S14 and S15), and for type 2 cells lifeAct‐GFP‐expressing cells (Appendix Fig S16). Using the kymographs, we further quantified the correlation between membrane‐localized cytoskeletal components, force generation, and motion for fan‐shaped cells by identifying regions of large positive and negative edge velocities, corresponding to retracting and protruding regions. For fan‐shaped cells, these regions obviously correspond to the back and front of the cell, respectively. We also found, and consistent with our observation of large forces in the posterior corner, that the ratio between the traction forces in the protruding (front) and retracting (back) regions was ~0.15. Furthermore, and as expected, LimE‐GFP was brighter in the protruding regions, where F‐actin is polymerizing, whereas myosin was significantly brighter in the retracting regions. In addition, experiments performed with cells tagged with lifeAct‐GFP showed no noticeable difference in fluorescence between retracting and protruding regions, indicating the presence of F‐actin everywhere along the membrane.

Then, we used the fluorescent kymographs to detect regions of high F‐actin polymerization and high myosin activity and correlated them with the stress at the boundary and the edge velocity (Appendix Fig S16E and F). As expected, regions of high LimE‐GFP intensity corresponded mostly to the front of the cell and to higher edge velocity, whereas regions of high myosin activity were found mostly in the back of the cell and were correlated with negative edge velocities (Appendix Fig S16E). Comparing the average values of the stress in the regions of high fluorescence to the values in the rest of the cell revealed that for LimE‐GFP, this ratio was close to 1 (Appendix Fig S16F). For GFP‐myo and lifeAct‐GFP, this ratio was larger than 1. These results suggest that myosin was responsible for most of the total force developed by the cells during motion, and that the forces created by actin polymerization were not significantly larger than the average force in the rest of the cell's outline, including regions of zero normal motion.

##### Oscillatory cells

Next, we quantified the edge velocity of the oscillatory cells displayed in Fig 3, which showed that the edge velocity was high when the LimE‐GFP intensity is highest (Fig EV3). During these expansion phases, the stress was relatively low. When the edge velocity was small, corresponding to retractions, the myosin intensity was high (Appendix Fig S17). Thus, protrusions are associated with increased LimE activity near the membrane, while contractions, and larger stresses, occur when myosin is elevated near the membrane.

We also determined the ratio between the retracting and protruding velocity (Appendix Fig S17D). As for fan‐shaped cells (Appendix Fig S16D), this ratio was close to 1, indicating that the edge velocity during retraction and protrusion was approximately identical. The ratio between the traction forces in the protruding and retracting regions was ~0.4, again illustrating that the traction forces during the retraction phase were larger (Appendix Fig S17D). We also found that LimE‐GFP was brighter in the protruding regions for both engineered and wild‐type cells, while myosin was brighter in the retracting regions (Appendix Fig S17D). The fluorescent kymographs revealed that regions of high LimE‐GFP intensity corresponded mostly to the protruding phase of the cell and that regions of high myosin activity were found mostly during the retractile phase of the cell (Appendix Fig S17E). The ratio between the average stress in the regions of high LimE fluorescence and in the rest of the cell was slightly less than 1 for both engineered and wild‐type cells. This ratio for myosinII fluorescence, on the contrary, was almost 2 (Appendix Fig S17F). Therefore, and similar to fan‐shaped cells, myosin was responsible for most of the total force developed by the cells during morphology changes.

##### Amoeboid cells

We used the velocity kymographs to identify regions of large positive and negative edge velocities (Appendix Figs S18–S20). We found that, on average, the magnitude of the most negative and the most positive edge velocity was the same, indicating that the protrusion and retraction speed were similar (Appendix Fig S20D). We then computed the average fluorescent intensities and average stress in these regions. This revealed that the average stress in the retracting regions was about twofold larger than the stress in protruding regions (Appendix Fig S20D). These findings are consistent with our cell‐averaged results and previous results and demonstrate small forces underneath expanding pseudopods but larger ones in retracting areas (Del Alamo *et al*, 2007; Delanoe‐Ayari *et al*, 2008; Iwadate & Yumura, 2008). Furthermore, and as expected, LimE‐GFP was brighter in the protruding regions, whereas myosin was slightly brighter in the retracting regions. Interestingly, experiments performed with cells tagged with lifeAct‐GFP showed no noticeable difference in fluorescence between retracting and protruding regions, indicating that F‐actin is required for both retractions and protrusions (Appendix Fig S20D). This suggests that myosin and actin can form an actin–myosin complex that is responsible for contraction not only in pseudopods but also in regions distinct from pseudopods.

We also used the fluorescent kymographs to detect regions of high F‐actin polymerization and high myosin activity and correlated them with the stress at the boundary and the edge velocity (Appendix Fig S20E and F). As expected, regions of high LimE‐GFP intensity corresponded mostly to positive edge velocities, whereas regions of high GFP‐myo and lifeAct‐GFP intensity were correlated with negative edge velocities (Appendix Fig S20E). To further quantify this observation, the average values of the stress in the regions of high fluorescence were compared with the values in the rest of the cell (Appendix Fig S20F). For GFP‐myo and lifeAct‐GFP, this ratio is close to 2, while for LimE‐GFP, it is close to 1. This suggests that myosin was responsible for most of the total force developed by the cells during motion, which occurs during retraction, and that the forces created by actin polymerization were not significantly larger than the average force in the rest of the cell's outline, which includes regions of vanishing normal motion.

### Computational model

We propose a mathematical model to explain the different force patterns observed in the experiments. Here, we consider a 2D cell that interacts with the substrate. In our model, the interior of the cell, which is assumed to be the cell cortex, is modeled as a compressible fluid. This fluid is actively driven by actin polymerization and myosin contraction, and the cell morphology and motion are determined by the force balance on the cell boundary. Furthermore, the size of the cell is taken to be constrained within a certain range and the distribution of actin and myosin is pre‐set based on experimental observations. Finally, the friction of the fluid with the substrate generates the traction forces exerted by the cell onto substrate (Barnhart *et al*, 2011). This is a reasonable assumption since *Dictyostelium* cells exhibit non‐specific cell–substrate adhesion and do not utilize focal adhesion complexes (Loomis *et al*, 2012).

To simulate the motion, we utilized the phase‐field approach (Shao *et al*, 2010, 2012; Cao *et al*, 2019c; Moreno *et al*, 2020). In this approach, the shape of the cell is tracked by a phase‐field variable *φ*, with *φ* = 1 indicating the interior and *φ* = 0 representing the exterior of the cell. The cell boundary is then implicitly tracked by *φ* = 1/2. ′The cell shape evolves according to the equation:

$$\frac{d\varphi }{\mathrm{dt}}=‐u\cdot \nabla \varphi +\Gamma \left(\frac{\in \nabla^{2}\varphi ‐G^{\prime }\left(\varphi \right)}{\in +\in c\left|\nabla \varphi \right|}\right),$$

where *u* is the velocity field of the fluid flow, *ɛ* is the phase‐field boundary width, *c* = −***∇*⋅(*∇φ***/| ***∇φ*** |) is the local interface curvature, *Γ* is a relaxation coefficient, and *G*(***φ***)=18 ***φ*** ^2^ (1 − ***φ***)^2^.

The dynamics of the interior fluid is modeled using the Stokes equation:

$$\nabla \cdot \left[\nu \varphi \left(\nabla u+\nabla u^{T}\right)\right]+F_{\text{mem}}+F_{\text{area}}+F_{\text{act}}‐\xi u=0.$$

Here, *ν* is the fluid viscosity, *F*_mem_ is the cell membrane tension, given by *F* = *δH*(*φ*)/*δφ*, with *H*(*φ*) = *γ*(*ɛ* |*φ*|^2^ + *G*(*φ*)/*ɛ*), *γ* is the cell membrane tension per length, and *F*_area_ is the area conservation force that constrains the cell size *A* = ∫*φdxdy* between [*A*_min_, *A*_max_]. Specifically, we take *F*_area_ = *M*_s_ *g*(*φ*)*∇φ*, where *g*(*φ*) = *A* − *A*_min_ if *A* < *A*_min_ or *A* − *A*_max_ if *A* > *A*_max_, and *g*(*φ*) = 0 otherwise and where *M_s_* is a parameter that controls the penalty for having an area outside the preferred range.

The active force in our model is provided by actin polymerization and myosin contraction. Following earlier work (Shao *et al*, 2012), the active force takes the form:

$$F_{\text{act}}=\nabla \cdot \left(‐\eta_{a}\rho_{a}\in {\left|\nabla \varphi \right|}^{2}nn+\eta_{m}\rho_{m}\varphi \right),$$

where *ρ_a,m_* is the density of actin and myosin, respectively. In this equation, the parameters *η_a_* and *η_m_* describe the strength of actin polymerization and myosin contraction and *n* = −*∇φ*/|*∇φ*| is the outward normal direction at the cell membrane. To model the different cell migration modes, we implemented three different spatial distributions.

1. For fan‐shaped cells, we implemented a stationary distribution for both actin and myosin. Since our experiments showed that LimE and myosin are spatially excluded and that myosin was localized in the back of the cell, we restricted myosin to a narrow band at the back of the cell: *ρ_m_* = 1 when *x* − *x_c_* < *β*, where *x* is the coordinate in the direction of motion, *x_c_* is the center of mass of the cell, and *β* is a negative constant. Actin is filled in a ring with width of 2 μm that surrounds the rest of the cell.
2. In the experiments, the oscillatory cells showed spatially homogeneous and temporally oscillating actin and myosin profiles near the cell periphery. In the models, we thus define an annulus with radius *r*_0_, located at the membrane, in which actin shows oscillations. Specifically, we set $\rho_{a}=\zeta \left(r,r_{0}\right)\left[1+\sin\left(\frac{2\pi t}{T}\right)\right]/2\;\text{if}\;\sin\left(2\pi t/T\right)>0$ and *ρ_m_* = 1 if *sin* (2*πt*/*T*) < 0, where *T* is a constant period, and *ζ*(*r*, *r*_0_) = 0 if the distance to the center of mass *r* < *r*_0_, and *ζ*(*r*,*r*_0_) = 1 otherwise. To account for spatial heterogeneity in oscillatory cells (Appendix Fig S23), we introduced two patches of actin along the membrane, using the same method as in the amoeboid cell simulations, detailed below. These actin patches are synchronized, and both actin and myosin have the same oscillatory dynamics as described above.
3. For amoeboid cells, we implemented spatiotemporally heterogeneously distributed actin and myosin. Specifically, and based on our experimental results, we assumed that actin polymerization and myosin are limited to two small protrusions (denoted by *χ_a_* and *χ_m_*) with radius *r*_2_ close to the membrane, and show alternating oscillations. An additional myosin patch was generated in random positions within the cell. Furthermore, to capture the limited lifetime and spatial extend of a pseudopod, we assumed that both actin and myosin had a lifetime *τ*, where *τ* is drawn from a normal distribution with mean of *T* and variance *σ*. Explicitly, for the protrusions, we used the following distributions: $\rho_{a}=\chi_{a}\left(r,r_{1}\right)\left[1+\sin\left(\frac{2\pi t}{T}\right)\right]/2\;\text{if}\;\text{sin}\left(2\pi t/T\right)>0$ and $\rho_{m}=\chi_{m}\left(r,r_{1}\right)\left[1‐\sin\left(\frac{2\pi t}{T}\right)\right]/2\;\text{if}\;\sin\left(2\pi t/T\right)<0$. Here $\chi_{m}\left(r,r_{1}\right)=\left\{1+\tan h\left[3\frac{r_{2}‐\left|r‐r_{1}\right|}{\in }\right]\right\}/2$ is a disk with a center position *r*_1_ that is randomly drawn from the boundary points of the cell, and *χ_m_* = *χ_a_* (*r*,*r_m_* − *r*_1_), with *r_m_* being the cell mass center. For the myosin patch, we simply use $\rho_{m}=\chi_{m}\left(r,r_{1}\right)\left[1‐\sin\left(\frac{2\pi t}{T}\right)\right]/2$.

Parameters for our simulations are given in Table EV1. The equations were solved on a *n* × *m* regular grid with size *L_x_* × *L_y_*. We denote the state of the system at time *t* = *n*Δ*t* by *φ* ^(^ *^n^* ^)^, *u* ^(^ *^n^* ^)^. The *φ*‐equation was solved by forward Euler scheme:

$$\varphi^{\left(n+1\right)}=\varphi^{\left(n\right)}‐\Delta tu^{\left(n\right)}\nabla \varphi^{\left(n\right)}+\Delta t\Gamma \left[\frac{\in \nabla^{2}\varphi^{\left(n\right)}‐G^{\prime }\left(\varphi^{\left(n\right)}\right)}{\in +\in c^{\left(n\right)}\left|\nabla \varphi^{\left(n\right)}\right|}\right],$$

where *∇φ* ^(^ *^n^* ^)^,*∇* ^2^ *φ* ^(^ *^n^* ^)^ are calculated by the Fourier transformation method, and the curvature term is calculated with a central difference scheme.

The Stokes equation was solved with a semi‐implicit Fourier spectral scheme to obtain *u* ^(^ *^n^* ^+ 1)^. To do so, we first subtract the term 2*ν∇* ^2^ *u* from both sides of the Stokes equation to yield

$$\xi u‐2\nu \nabla^{2}u=\nabla \cdot \left[\nu \left(\varphi ‐2\right)\nabla u+\nu \varphi \nabla u^{T}\right]+F_{\text{mem}}+F_{\text{area}}+F_{\text{act}}\equiv RHS\left(u,\varphi \right).$$

We solve the above equation iteratively using the Fourier spectral method,

$$u^{\left(n+1\right)}=F^{‐1}\left[F\left(RHS\left(u^{\left(n\right)},\varphi^{\left(n\right)}\right)\right)/\left(\xi +2\nu k^{2}u_{k}\right)\right]$$

where *u_k_* is the *k*‐th Fourier series, and *F*, *F* ^−1^ is the forward and reverse Fourier transformation, respectively. The iteration will stop until $\max\left|u^{\left(n+1\right)}‐u^{\left(n\right)}\right|<0.01\max u^{\left(n\right)}|$ or the maximal iteration steps exceed 100.

## Author contributions

W‐JR designed and coordinated the project. EG performed the experiments. YC carried out the simulations. EG and AG developed the TFM substrates. YM and PD provided strains. EG, YC, PD, and W‐JR analyzed the data. EG, YC, and W‐JR wrote the draft of the manuscript and edited the manuscript with input from YM and PD.

## Conflict of interest

The authors declare that they have no conflict of interest.

## Supporting information



Appendix

Expanded View Figures PDF

Table EV1

Movie EV1

Movie EV2

Movie EV3

Movie EV4

Movie EV5

Movie EV6

Movie EV7

Movie EV8

Movie EV9

Movie EV10

Movie EV11

Movie EV12

## Data Availability

The datasets of the images in this study are available in the following database: https://doi.org/10.6084/m9.figshare.16826740. Computational code is deposited on https://github.com/Rappel‐lab/Traction_force.
