# Supplementary material for: Coupling traction force patterns and actomyosin wave dynamics reveals mechanics of cell motion
Source: Mol Syst Biol. 2021 Dec 13;17(12):e10505. doi: 10.15252/msb.202110505 (PMC8666840; doi:10.15252/msb.202110505)
Supplement: Supplementary file 1 — Appendix [file MSB-17-e10505-s006.pdf]

Appendix – Table of contents

Appendix Figure S1: Snapshots of lime-GFP, GFP-myo and lifeAct in fan shaped cells; total force F and fluorescence as a function of time for the type 1 and type 2 fan-shaped cells
Appendix Figure S2: Beads displacement and comparison of FTTC and BEM for the traction force reconstruction
Appendix Figure S3: Force fx and fy as a function of y for the type 1 and type 2 cells in Fig. 2
Appendix Figure S4: Speed of the fan shaped, oscillatory and amoeboid cells as a function of area and total force
Appendix Figure S5: Pressure of the fan shaped, oscillatory and amoeboid cells as a function of the area
Appendix Figure S6: More detailed representation of Fig. 2E.
Appendix Figure S7: Period and pseudo-period of oscillation of the oscillatory and amoeboid cells
Appendix Figure S8: Period of oscillation of two oscillatory cells over a long recording time
Appendix Figure S9: Schematic summary of the cross-correlations for oscillatory and amoeboid cells
Appendix Figure S10: More detailed representation of Fig. 3G.
Appendix Figure S11: Basal area and GFP-myo intensity as a function of time of two amoeboid cells
Appendix Figure S12: Kymographs of the LimE-GFP intensity, edge velocity, and stress of a type 1 fan-shaped cell
Appendix Figure S13: Kymographs of the LimE-GFP intensity, edge velocity, and stress of a type 2 fan-shaped cell
Appendix Figure S14: Kymographs of the GFP-myo intensity, edge velocity, and stress of a type 1 fan-shaped cell
Appendix Figure S15: Kymographs of the GFP-myo intensity, edge velocity, and stress of a type 2 fan-shaped cell
Appendix Figure S16: Kymographs of the lifeAct intensity, edge velocity, and stress of a type 1 fan-shaped cell
Appendix Figure S17: Kymographs of the GFP-myo intensity, edge velocity, and stress of an oscillatory cell
Appendix Figure S18: Kymographs of the LimE-GFP intensity, edge velocity, and stress of an oscillatory cell
Appendix Figure S19: Kymographs of the GFP-myo intensity, edge velocity, and stress of an amoeboid cell
Appendix Figure S20: Kymographs of the lifeAct intensity, edge velocity, and stress of an amoeboid cell
Appendix Figure S21: Translational cell speed computed from the cell center of mass for amoeboid, fan-shaped and oscillatory cells.
Appendix Figure S22: Stress in the direction of motion for simulated fan-shaped cells.
Appendix Figure S23: Simulated traction force patterns for oscillatory cells with non-homogeneous signaling distributions.
Appendix Figure S24: Quantification of Young's modulus.

Appendix Table S1: (Pseudo-)period of oscillations determined using different auto-correlation functions for different migration modes
Appendix Table S2: Time shifts in cross correlation functions for different migration modes
Appendix Table S3: Cell speed, edge velocity, and relevant ratios for the three different migration modes

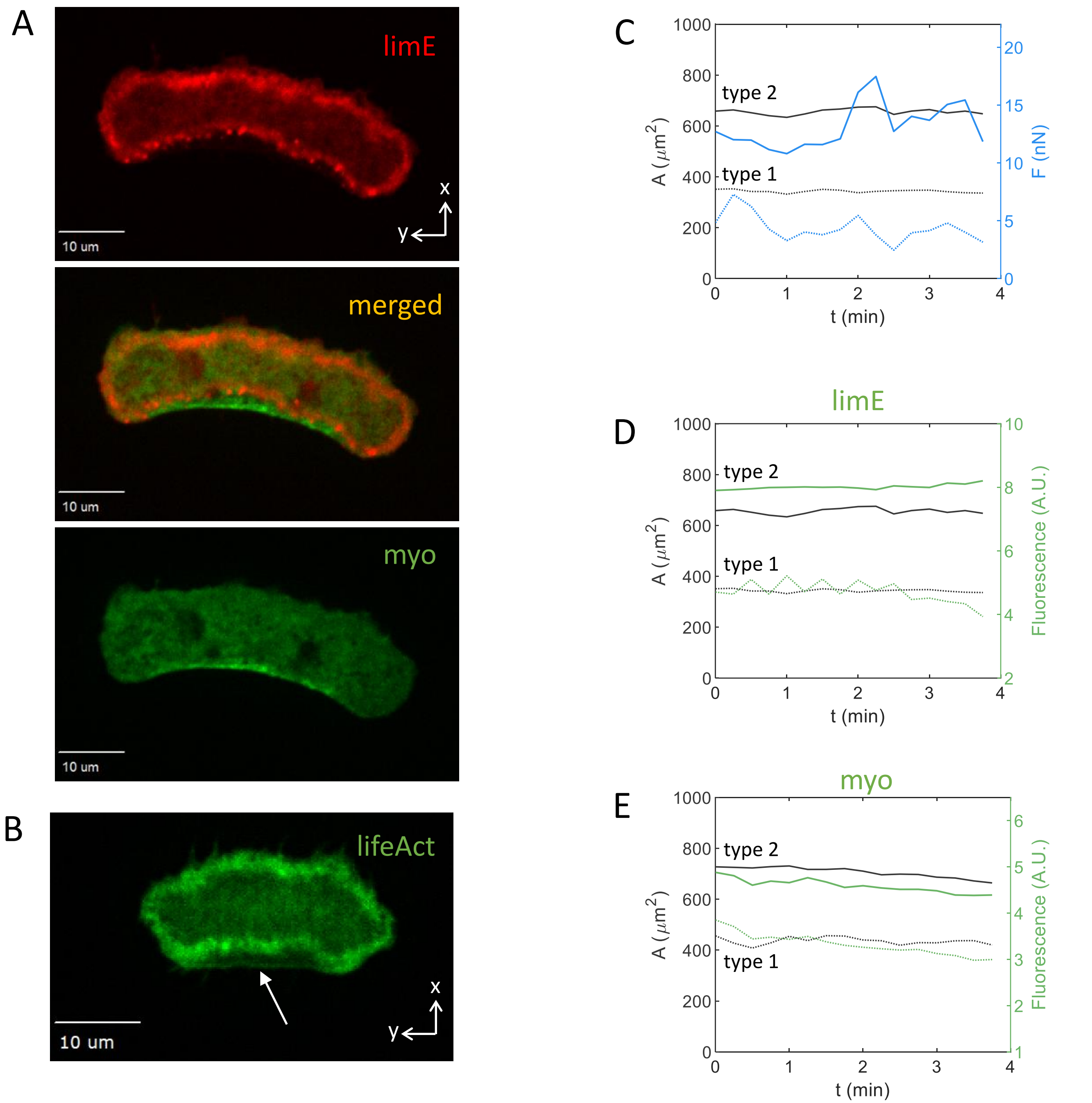

Appendix fig. S1: A. Snapshots of a fan shape cell co-expressing LimE-RFP and GFP-myo. Myosin localizes in the back of the cell in a region free of LimE and the LimE ring detaches from the membrane at the two ends of the myosin region. **B.** Snapshot of a type 1 fan shape cell expressing lifeAct-GFP. In addition to a ring, similar to the distribution of freshly polymerized F-actin, lifeAct-GFP is present in the back of the cell (white arrow). **C-D.** Area, total force and limE intensity of the cell as a function of time for the same cells as in Fig. 2A, B & E. **E.** Area and myosin intensity of the cell as a function of time for the same cells as in Fig. 2D. Dashed lines in (C), (D) & (E) are cells type 1 and solid lines are cells type 2.

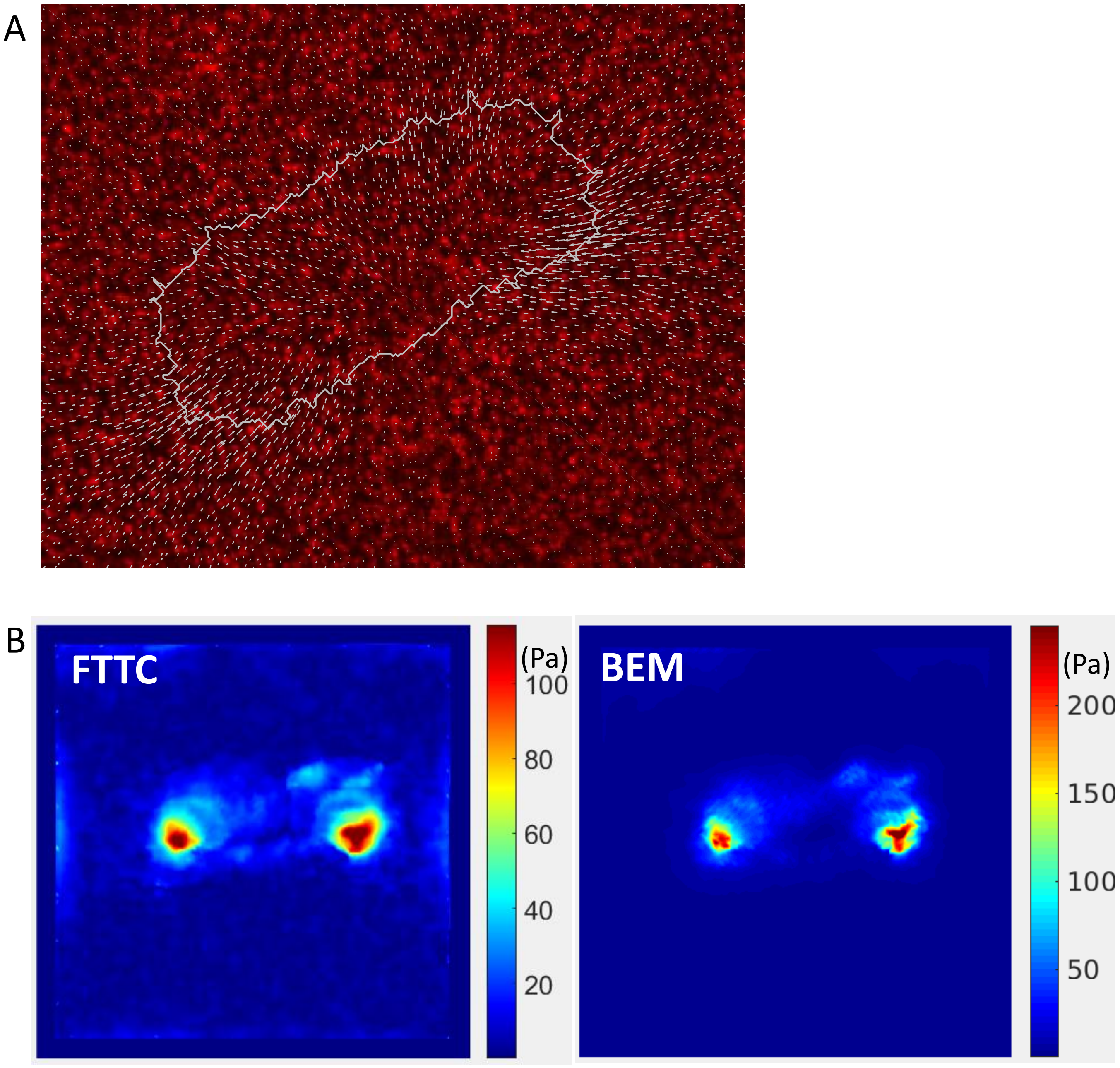

Appendix fig. S2: A: Snapshot of the fluorescent beads corresponding to the TFM map of a fan-shaped cell. Arrows indicate the beads displacement measured with the particle tracking method and sub-pixel correlation by image interpolation (see Methods). Maximum bead displacement in this snapshot is approximately 330 nm. **B**. Comparison of the traction force map obtained using two methods for the force reconstruction: Fourier Transform Traction Cytocymetry (FTTC) with L2 regularization and the Boundary Element Method (BEM) with L1 regularization. The two maps were computed from the same beads displacements. Though the overall map looks similar, the stress magnitude is underestimated with FTTC and noise in the background is lower with BEM and L1 regularization.

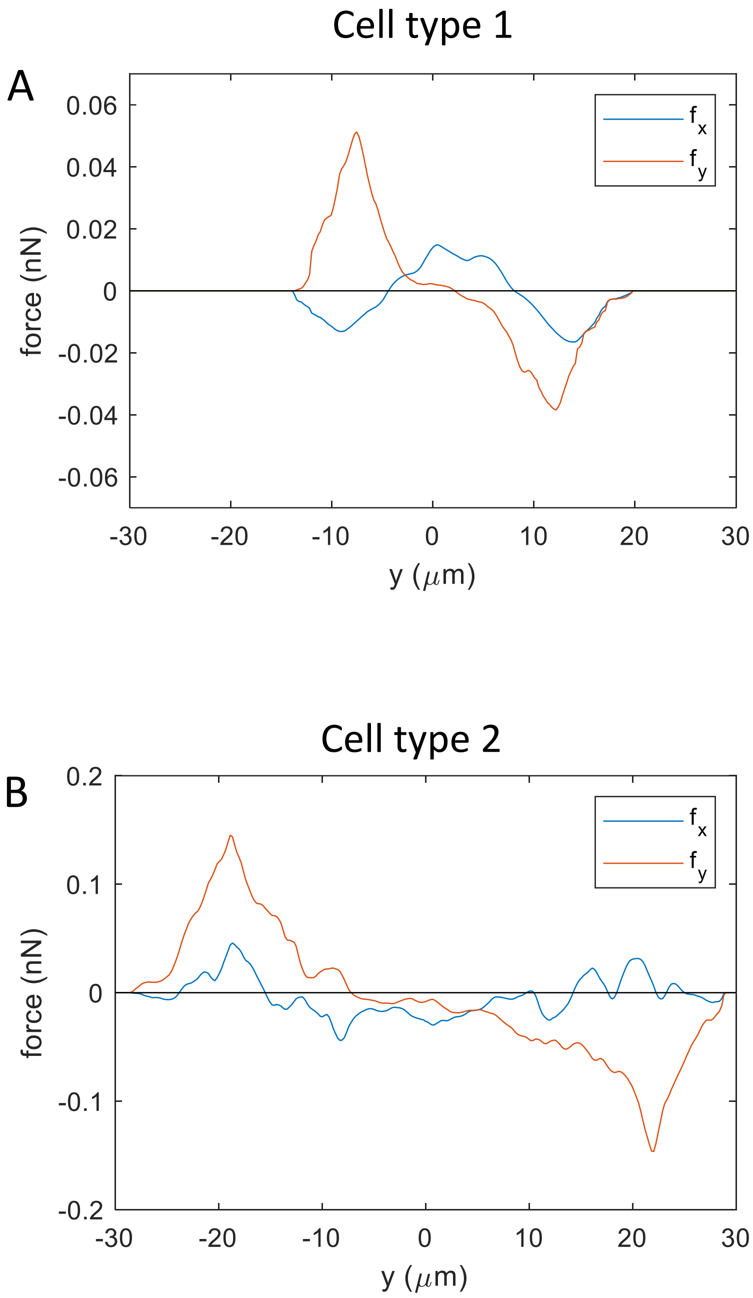

Appendix fig. S3: A&B: Forces f_x and f_y, integrated along x (direction of motion), as a function of y for the cell type 1 (A) and type 2 (B) displayed in fig. 2 B&D. Please note these forces are the sum of the relative value of the stress, while F_x and F_y reported in the main figures are based on the absolute values of the stresses.

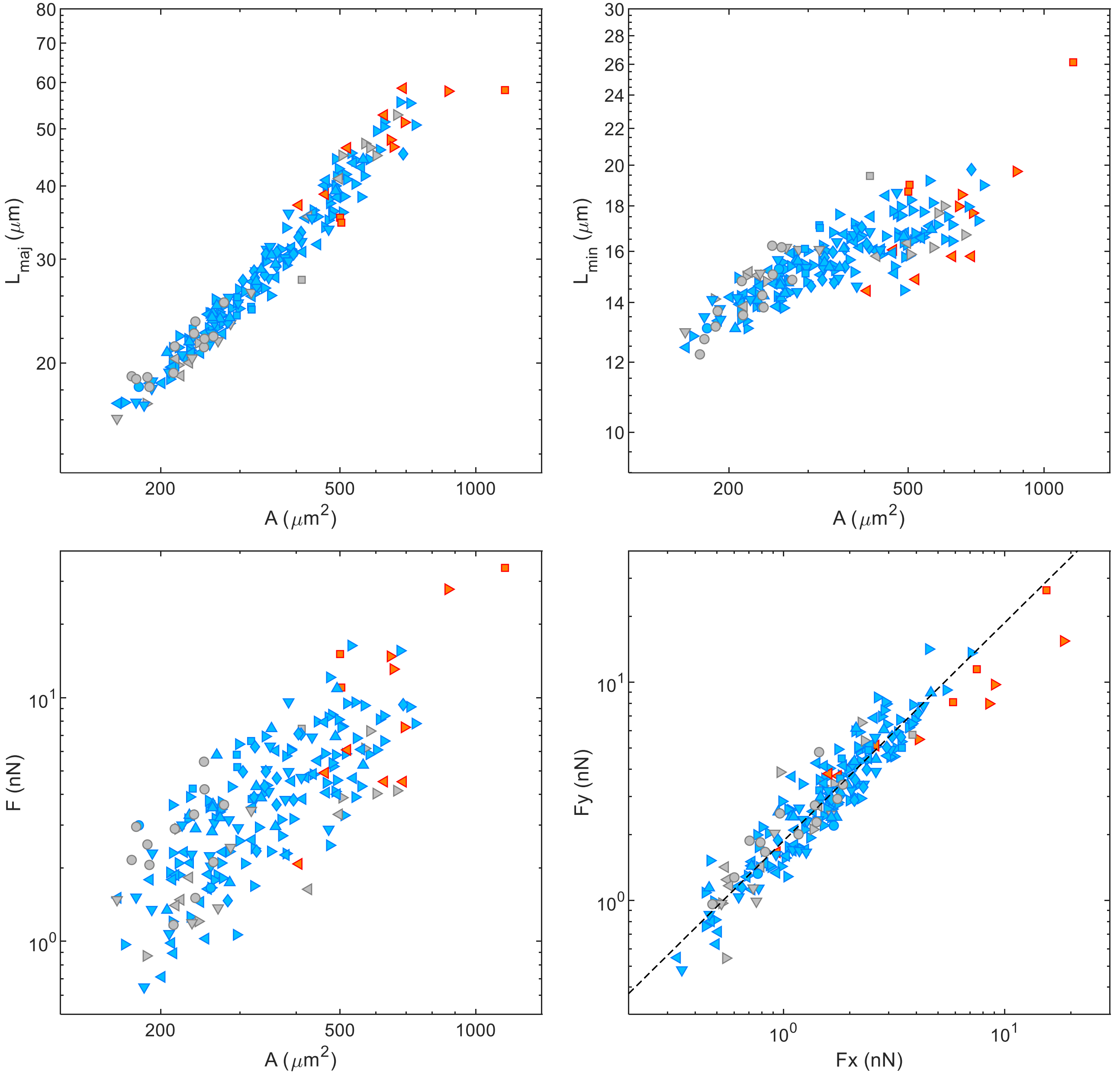

Appendix fig. S4: Larger magnification of Fig. 2E. Cell type 1: blue markers, cell type 2: orange markers, less stable cells: gray markers (see Methods). The shape of the marker denotes the different strains: AX2 (v), AX2 LimE-GFP (>), AX2 GFP-myo (<), AX2 lifeAct-GFP (^), AX2 amiB- (square), AX2 amiB-/LimE-GFP (diamond), and engineered cells (o).

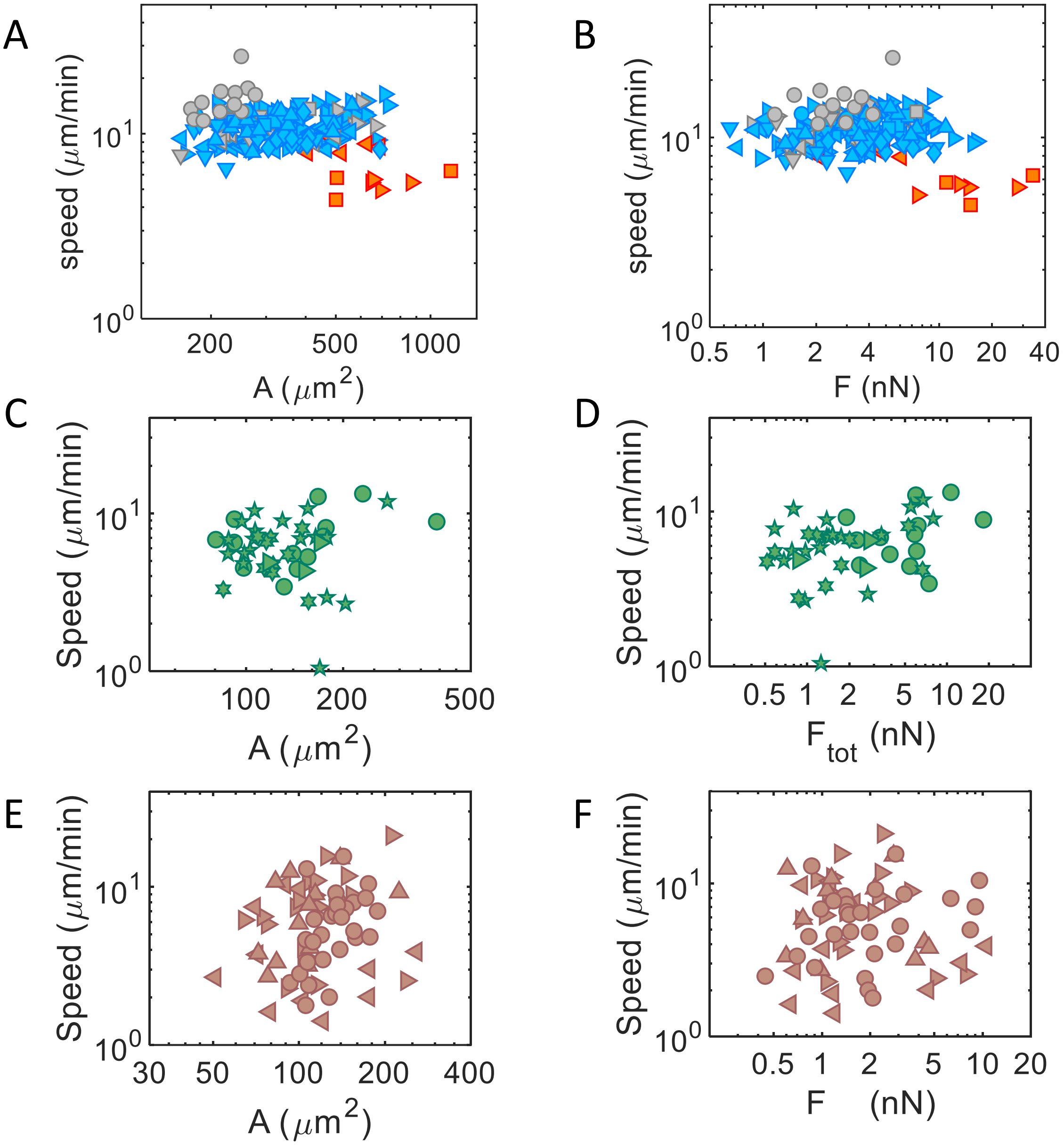

Appendix fig. S5: A&B. Speed of fan shape cells, averaged over the duration of each recording, as a function of their basal area (A) and total force (B). The color and shape of the symbols correspond to the different cell types and strains as in Appendix Fig. S4. Average speed does not vary with cell size or total force within each group of cell but varies from group to group: 10.8 (9.4/12.3) µm/min (N=161; type 1), 6.0 (5.4/8.2) µm/min (N=12; type 2), and 12.6 (10.2/14.2) µm/min (N=31; unstable cells) ($p_{type1-type2}$=2.6x10^{-7} and $p_{type1-unstable}$=9.7x10^{-4}). C&D. Speed of oscillatory cells as a function of basal area (C) and total force (D) (engineered cells: o, engineered cells limE-YFP: five-pointed stars, and engineered cells GFP-myo: six-pointed stars). E&F. Speed of amoeboid cells as a function of basal area (E) and total force (F) (AX2 LimE-GFP (>), AX2 GFP-myo (<), AX2 lifeAct-GFP (^), and engineered cells (o)).

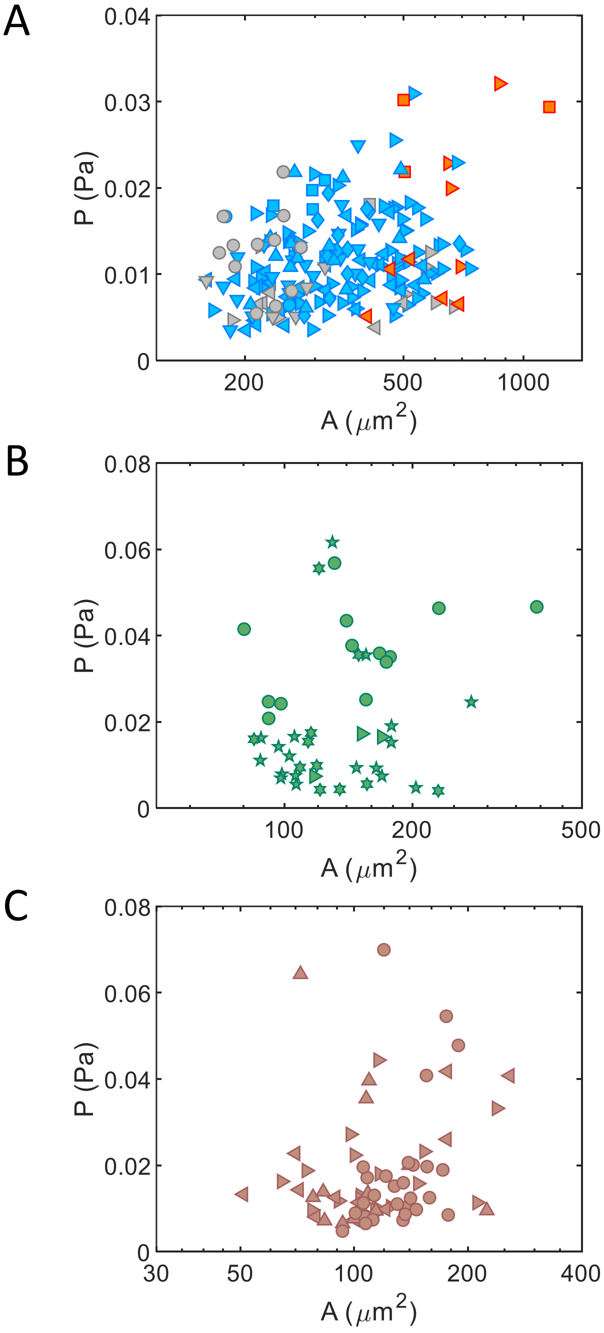

Appendix fig. S6: A-C: Pressure (total force F divided by the basal area A) as a function of the cell's basal area for fan-shaped cells (A), oscillatory cells (B) and amoeboid cells (C). The color and shape of the markers denote the different strains as specified in Appendix Fig. S4&5. The increase of F with A is not well described by a linear fit with zero y-intercept.

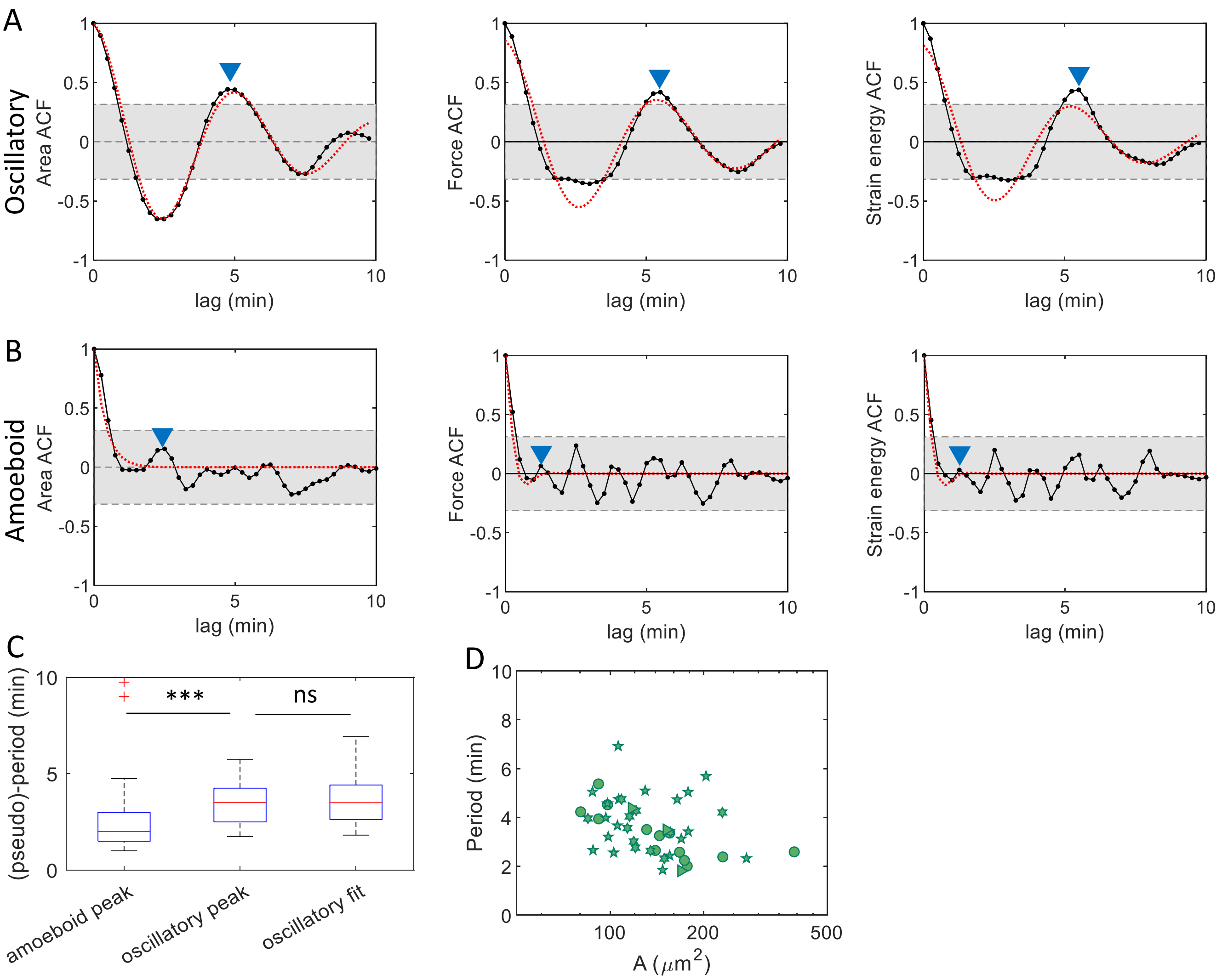

Appendix fig. S7: A-B. Auto-correlation functions (ACF) of the area, force, and strain energy for the oscillatory cell expressing LimE-GFP from Fig. 3 and for the amoeboid cell expressing LimE-GFP from Fig. 4. The ACF was computed with the 'autocorr' function in MATLAB and the 95% confidence interval correspond to the gray-shaded regions. The dashed line is a damped cosine fit to the ACF. A period of oscillation can only be extracted from this fit for the area ACF of the oscillatory cell. Also shown is the second method to find the pseudo-period of oscillation, which identifies the first positive peak of the ACF, indicated by the blue triangles in the ACF plots. The automated detection of the peak was achieved using the 'findpeaks' function in MATLAB. The comparison of the ACF of the area, the force and the strain energy indicates that the area as the mostly periodic behavior. Our measurement of the periods and pseudo-periods are thus based on the area ACF. **C.** Median, first and third quartile of the pseudo-period of amoeboid cells, using the first peak detection of the area ACF, and of oscillatory cells using the first peak detection of the area ACF and the damped cosine fit. Both methodologies gave consistent results for the median period of the oscillatory cell: 3.5 (2.6/4.4) min (Table S1, N=43). See also Appendix Table S1. D. Period of the oscillatory cells as a function of the cell area: no dependence is observed

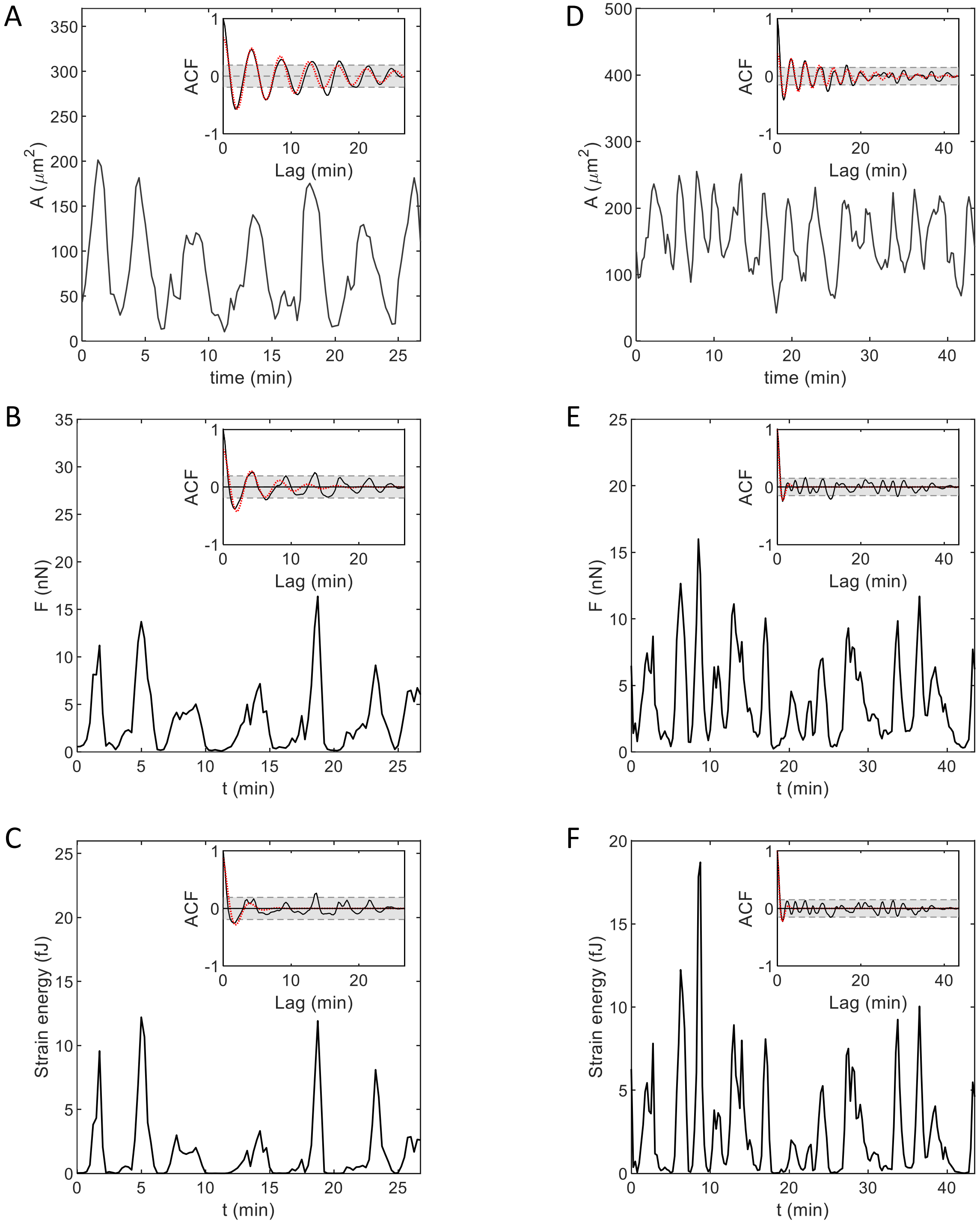

Appendix fig. S8: Two examples of the area, total force, and strain energy of oscillatory cells over an extended period of time. The insets show the corresponding ACF. The area is most clearly periodic and its ACF is used to determine the period of oscillation, whereas the force and strain energy have a less periodic behavior as shown by their ACF.

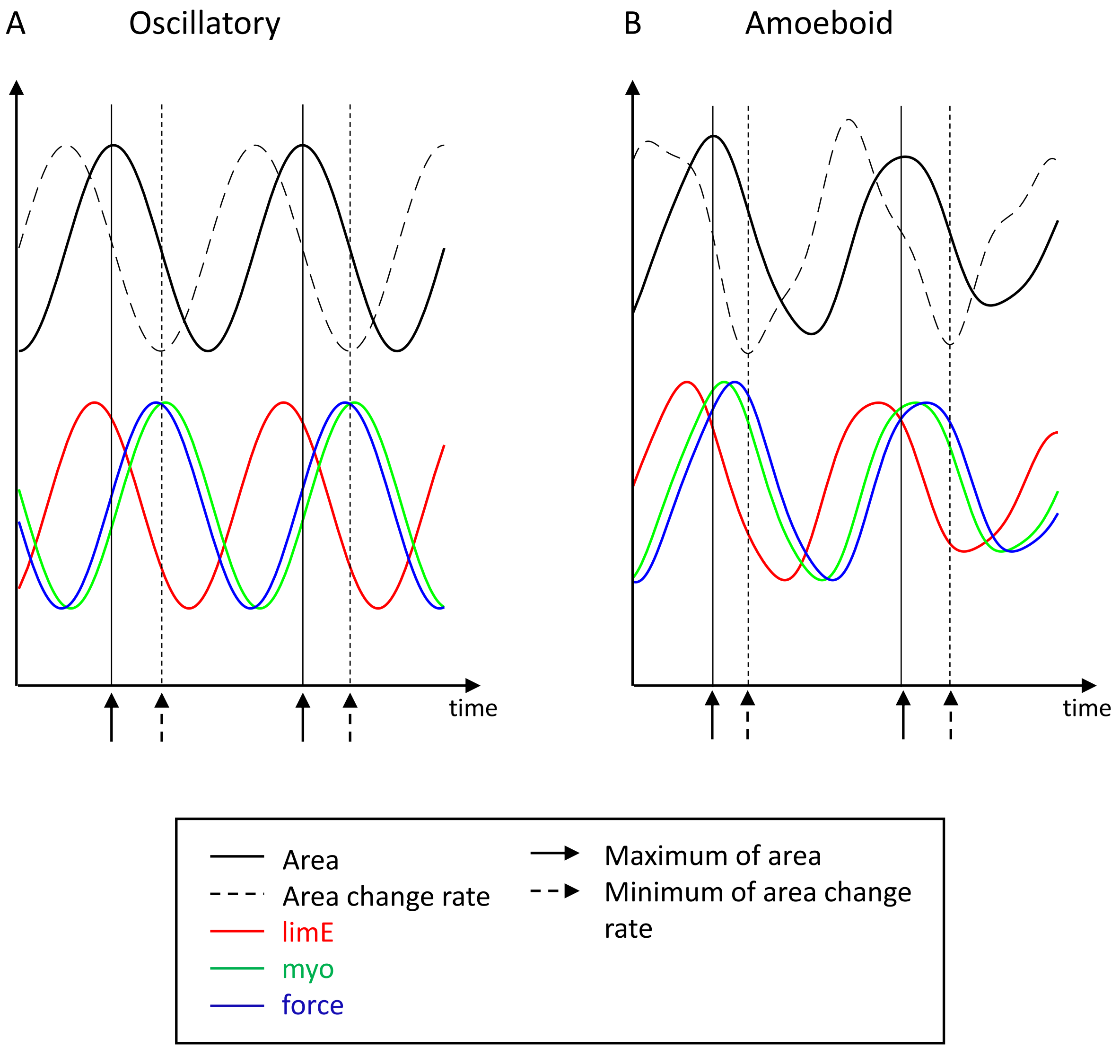

Appendix fig. S9: Summary of the temporal evolution of the area, the force and the cell-averaged limE and myosin intensity obtain from the temporal cross-correlations for the oscillatory cells (A) and for the amoeboid cells (B).

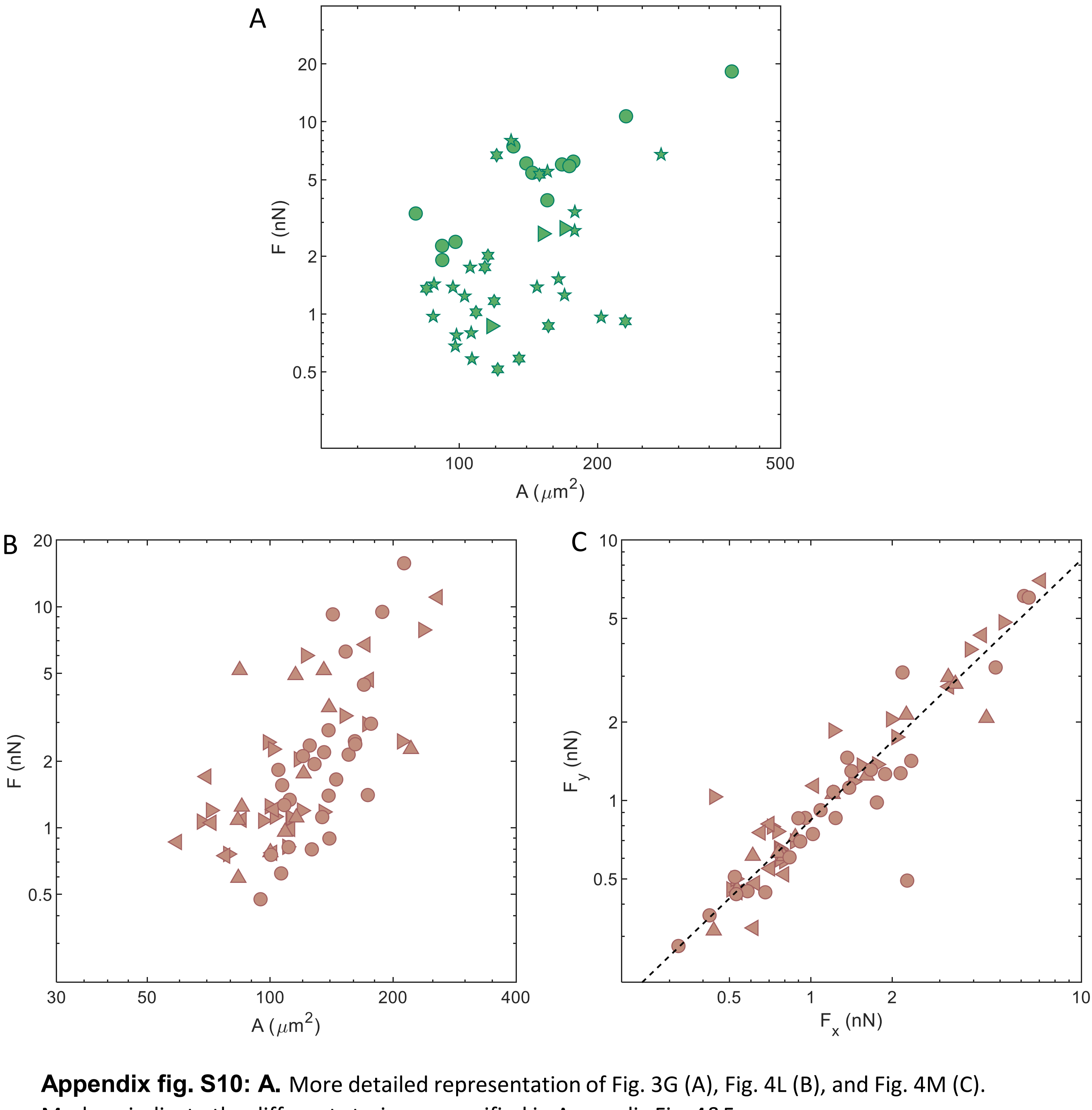

Appendix fig. S10: A. More detailed representation of Fig. 3G (A), Fig. 4L (B), and Fig. 4M (C). Markers indicate the different strains as specified in Appendix Fig. 4&5.

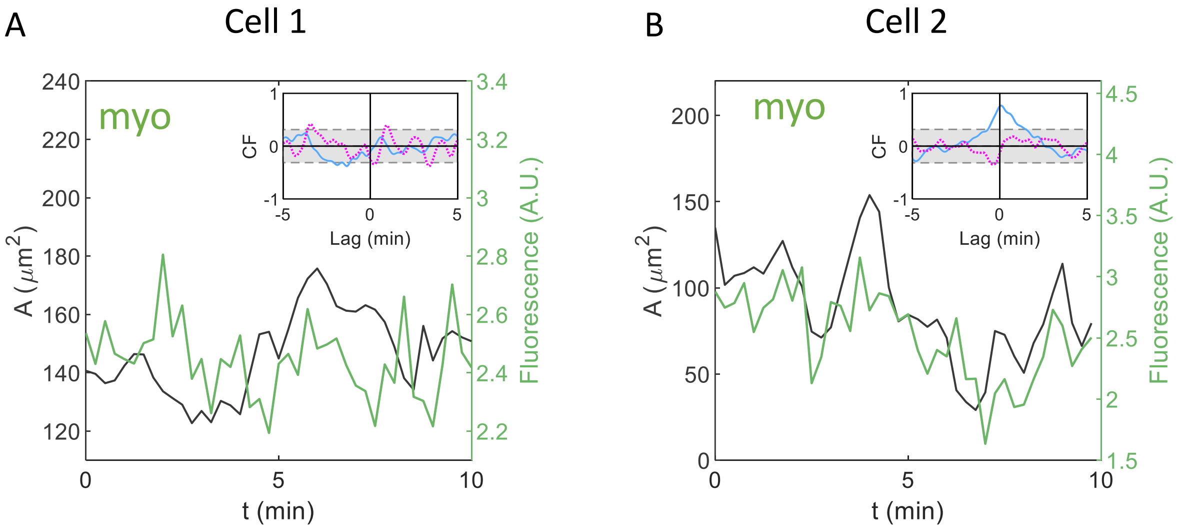

Appendix fig. S11: Basal area (black line) and average fluorescence (green line) as a function of time corresponding to two amoeboid cells expressing GFP-myo. Cell displayed in (A) is the same as in Fig. 4. Inset: Temporal cross-correlation function (CF) of the area and the fluorescent signal (blue) and of the area change rate and the fluorescent signal (magenta). The correlation within the shaded region is below the 95% confidence interval. A significant correlation is found for the cell in panel (B), but not for the cell in panel (A), illustrating the variety observed in the data.

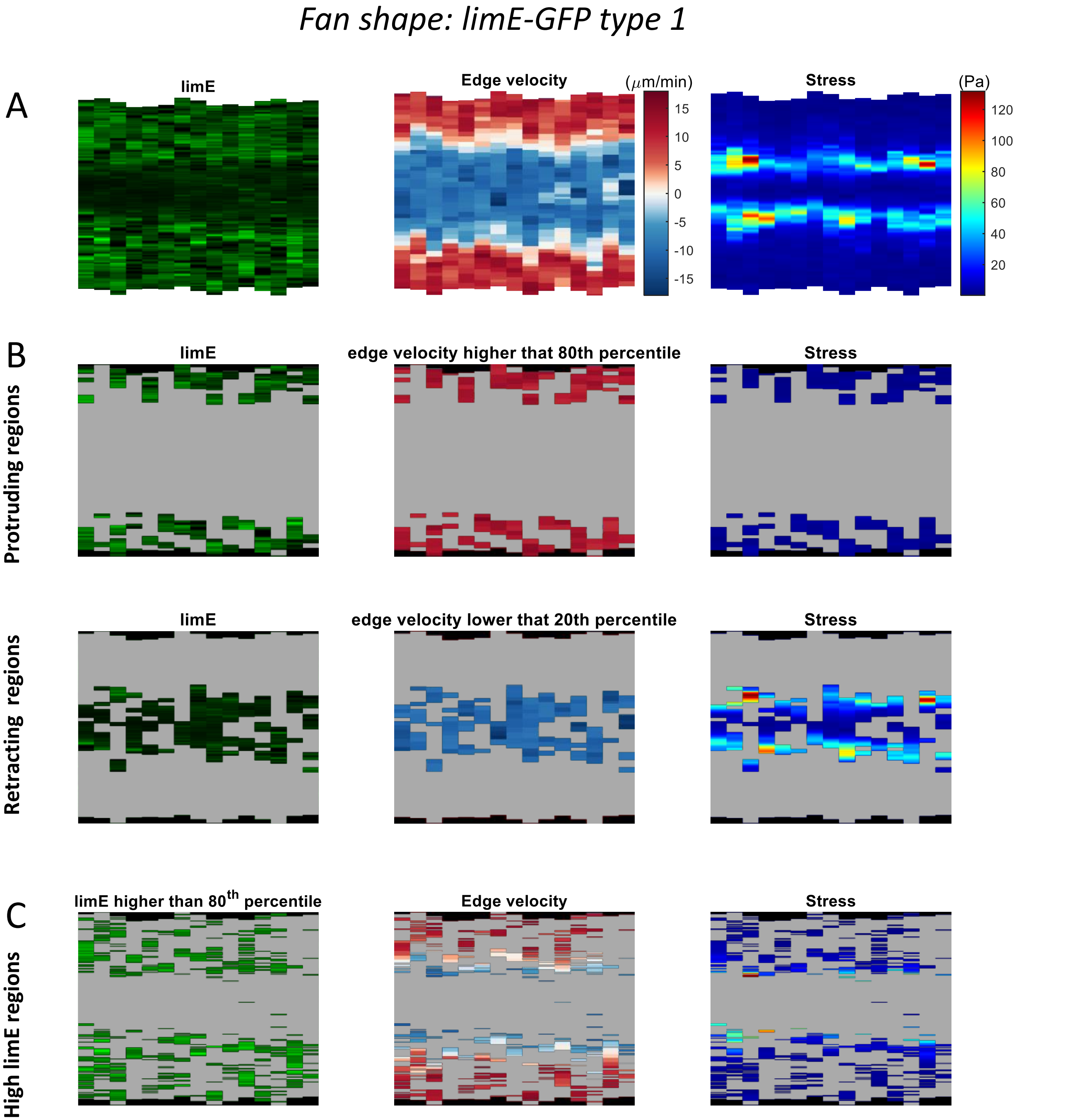

Appendix fig. S12: A. Cell membrane kymographs of the LimE intensity, edge velocity, and stress of a type 1 fan shape cell expressing LimE-GFP. Here, and elsewhere, positive normal velocities, corresponding to protrusions of the membrane, are shown in red and negative normal velocities, indicating retractions, are shown in blue. Note that these moving cells contain regions of vanishing normal velocity, which correspond to the sides of the cell. **B&C.** Same kymographs as in (A) with masks to only display pixels in the protruding and retracting regions (B) or in the region of high fluorescence (C).

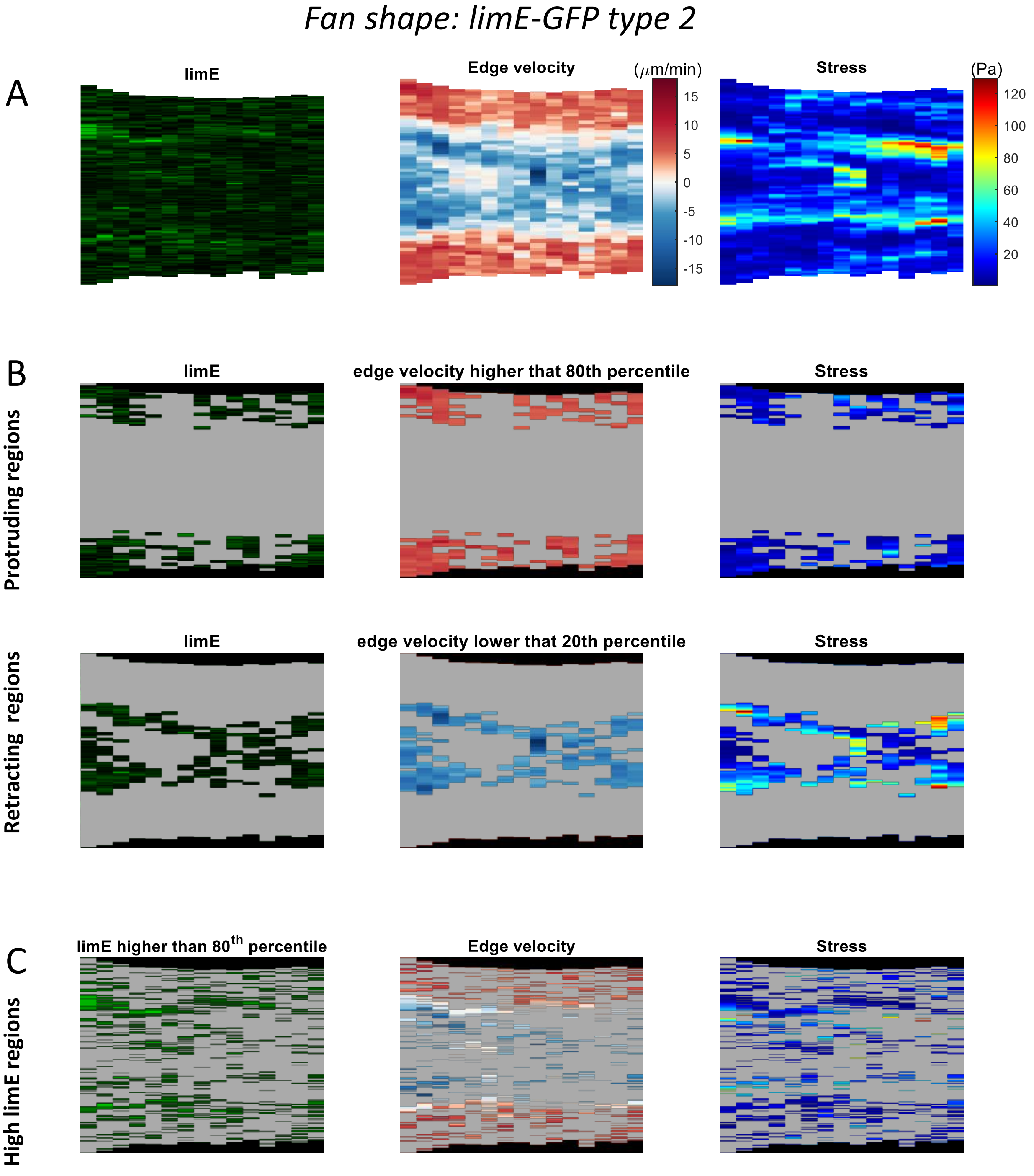

Appendix fig. S13: A. Cell membrane kymographs of the LimE intensity, edge velocity, and stress of a type 2 fan shape cell expressing LimE-GFP. **B and C.** Same kymographs as in (A) with masks to only display pixels in the protruding and retracting regions (B) or in the region of high fluorescence (C).

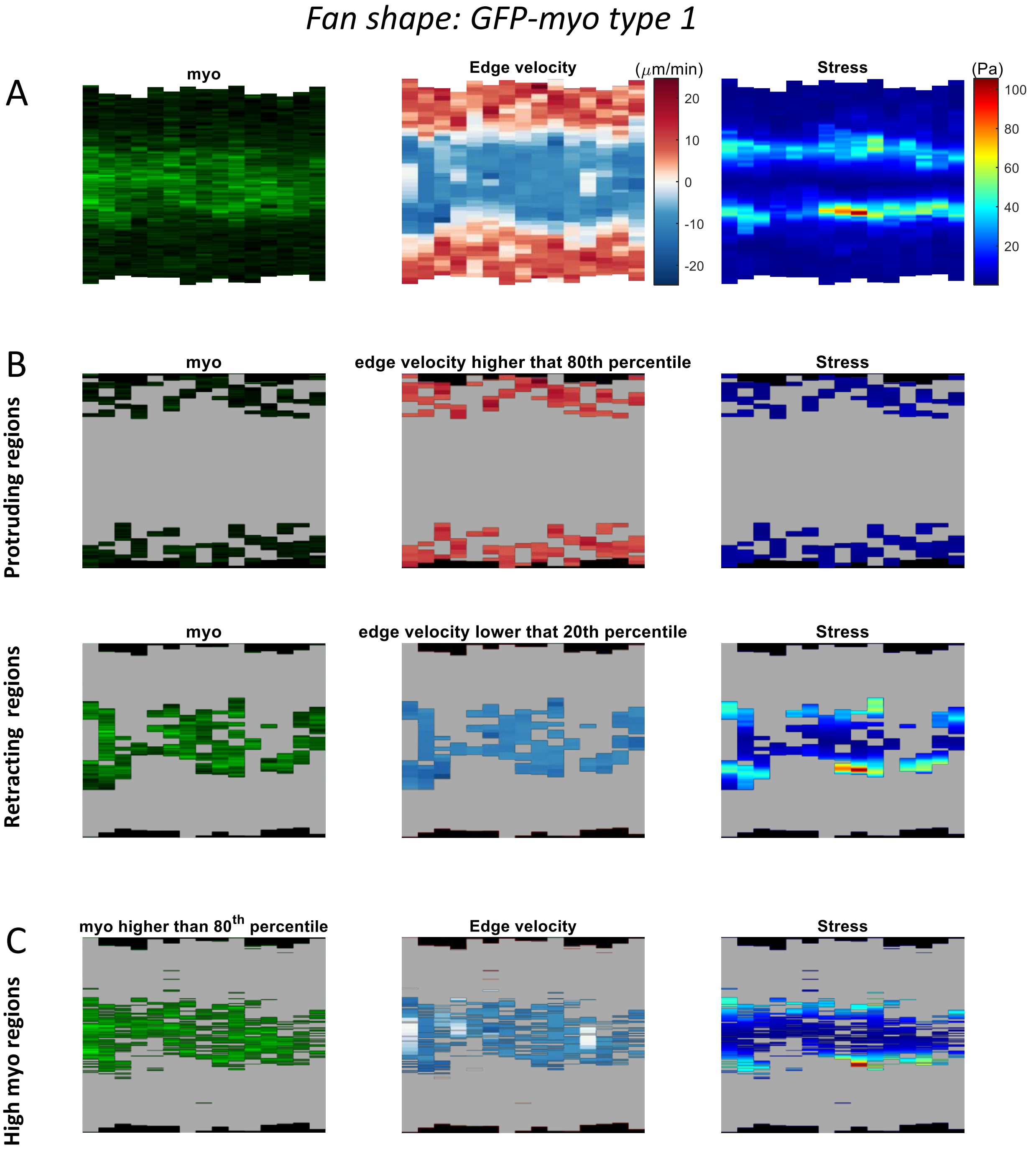

Appendix fig. S14: A. Cell membrane kymographs of the myosinII intensity, edge velocity, and stress cell membrane of a type 1 fan shape cell expressing GFP-myo. **B&C.** Same kymographs as in (A) with masks to only display pixels in the protruding and retracting regions (B) or in the region of high fluorescence (C).

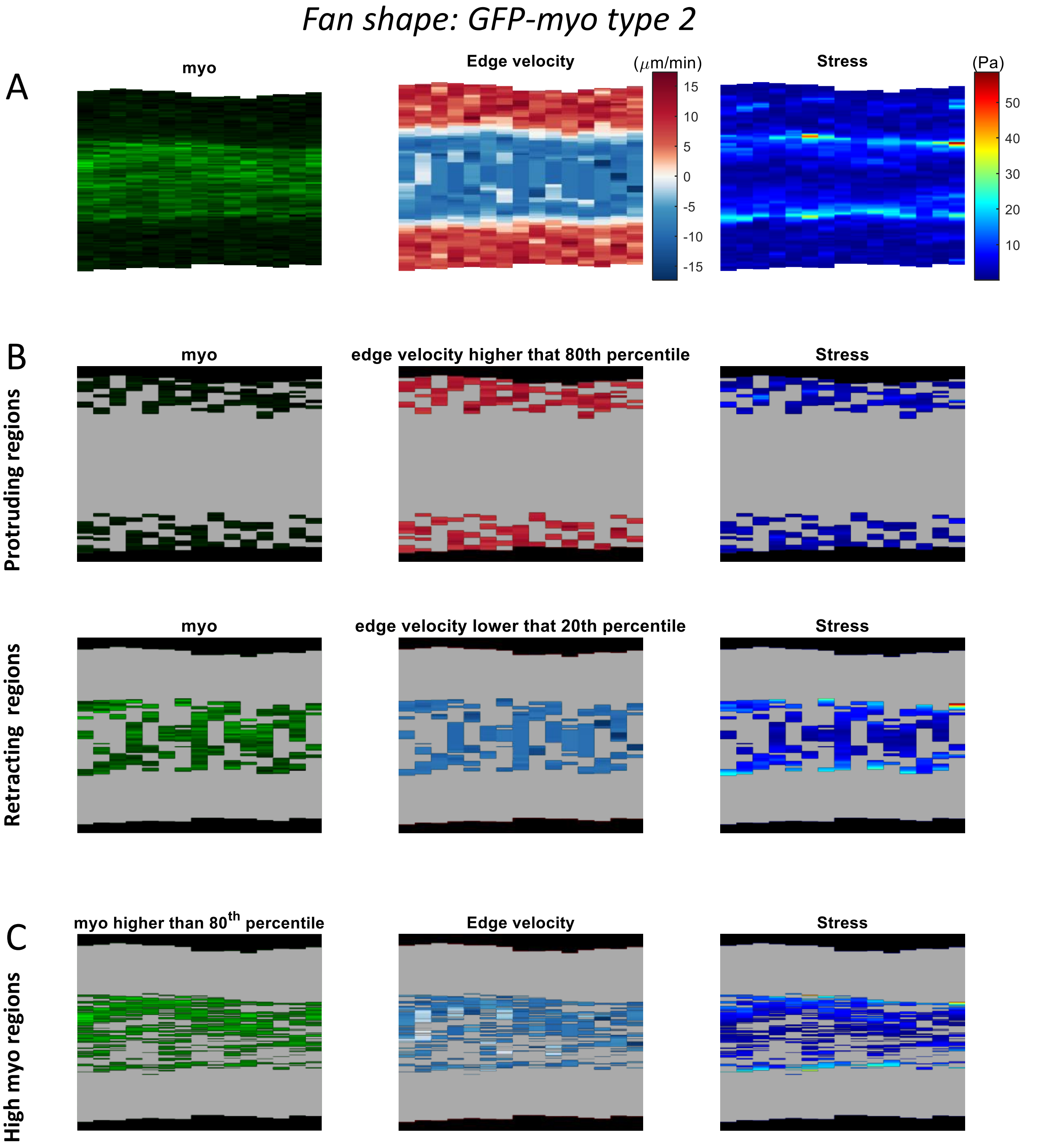

Appendix fig. S15: A. Cell membrane kymographs of the myosinII intensity, edge velocity, and stress of a type 2 fan shape cell expressing GFP-myo. **B and C.** Same kymographs as in (A) with masks to only display pixels in the protruding and retracting regions (B) or in the region of high fluorescence (C).

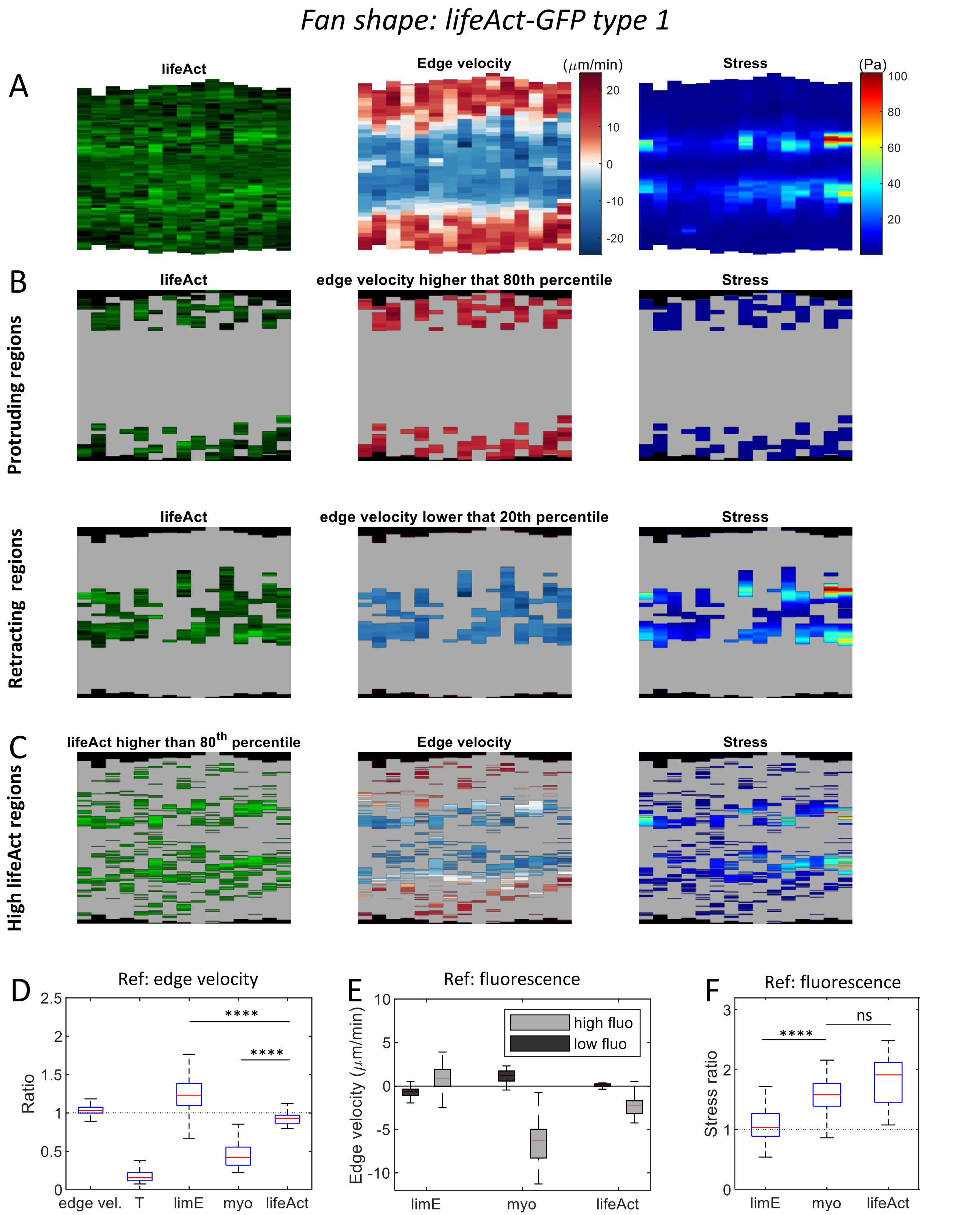

Fan shape: lifeAct-GFP type 1
A
lifeAct
Edge velocity
(μm/min)
20
10
0
-10
-20
Stress
(Pa)
100
80
60
40
20
B
Protruding regions
lifeAct
edge velocity higher that 80th percentile
Stress
Retracting regions
lifeAct
edge velocity lower that 20th percentile
Stress
C
High lifeAct regions
lifeAct higher than 80th percentile
Edge velocity
Stress
D
Ref: edge velocity
Ratio
edge vel.
T
limE
myo
lifeAct
****
****
E
Ref: fluorescence
Edge velocity (μm/min)
high fluo
low fluo
limE
myo
lifeAct
F
Ref: fluorescence
Stress ratio
****
ns
limE
myo
lifeAct

Appendix fig. S16: A. Cell membrane kymographs of the lifeAct intensity, edge velocity, and stress of a type 2 fan shape cell expressing lifeAct -GFP. **B and C.** Same kymographs as in A with masks to only display pixels in the protruding and retracting regions (B) or in the region of high fluorescence (C). **D.** Ratio of the average edge velocity (N=166), stress (N=166) and fluorescence intensity in the protruding regions (N=85/39/15) compared to the retracting regions. **E.** Average edge velocity in the regions of low and high fluorescence for amoeboid cells expressing LimE-GFP (N=85), GFP-myoII (N=39) and lifeAct-GFP (N=15). **F.** Ratio of the average stress in the regions of high fluorescence compared to the regions of low fluorescence, for LimE-GFP, GFP-myoII and lifeAct-GFP.

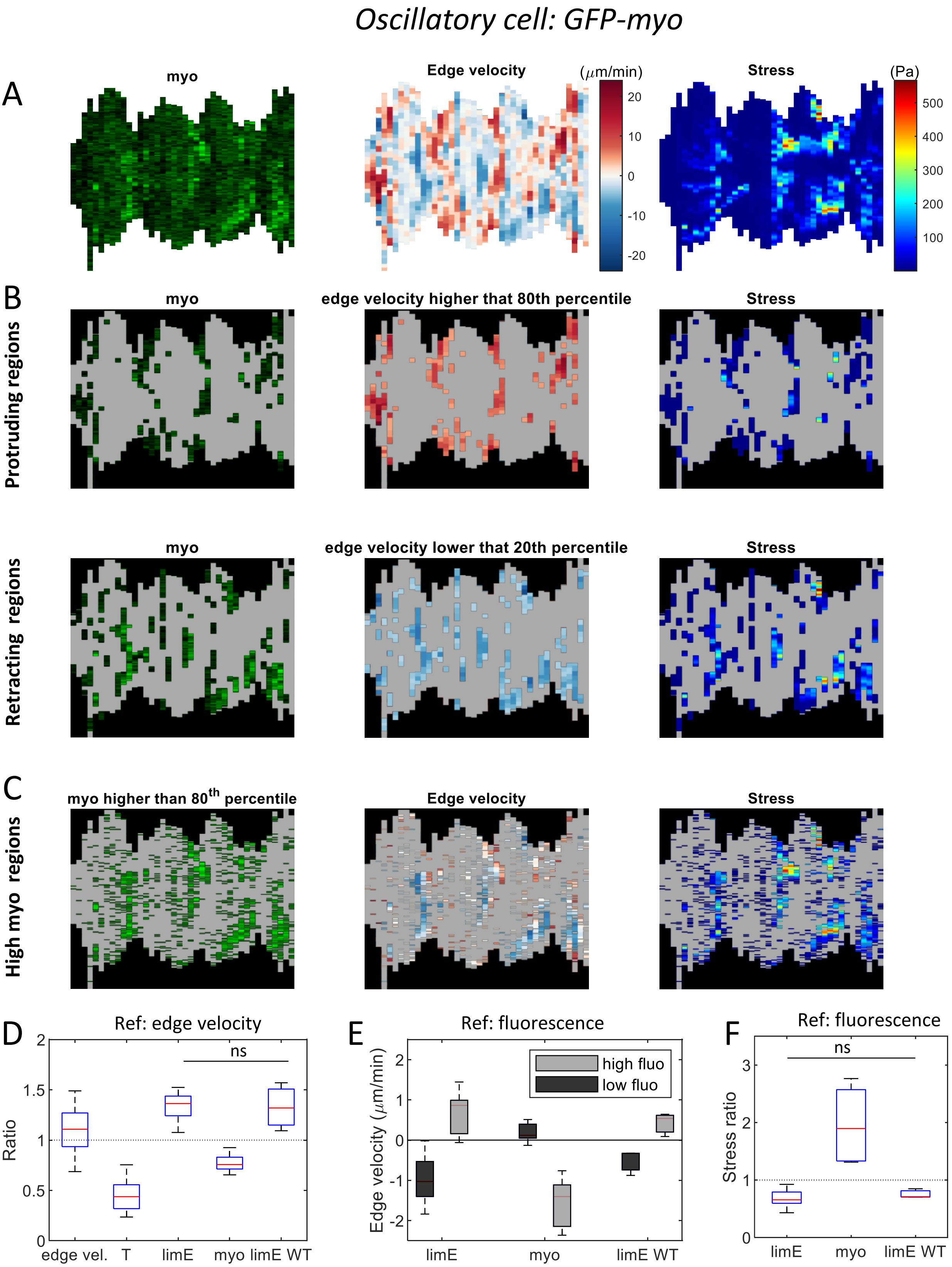

Oscillatory cell: GFP-myo
A
myo
Edge velocity
(μm/min)
20
10
0
-10
-20
Stress
(Pa)
500
400
300
200
100
B
Protruding regions
myo
edge velocity higher that 80th percentile
Stress
Retracting regions
myo
edge velocity lower that 20th percentile
Stress
C
High myo regions
myo higher than 80th percentile
Edge velocity
Stress
D
Ref: edge velocity
ns
Ratio
edge vel.
T
limE
myo
limE WT
E
Ref: fluorescence
high fluo
low fluo
Edge velocity (μm/min)
limE
myo
limE WT
F
Ref: fluorescence
ns
Stress ratio
limE
myo
limE WT

Appendix fig. S17: A. Kymographs along an engineered oscillatory cell's outline for the GFP-myoII intensity, edge velocity and stress corresponding to the cell expressing GFP-myosinII in Fig. 4A. **B&C.** Kymographs as in (A) with masks applied to only display pixels in the protruding and retracting regions (B) or in the region of high fluorescence (C). **D.** Ratio of the average edge velocity (N=37), stress (N=37) and fluorescence in the protruding regions compared to the retracting regions. **E.** Ratio of the average stress in the regions of high fluorescence compared to the regions of low fluorescence, for LimE-YFP (N=17) and GFP-myoII (N=11) of engineered cells and LimE-GFP of wild type cells (N=3). Few wild cells were observed in the oscillatory mode while performing the fan shape cells experiment. **F.** Ratio of the average stress in the regions of high fluorescence compared to the regions of low fluorescence, for engineered oscillatory cells expressing LimE-GFP and expressing GFP-myoII and for wild type oscillatory cells expressing LimE-GFP.

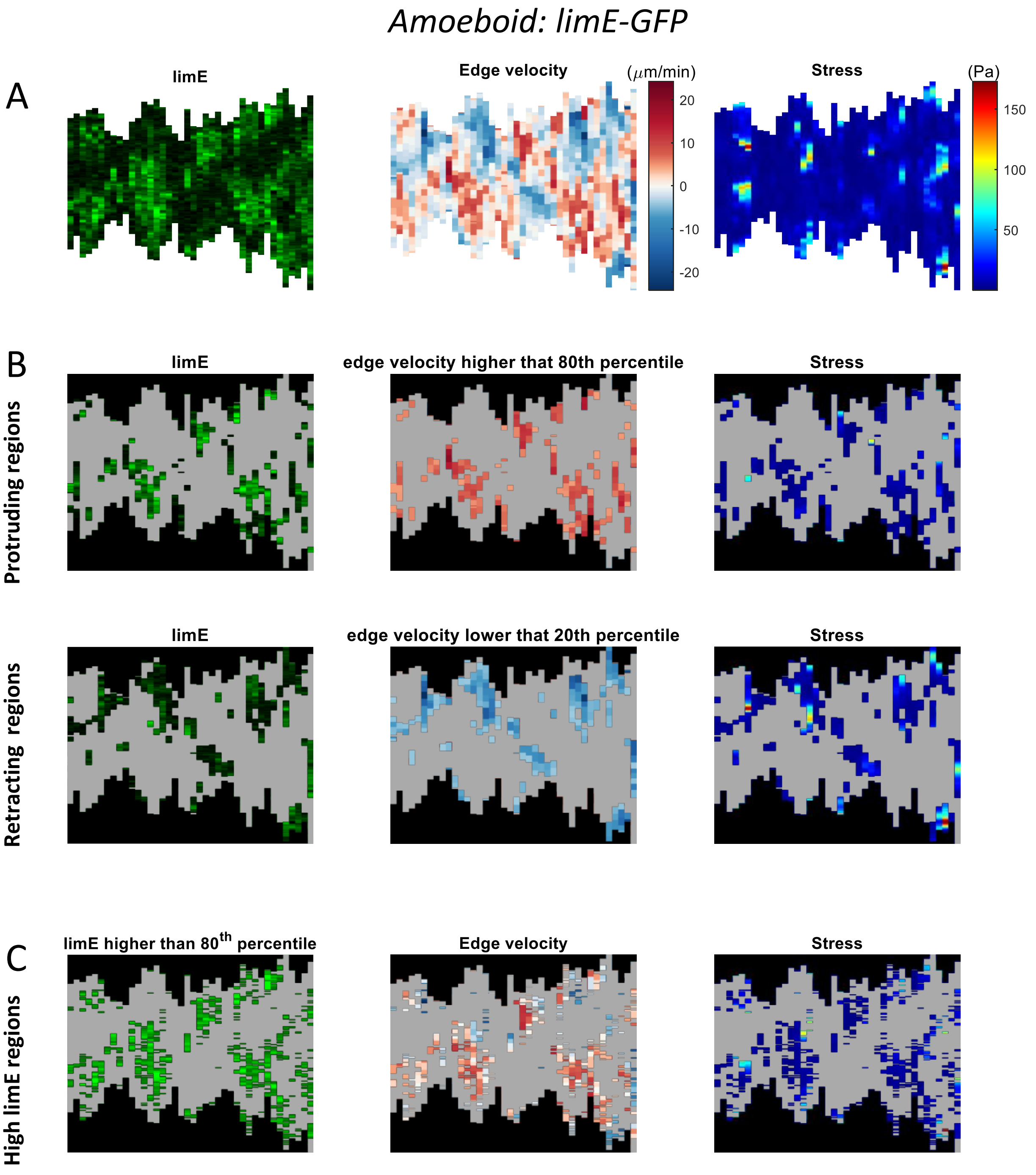

Appendix fig. S18: A. Kymograph along an amoeboid cell's outline for the LimE-GFP intensity, edge velocity and stress of the first cell in Fig. 2A. **B&C.** Same kymographs with masks to only display pixels in the protruding and retracting regions (B) or in the region of high fluorescence (C).

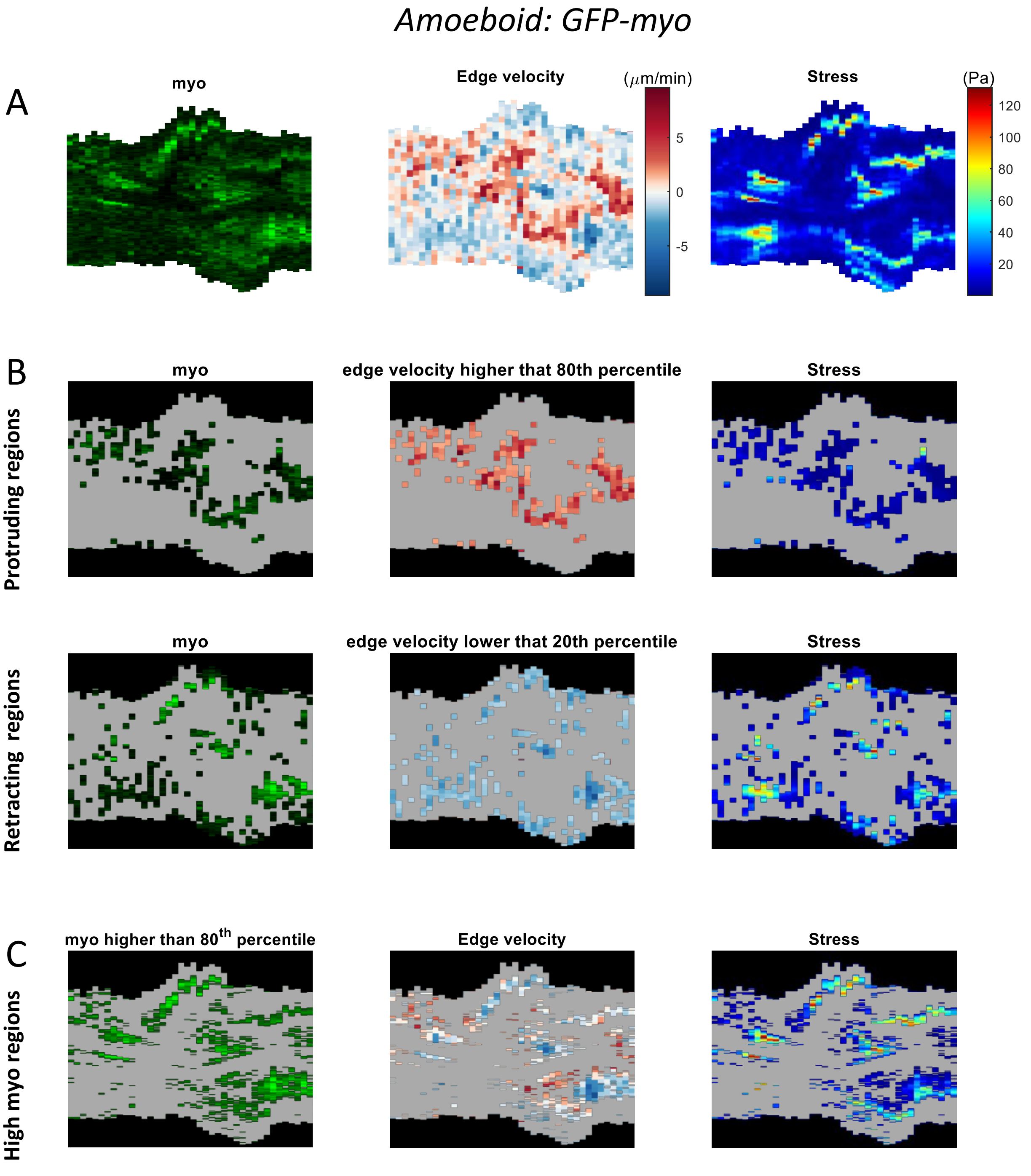

Appendix fig. S19: A. Kymograph along an amoeboid cell's outline for the GFP-myo intensity, edge velocity and stress of the second cell in Fig. 2A. **B and C.** Same kymographs with masks to only display pixels in the protruding and retracting regions (B) or in the region of high fluorescence (C).

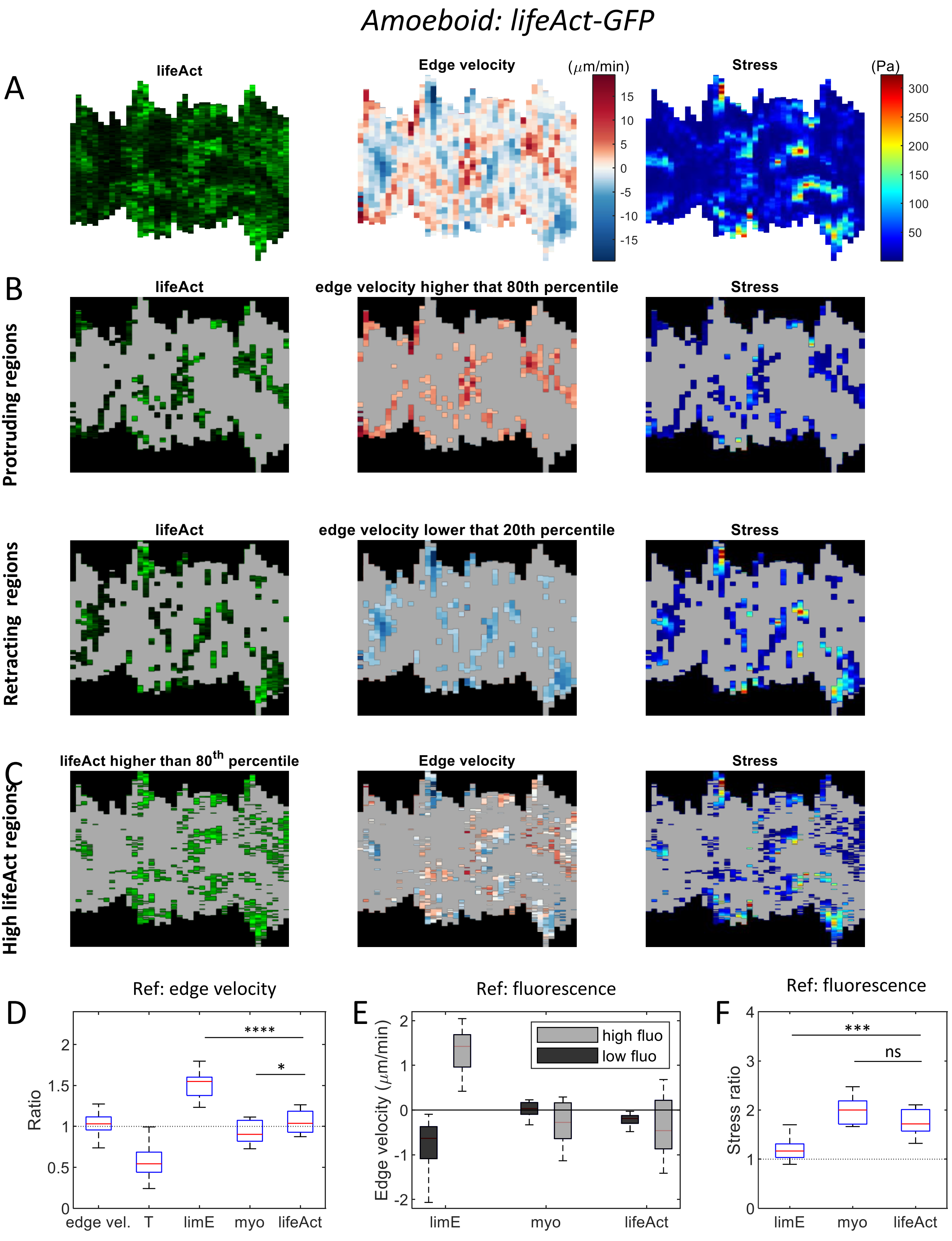

Amoeboid: lifeAct-GFP
A
lifeAct
Edge velocity
(μm/min)
15
10
5
0
-5
-10
-15
Stress
(Pa)
300
250
200
150
100
50
B
Protruding regions
lifeAct
edge velocity higher that 80th percentile
Stress
Retracting regions
lifeAct
edge velocity lower that 20th percentile
Stress
C
High lifeAct regions
lifeAct higher than 80th percentile
Edge velocity
Stress
D
Ref: edge velocity
Ratio
****
*
edge vel.
T
limE
myo
lifeAct
E
Ref: fluorescence
Edge velocity (μm/min)
high fluo
low fluo
limE
myo
lifeAct
F
Ref: fluorescence
Stress ratio
***
ns
limE
myo
lifeAct

Appendix fig. S20: A. Kymograph along the outline of an amoeboid cell expressing lifeAct-GFP for the GFP intensity, edge velocity and stress. **B&C.** Same kymographs with masks to only display pixels in the protruding and retracting regions (B) or in the region of high fluorescence (C). **D.** Ratio of the average edge velocity (n=56), stress (n=56) and fluorescence intensity in the protruding regions compared to the retracting regions. **E.** Average edge velocity in the regions of low and high fluorescence for amoeboid cells expressing LimE-GFP (n=17), GFP-myoII (n=10) and lifeAct-GFP (n=11). **F.** Ratio of the average stress in the regions of high fluorescence compared to the regions of low fluorescence, for LimE-GFP, GFP-myoII and lifeAct-GFP.

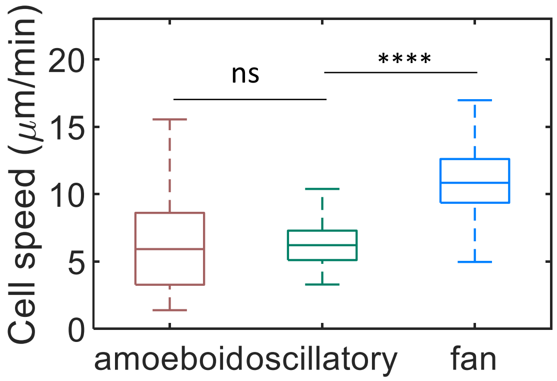

Appendix fig. S21: Translational cell speed computed from the cell center of mass for amoeboid (N=56), fan-shaped (N=166), and oscillatory cells (N=37). Fan-shaped cells exhibited a much higher translational cell speed than amoeboid cells (10.8 (9.4/12.6) μm/min vs 5.9 (3.3/8.6) μm/min).

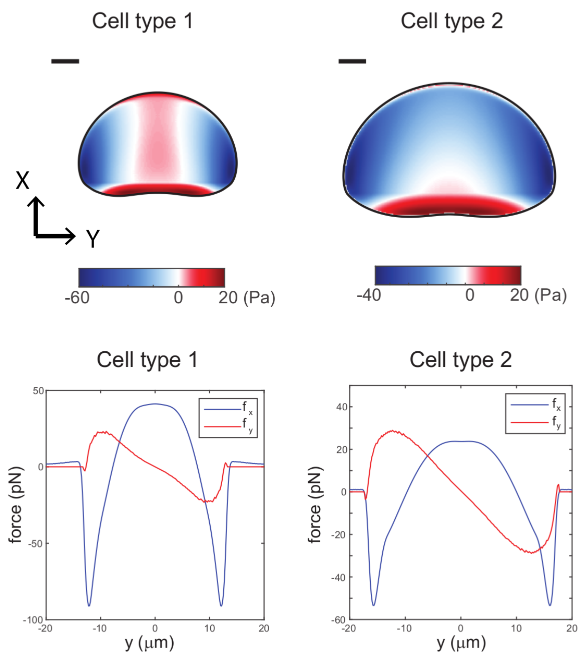

Appendix Fig. S22. Upper panels: the stress in the direction of motion (T_x) for a type 1 (left) and type 2 (right) cell in the simulations. The cells are moving upwards. Lower panels: The x and y component of the force, f_x and f_y, integrated along x (direction of motion), as a function of y for the simulated cell type 1 and type 2. Scale bar in panels: 5 μm.

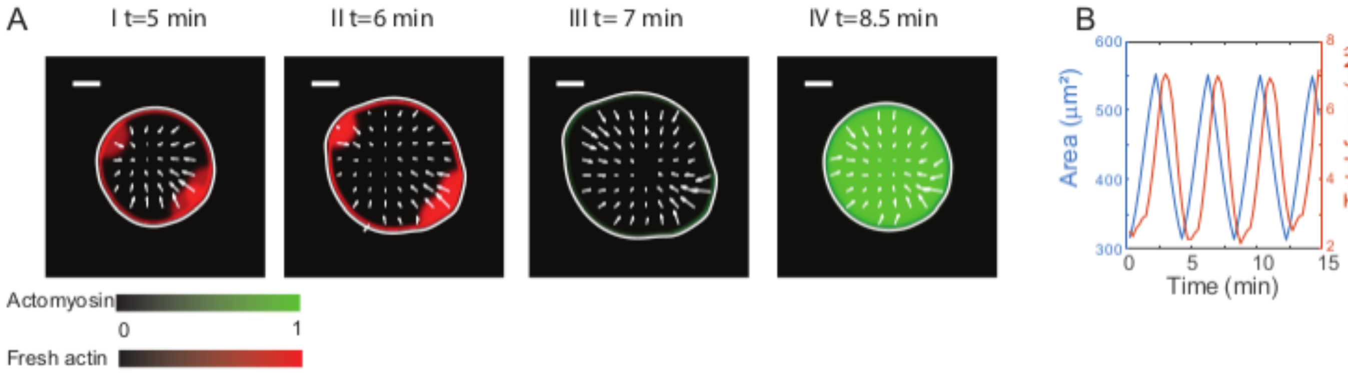

Appendix Fig. S23. (A) Simulated traction force patterns for oscillatory cells for distributions of actin and myosin that are not spatially homogeneous but are synchronized. The area and total force clearly display oscillatory behavior (B).

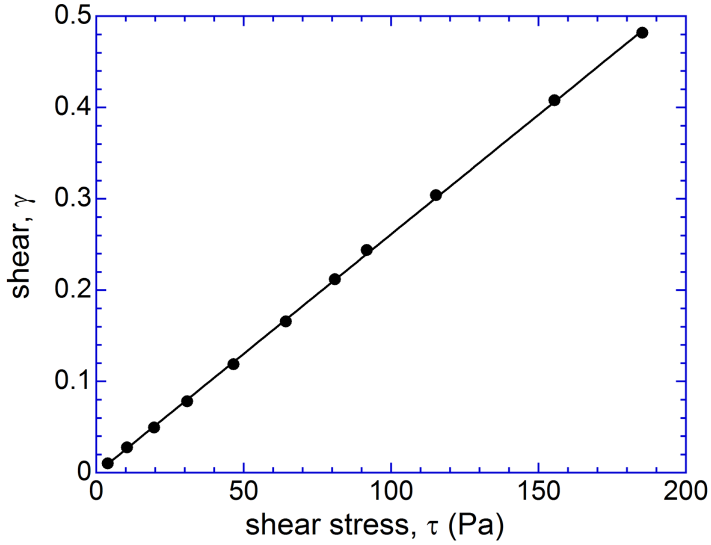

Appendix Fig. S24. Average shear *γ* of a ~1 mm thick gel layer as a function of stress *τ* (black dots) and the corresponding linear fit (uncertainty 0.5%;R^2=0.9999; slope $2.62 \cdot 10^{-3}$ Pa^{-1}. The stress was applied using a centrifugal rheometer (Ronan, E., “Centrifugal Rheometry and Rapid Stimulation of Dinoflagellate Bioluminescence in a Microfluidic Device“, PhD Dissertation, UC San Diego, 2018)). The Young’s modulus was calculated using $E=2(1+ \mu)\tau/\gamma$, which, assuming a Poisson ratio of approximately μ~0.5 for soft silicone gels, resulted for this gel in E~1.15 kPa. The elastic moduli of the silicone gel samples prepared on different days varied within ~20%, and we therefore used a value of E=1kPa in our study.

Appendix table S1:

(Pseudo-)period of oscillations determined using different auto-correlation functions for different migration modes		
**ACF**	**Amoeboid: pseudo-period**<br><br>Median (Q1/Q3) (min)	**Oscillatory: period**<br><br>Median (Q1/Q3) (min)
**Area (fit)**	N/A	3.5 (2.6/4.4) N=45
**Area (first peak)**	2.0 (1.5/3.8) N=69	3.5 (2.5/4.3) N=43
**F_{tot} (first peak)**	2.3 (1.5/3.5) N=69	3.0 (2.3/4.3) N=45
**Strain energy (first peak)**	2.3 (1.5/3.5) N=69	3.0 (2.1/4.2) N=44

Appendix table S2: data from fig. 3E&F and fig. 4D&E

Time shifts in cross correlation functions for different migration modes				
	**Amoeboid**		**Oscillatory**	
	**Area**<br><br>Median (Q1/Q3) (s)	**Area change rate**<br><br>Median (Q1/Q3) (s)	**Area**<br><br>Median (Q1/Q3) (s)	**Area change rate**<br><br>Median (Q1/Q3) (s)
**Force**	Correlated<br><br>11.3 (3.8/15)	Anti-correlated<br><br>-11.2 (-15.0/-9.4)	Correlated<br><br>33.8 (22.5/42.2)	Anti-correlated<br><br>-3.7 (-11.2/3.8)
**LimE**	Correlated<br><br>-11.2 (-15.0/-7.5)	Correlated<br><br>11.3 (7.5/11.3)	Correlated<br><br>-11.2 (-19.6/-10.3)	Correlated<br><br>16.9 (7.5/30.0)
**Myosin**	Correlated<br><br>7.5 (1.9/12.2)	Anti-correlated<br><br>-26.3 (-30.0/-14.1)	Correlated<br><br>52.5 (41.3/62.8)	Anti-correlated<br><br>3.8 (0/7.5) s

Appendix table S3:

Cell speed, edge velocity, and relevant ratios for the three different migration modes

		Amoeboid<br>Median (Q1/Q3) (s)	Fan-shape<br>Median (Q1/Q3) (s)	Oscillatory<br>Median (Q1/Q3) (s)
**Cell speed in µm/min**		5.9 (3.3/8.6) N=56	10.8 (9.4/12.6) N=166	6.2 (5.1/7.3) N=37
		**Reference: edge velocity**		
**Edge velocity in µm/min**	Retraction	-6.5 (-9.4/-4.1) N=56	-11.7 (-13.2/-10.3) N=166	-8.0 (-10.2/-6.9) N=37
	Protrusion	6.7 (4.7/9.3) N=56	12.3 (10.6/14.3) N=166	8.5 (8.0/10.0) N=37
**Ratio**	edge velocity	1.03 (0.96/1.12) N=56	1.03 (1.00/1.07) N=166	1.10 (0.94/1.27) N=37
	Stress	0.54 (0.44/0.69) N=56	0.16 (0.12/0.22) N=166	0.44 (0.32/0.56) N=37
	LimE	1.55 (1.38/1.60) N=17	1.23 (1.10/1.38) N=85	1.36 (1.24/1.44) N=16
	myosin	0.90 (0.82/1.07) N=10	0.42 (0.32/0.55) N=39	0.76 (0.71/0.83) N=10
	lifeAct	1.04 (0.93/1.19) N=11	0.93 (0.86/0.97) N=15	
	LimE (WT osci)			1.32 (1.15/1.51) N=3
		**Reference: fluorescence**		
**Edge velocity in µm/min**	Low LimE	-0.6 (-1.1/0.4) N=17	-0.59 (-1.09/-0.35) N=85	-1.03 (-1.40/-0.53) N=16
	High LimE	1.4 (1.0/1.7) N=17	0.90 (-0.05/1.91) N=85	0.86 (0.16/0.99) N=16
	Low myo	0.0 (-0.1/0.2) N=10	1.23 (0.58/1.74) N=39	0.12 (0.05/0.40) N=10
	High myo	-0.3 (-0.6/0.2) N=10	-6.24 (-8.27/-4.97) N=39	-1.40 (-2.15/-1.11) N=10
	Low lifeAct	-0.2 (-0.3/-0.1) N=11	0.12 (-0.06/0.25) N=15	
	High lifeAct	-0.5 (-0.9/0.2) N=11	-2.20 (-3.17/-1.69) N=15	
	Low LimE (WT osci)			-0.33 (-0.74/-0.33) N=3
	High LimE (WT osci)			0.54 (0.20/0.62) N=3
**Stress ratio**	LimE	1.17 (1.03/1.31) N=17	1.04 (0.89/1.27) N=85	0.66 (0.60/0.79) N=16
	myosin	2.00 (1.71/2.19) N=10	1.58 (1.39/1.77) N=39	1.90 (1.33/2.57) N=10
	lifeAct	1.72 (1.57/2.01) N=11	1.91 (1.46/2.12) N=15	
	LimE WT (osci)			0.71 (0.70/0.81) N=3
