# Supplementary material for: Coupling traction force patterns and actomyosin wave dynamics reveals mechanics of cell motion
Source: Mol Syst Biol. 2021 Dec 13;17(12):e10505. doi: 10.15252/msb.202110505 (PMC8666840; doi:10.15252/msb.202110505)
Supplement: Supplementary file 2 — Expanded View Figures PDF [file MSB-17-e10505-s013.pdf]

Expanded View Figures

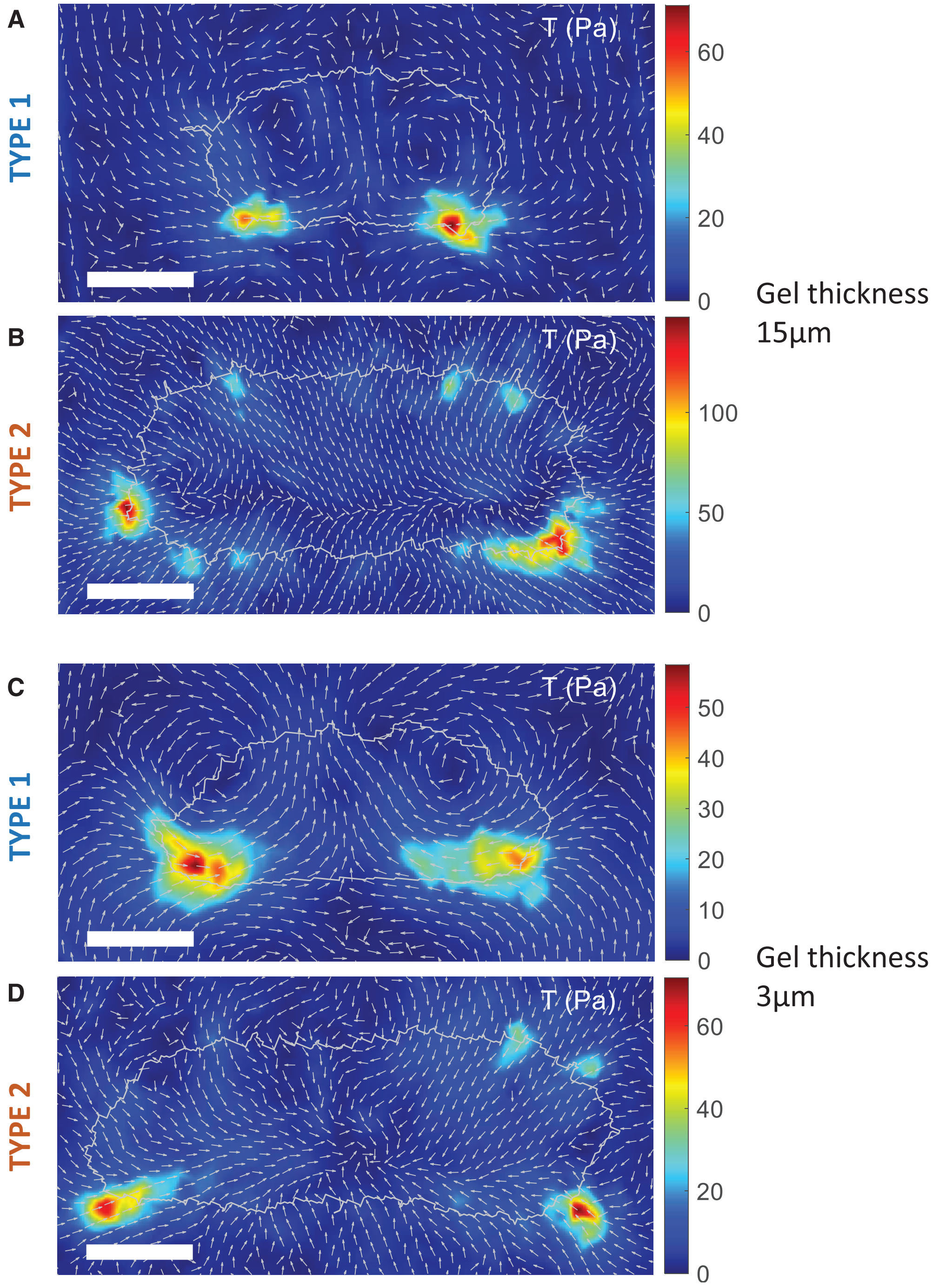

Figure EV1. Detailed traction force maps for different gel thickness.

A, B Detailed TFMs of type 1 and type 2 fan-shaped cells of Fig 2 using a gel with a thickness of 15 μm.

C, D As (A) and (B) but now for a gel with a thickness of 3 μm. Markers only indicate the direction of the stress, regardless of the magnitude, so that the direction is visible even for low stresses.

Data information: The arrows indicate the traction force. All scale bars in the figure: 10 μm.

 © 2021 The Authors

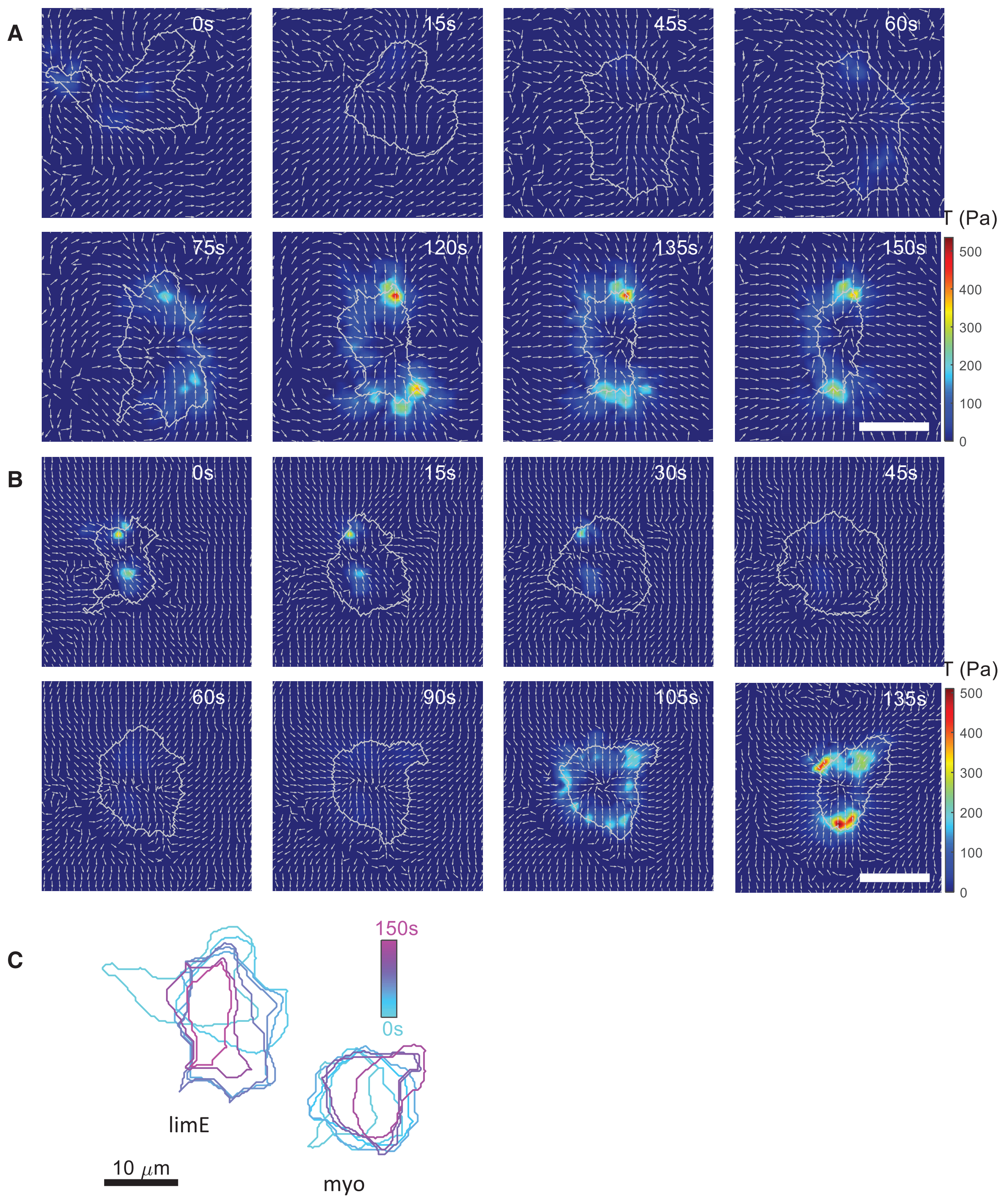

Figure EV2. Detailed traction force maps for oscillatory cells.

A Detailed traction force map for the oscillatory cell with LimE-GFP presented in Fig 3.
B Detailed traction force map of the second cell of Fig 3, expressing GFP-myo. Arrows indicate the stress direction, regardless of the magnitude.
C Outlines of the cells presented in Fig 3, at the corresponding times.

Data information: The arrows indicate the traction force. All scale bars in the figure: 10 μm.

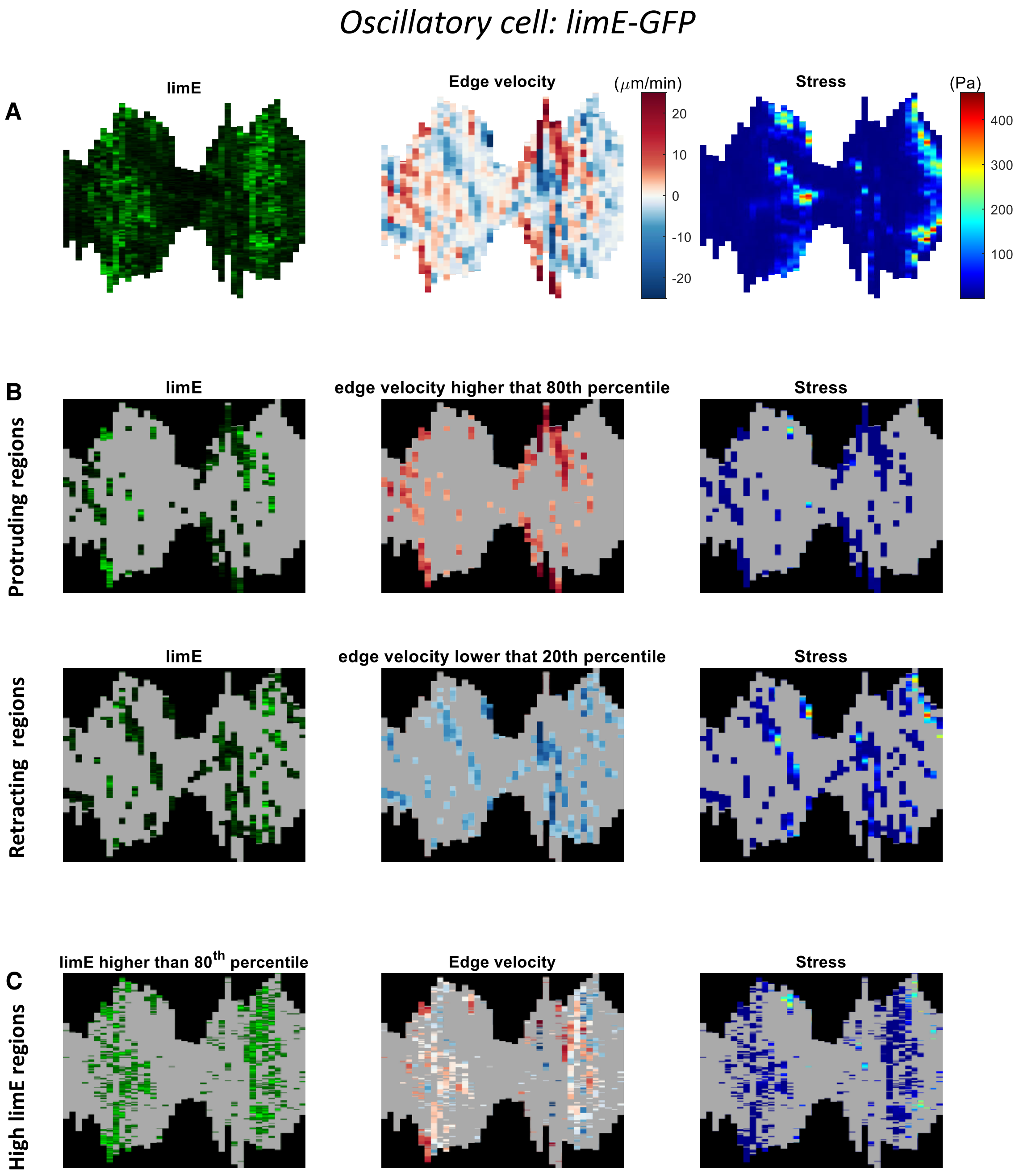

Figure EV3. Kymographs of an oscillatory cell expressing LimE.

A Kymographs along an engineered oscillatory cell's outline for the LimE-GFP intensity, edge velocity, and stress corresponding to the cell expressing LimE in Fig 3A.

B, C Kymographs as in (A) with masks applied to only display pixels in the protruding and retracting regions (B) or in the region of high fluorescence (C).

 © 2021 The Authors

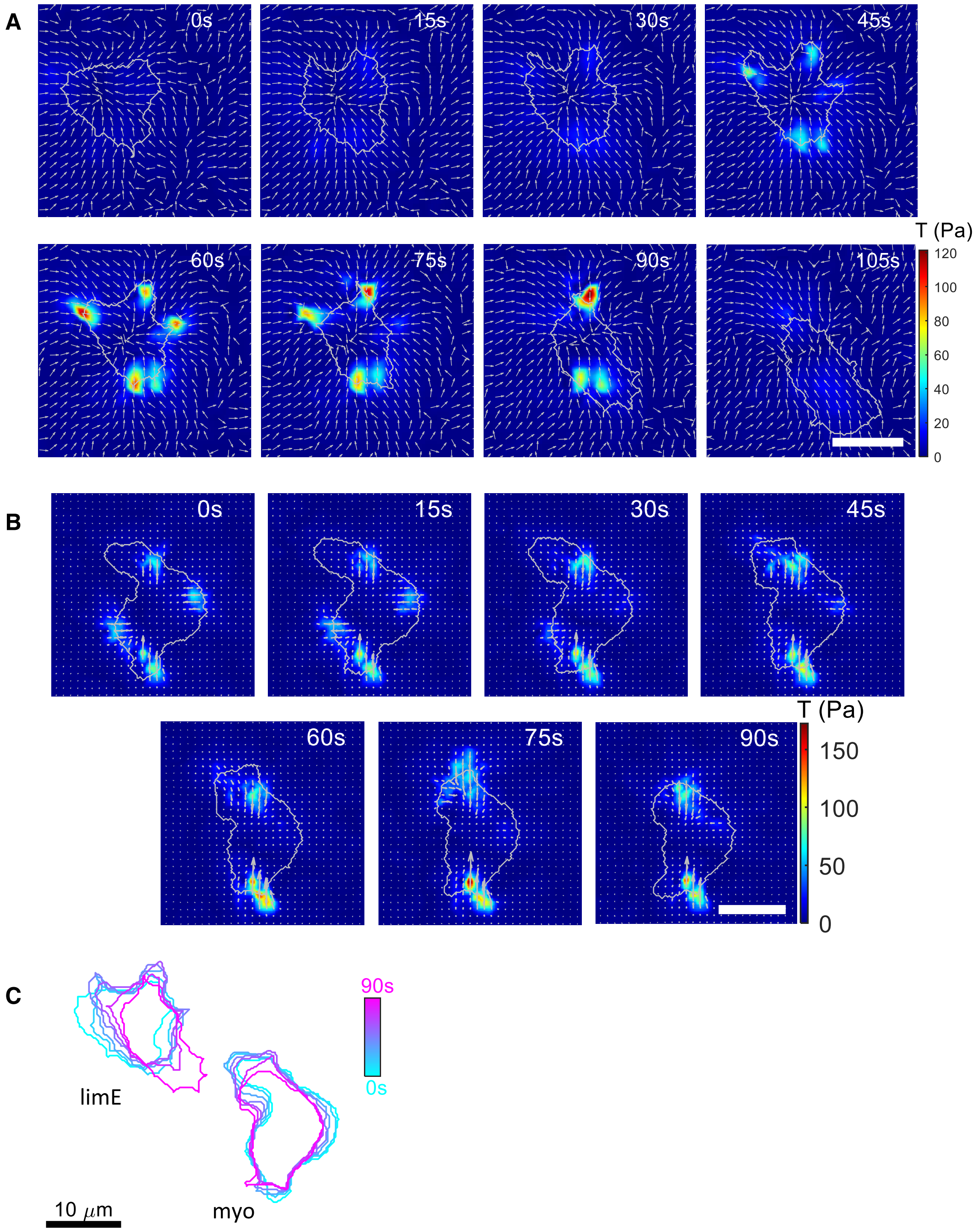

Figure EV4. Stress maps of an amoeboid cell.

A Stress maps of the same amoeboid cell as in Fig 4A top row. The arrows indicate only the direction of the stress, regardless of the magnitude. Forces are oriented inward at all times for the amoeboid and oscillatory modes.

B Stress map of the second cell of Fig 4A, expressing GFP-myo. The arrows are proportional to the local stress.

C Outlines of the cells presented in Fig 4, at the corresponding times.

Data information: All scale bars in the figure: 10 μm.

© 2021 The Authors
