# Supplementary material for: Coupling traction force patterns and actomyosin wave dynamics reveals mechanics of cell motion
Source: Mol Syst Biol. 2021 Dec 13;17(12):e10505. doi: 10.15252/msb.202110505 (PMC8666840; doi:10.15252/msb.202110505)
Supplement: Supplementary file 4 — Movie EV1 [file MSB-17-e10505-s002.zip › EV1_legend.docx]

Movie EV1: Movies of type 1 and 2 fan shaped cells expressing LimE-GFP.
