# Supplementary material for: Coupling traction force patterns and actomyosin wave dynamics reveals mechanics of cell motion
Source: Mol Syst Biol. 2021 Dec 13;17(12):e10505. doi: 10.15252/msb.202110505 (PMC8666840; doi:10.15252/msb.202110505)
Supplement: Supplementary file 5 — Movie EV2 [file MSB-17-e10505-s008.zip › EV2_legend.docx]

Movie EV2: Movies of type 1 and 2 fan shaped cells illustrating the distribution of the contractile protein myosin II, visualized using GFP-myo.
