# Supplementary material for: Coupling traction force patterns and actomyosin wave dynamics reveals mechanics of cell motion
Source: Mol Syst Biol. 2021 Dec 13;17(12):e10505. doi: 10.15252/msb.202110505 (PMC8666840; doi:10.15252/msb.202110505)
Supplement: Supplementary file 6 — Movie EV3 [file MSB-17-e10505-s004.zip › EV3_legend.docx]

Movie EV3: Stress maps of a type 1 fan shaped cell.
