# Supplementary material for: Coupling traction force patterns and actomyosin wave dynamics reveals mechanics of cell motion
Source: Mol Syst Biol. 2021 Dec 13;17(12):e10505. doi: 10.15252/msb.202110505 (PMC8666840; doi:10.15252/msb.202110505)
Supplement: Supplementary file 9 — Movie EV6 [file MSB-17-e10505-s001.zip › EV6_legend.docx]

Movie EV6: Stress maps (left panel) and GFP-myo distributions of an oscillatory cell.
