# Supplementary material for: Coupling traction force patterns and actomyosin wave dynamics reveals mechanics of cell motion
Source: Mol Syst Biol. 2021 Dec 13;17(12):e10505. doi: 10.15252/msb.202110505 (PMC8666840; doi:10.15252/msb.202110505)
Supplement: Supplementary file 12 — Movie EV9 [file MSB-17-e10505-s014.zip › EV9_legend.docx]

Movie EV9: Simulation results for a large contractile strength, resulting in a fan shaped cells consistent with type 1.
