# Supplementary material for: Coupling traction force patterns and actomyosin wave dynamics reveals mechanics of cell motion
Source: Mol Syst Biol. 2021 Dec 13;17(12):e10505. doi: 10.15252/msb.202110505 (PMC8666840; doi:10.15252/msb.202110505)
Supplement: Supplementary file 13 — Movie EV10 [file MSB-17-e10505-s009.zip › EV10_legend.docx]

Movie EV10: Simulation results for a small contractile strength, resulting in a fan shaped cell consistent with type 2.
