# Supplementary material for: Coupling traction force patterns and actomyosin wave dynamics reveals mechanics of cell motion
Source: Mol Syst Biol. 2021 Dec 13;17(12):e10505. doi: 10.15252/msb.202110505 (PMC8666840; doi:10.15252/msb.202110505)
Supplement: Supplementary file 16 [file MSB-17-e10505-s015.pdf]

molecular systems biology

Coupling traction force patterns and actomyosin wave dynamics reveals mechanics of cell motion

Elisabeth Ghabache, Yuansheng Cao, Yuchuan Miao, Alexander Groisman, Peter Devreotes, and Wouter-Jan Rappel
DOI: 10.15252/msb.202110505

Corresponding author(s): Wouter-Jan Rappel (rappel@physics.ucsd.edu)

Review Timeline:

Submission Date:	16th Jun 21
Editorial Decision:	22nd Jul 21
Revision Received:	18th Oct 21
Editorial Decision:	15th Nov 21
Revision Received:	18th Nov 21
Accepted:	19th Nov 21

Editor: Jingyi Hou

Transaction Report:

(Note: With the exception of the correction of typographical or spelling errors that could be a source of ambiguity, letters and reports are not edited. Depending on transfer agreements, referee reports obtained elsewhere may or may not be included in this compilation. Referee reports are anonymous unless the Referee chooses to sign their reports.)

Thank you for submitting your work to Molecular Systems Biology. We have now heard back from the three reviewers who agreed to evaluate your study. As you will see below, the reviewers find the topic of your study of interest. However, they raise substantial concerns about your work, which should be convincingly addressed in a major revision of the present manuscript.

Without reiterating all the points listed below, the most fundamental issues that need to be convincingly addressed are the following:

- Reviewer #1's concerns about the model need to be addressed. Attention should be paid to placing the current modeling approach in the context of existing dynamic modeling literature.

- Reviewer #2 pointed out that "migration" is not sufficiently addressed in the current study. During our pre-decision cross-commenting process (in which the reviewers are given the chance to make additional comments, including on each other's reports), Reviewers #1 and #3 concurred with Reviewer #2. In light of the comments from all three reviewers, we would ask you to tone done the conclusion about migration within reason.

All other issues raised by the reviewers need to be satisfactorily addressed as well.

On a more editorial level, we would ask you to address the following issues:

REFEREE REPORTS

Reviewer #1:

In their manuscript the authors study different motility modes
of dictyostelium cells, especially concerning their different dynamics of protrusion (actin) and retraction (myosin),
and in combination with traction forces measured by TFM.

While the different motility modes have been reported previously,
I am very much in favor of such a comparative study.
The experiments are thorough, the modelisation is a bit oversimplified,
but it can be understood that it is not the aim here to give a fully dynamic
self-consistent description of all motility modes.

In summary I am in favour of publication, after the authors have considered
by concerns and comments below.

1) From the main part of the manuscript it is not clear how the different motility modes are induced experimentally. This can be found in the supplement, but it should be briefly described in the main text, i.e. which methods induce fan shap vs. oscillatory vs. amoeboid for the used dictyostelium cells.

2) I am a bit worried about some of the traction force patterns, where the main contributions are on the boundary or even slightly outside of the cell (which would be unphysical) . Could the authors comment on this?
Did the they verify their TFM analysis using an alternative method?

3) Concerning the modeling: I can understand that the model is not fully dynamic, i.e. the actin/myosin is basically prescribed. However, I do not get some of the choices:
- when myosin and actin regions are seperated (cf. fan-shape type), this biologically makes not much sense to me: myosin alone (without actin) does not do anything. Do the authors in fact mean actomyosin, when they write myosin, and bare actin if they write actin ?
- in the experimental section, if I did get it correctly, the difference of type 1 and type 2 concerning actin is that the actin ring is directly underneath the membrane vs. further away from the membrane?
In the model implementation this is not reflected, why?
- clearly the oscillatory mode with area changes of 400% can not be described in a 2D model. There are 3D models available (even by the same authors, see Cao et al JRS INterface 2019, and also Winkler et al Commun. Phys 2019) that should be mentioned as future verification of the proposed minimal approach

4) The modeling reminds me of work by Alonso's group [Moreno et al (BTW, cited as 15 and 45)] and also of Aransons group (Reeves et al Commun. Phys 2018). The former is fully dynamic, but does not implement adhesion/traction forces. The latter does include traction and in fact the shape waves in there (and the effective Burgers-equation) are related to protrusion and concomittant traction forces. The authors should discuss the relation of their simple prescribe oscillatory actomyosin and more refinde models.

Reviewer #2:

Elisabeth Ghabache and colleagues investigate the dynamics of actin and traction forces in parallel to cell shape changes in three distinct models of migration recapitulating keratocyte gliding motion, oscillatory and amoeboid migration. While the mechanisms controlling each of these different modes of migration have been analyzed in numerous studies, their direct comparison is likely to bring a better understanding of the cell properties that control the mode of motion and the potential switch between each of these modes. In this context, this study is a nice attempt a precisely characterizing the characteristics of the three types of motion. This gives the opportunity to the authors to unravel two subtypes of gliding motions. The description of the actin waves, the dynamics of traction forces is done very carefully. While it is very unlikely that theses two parameters are the only ones involved in the different cell motility, it does show the similarities and differences between each migration types. The authors then use these data in a computational model that predicts the distribution of traction forces in response to the pattern of actin and myosin dynamics. Unfortunately, both the description of the cell dynamics and the computational model leave out the migration, which in principle was the goal of the study. I believe that this is a major problem that precludes publication. The authors may want to reword the text so that it is clear that it does touch on the migration but only on the membrane dynamics and cell motility, with the risk that the paper will severely loose impact.

General comments:
1- While the study aims at determining how actin dynamics and traction forces are coupled in different modes of migration, the parameters describing the migration in each case are not shown. In figures 2, 3 and 4 which respectively describe the keratocyte-like migration, the oscillatory and the amoeboid modes of migration, the authors should show the speed, the directionality and persistence of migration (showing, for instance, data similar to that of figure S1 with the quantification of the migration characteristics). The migration direction should be indicated for each of cell shown in figure 2, 3 and 4.
How do the distribution of actin dynamics, cell contractility and traction forces correlate with cell speed. For instance in the keratocyte-like migration, do the types I and II migrate with the same speed? We can expect the position of the actin ring in the type I to dictate the direction of migration. In the type 2, where the actin ring do not present a clear polarity, is it possible to detect a difference that may explain the localization of traction forces and the direction of migration? In figure 3 and 4, it is not even clear that the cells shown as example are effectively migrating.
2- The authors show a correlation between the intensity of traction forces and the cell size. Does cell size also correlates with cell speed?
3- The graphs showing the total amount of forces (Figure 1E for instance) seems to indicate the mean of the absolute values of the forces. Is the mean of the relative values always null? Could the authors show graph of the sum of the relative values of Tx

as a function of Y for type I and type2 as well as in the corresponding models? The total amount of forces is shown to increase with the cell area. Does this mean that the total forces normalized by the area (pressure) is identical for all cell types? In figure 1B, it seems that the traction forces in type 2 cells are localized very close to the extremities ('left' and 'right') of the cell, while they appear more concentrated at the rear edge of the cell (assuming cells are migrating towards to the top of the figure). Is this observed for all type 1 and type 2 cells or is it only the case for the 2 cells shown here (in which case, the authors should show another more representative example)
4- The authors use GFP-myosin as an indicator of the localization of contractile forces in cells. Phosphorylation of myosin is a key indicator of the activity of the myosin motor but is not shown here. For each type of motion, the authors should show high resolution (no need for superresolution) images of the actin, phosphor-myosin and focal adhesion staining to better document the organization of the stress fibers.
5- Do the type I and type II of keratocyte-like migration correspond to two distinct population of cells or can the same cells switch from one type to the other with time? If it is correlated with cell size, could it be associated with different phases of the cell cycle?
6- I am not sure I clearly understood what information does the model bring. It does recapitulate some of the observations made in cells, but it seems to use very well known facts (actin polymerization leads to membrane protrusion, acto-myosin activity leads to cell contraction and traction forces) and by adding the parameters observed in cells, it does show similar motility. It is possible that a more detailed explanation of what the new findings may be sufficient. The authors should also show the cell edge velocity predicted by the model and recapitulating the data of the figure 5A.
7- Can the model show cell migration? And, if so, does the migration have the same characteristics to that of cells? The purpose of this study being to better understand the different types of migration and how cells can switch from one to another, it seems that the model should be able to show migration and determine which key parameter (localization, intensity of contractility, temporal correlation function...) would allow the switch between different types of motion.
8- The model is in fact quite different from the observations in type 2 keratocyte-like type of motion. In this case, the actin ring should be detached from the membrane all around the cell and not only at the back. The traction forces oriented towards the front at the back of the cell should not be localized along the central axis but rather on each side of the read edge (as shown in Figure 1B, Tx).
9- The model is also quite different from the observations in the case of the oscillatory motion, for which it predicts a homogenous contractility all along the cell periphery while it localizes in discrete spots in the cell shown in figure 3A.
Minor comments
1- In figures 1E, 4L, 4M. It is difficult to distinguish the shape of the dots.
2- Figure 3, there is problem with the panel numbers. The authors should check the legend of the figure.
3- Figure 3, the oscillation period seems to vary between different cells (for instance in 3B and 3C). How much does it vary from cell to cell? Does it vary in time or as a function of the cell area?

Reviewer #3:

Summary:
In this paper, the authors study the correlation between force patterns generated by cells displaying different types of migratory behaviors and the corresponding actomyosin spatiotemporal distribution. To this end, they use Dictyostelium discoideum cells, which can undergo different modes of migration depending on their culture condition: keratocyte-like, oscillatory, or amoeboid modes. They use a combination of imaging techniques to follow both actomyosin labels and traction force overtime, and modelling to try and simulate the traction force patterns generated by the cells in the different modes.
They first show that Fan-shaped cells display constant actomyosin waves propagating during migration and that forces exhibit two main poles at the back of the cells. Among Fan-shaped cells, they characterize two distinct types, for which the actin distribution pattern is different, leading to a differential effect on cell protrusion and thus traction stress pattern. This difference also results in a significant change in cell size between the two types.
They then demonstrate that cell oscillatory behavior occurs following the initial presence of stable actin waves propagating along the membranes and creating cell expansion followed by myosin waves preceding cell retraction. This spatiotemporal sequence results in oscillations of the inward directed forces and the cell size.
The authors next study the amoeboid cell migration behavior and show that in this case random and non-persistent actin waves occur and disappear leading to non-synchronized phases of expansion and retraction.
Finally, the authors extract spatiotemporal information on the cell edge velocity, actomyosin intensities and distribution from kymographs and compute all this information in a mathematical model aiming at reproducing the traction force patterns of all 3 types of migrating cells. By modelling the spatiotemporal distribution of actin and myosin and varying the strength of protrusion, the authors could reproduce the force patterns corresponding to the different migratory behaviors.

General comments:
Overall, the manuscript is good, well written and clearly displays the results. Although not entirely novel, as previous work already studied traction force patterns in cell displaying different types of migration, the work performed in this study offers a full characterization of both the cellular actomyosin spatiotemporal distribution and the force patterns generated by these cells, providing a comprehensive understanding of their relationship. The conclusions of the work are well supported by the data presented. In addition, it provides significance that this study is conducted using one common cellular model to study the

different types of migratory behaviors, avoiding the heterogeneity due to the use of different cell types. Finally, the model displaying a rather limited number of parameters is also in good agreement with the experimental conclusions of the work, supporting the hypothesis that both the force pattern and cell size rely on the spatiotemporal distribution of actin and myosin in cells and on the resulting balance between protrusive and contractile forces. As such, the manuscript can be published with only minor revisions detailed below.

Minor points that should be addressed or discussed:

• From a technical point of view, the authors use silicone gels of 1kPa having a very thin width (3-15 µm), on which they measure displacements in a range of 300 nm (which represent 10% of the gel thickness). Due to this small thickness and the use of a very soft gel, the gel may not display a linear elastic response and the glass below probably affects the actual stiffness of the gel, resulting in a loss of accuracy of the traction force measured. This error probably doesn't have a major effect on the work conclusions as the measures for the 3 different types of migrating cells are performed in the same conditions, however, the authors could try to measure the elasticity of the gels using some complementary methods like AFM to ensure that they have the expected 1 kPa.
• Displaying data originating from cells in which both actin and myosin are concomitantly labelled would facilitate the understanding of the spatiotemporal distribution of the two proteins and their correlation with the stress patterns (for example, the two cells taken as an example in Fig3B and C display very different oscillation periods). Along the same line, it would be helpful to display the force map and the myosin content from the same cells (Figure 3D kymograph is associated to the cell for which actin only is displayed in Fig3B so it makes things difficult to compare the pattern of myosin with the pattern of force). If technically not possible to perform dual labelling in the cells together with TFM, the authors could display instead one example of a cell (and associated kymographs) with actin and force and another example of cell with myosin and force as they did in Figure 4F to K.
• Type 1 and 2 descriptors are inverted in movies S1/S2
• The authors describe that the forces at the front of the cells are directed forward (in the direction of the movement) but this is not really visible on the different illustrations shown (Figure 2B and S4A and C). Forces seem rather to orient in a parallel fashion to the membrane, which is consistent with the vortices described by the authors.
• In Fig 2E: the symbol colors are inverted as compared to the figure caption
• Line 144: the sentence is unclear (a verb is missing)
• In the conclusion (line 376), the authors state that the temporal correlation between actomyosin and traction force was conserved across the different modes, suggesting that the modes employ the same migration mechanisms. This could be true only because the authors used the same cellular model for assessing all types of migration. It may be different in different cell types.
• Several plots displaying total force as a function of cell area show that they increase with cell area. However, the authors show that in different types of migration, the traction stresses are maximal during cell retraction phases, which appears to be in apparent contradiction with previous data. Could the authors comment on that?

Dear Editor,

We thank the Reviewers and you for your careful consideration of our study. As a response of these comments, we have further detailed the migration aspects of our study. Specifically, we have added a new figure (Fig. 6), which schematically summarizes our experimental findings. In addition, we have now placed our simulation results in a broader context. Finally, we have designated 4 figures as Extended View figures and one table as Extended View Table and have placed the computational code and datasets online.
Please find below our detailed reply to the comments of the Reviewers.

Reviewer #1:

In their manuscript the authors study different motility modes of dictyostelium cells, especially concerning their different dynamics of protrusion (actin) and retraction (myosin), and in combination with traction forces measured by TFM.

While the different motility modes have been reported previously, I am very much in favor of such a comparative study. The experiments are thorough, the modelisation is a bit oversimplified, but it can be understood that it is not the aim here to give a fully dynamic self-consistent description of all motility modes.

In summary I am in favour of publication, after the authors have considered by concerns and comments below.

We thank the Reviewer for these encouraging remarks and are happy to see that he/she is in favor of publication.

1) From the main part of the manuscript it is not clear how the different motility modes are induced experimentally. This can be found in the supplement, but it should be briefly described in the main text, i.e. which methods induce fan shap vs. oscillatory vs. amoeboid for the used dictyostelium cells.

We apologize for this omission in the main text, due to constraints on the length of the manuscript. We have now included the description how we induced the different migration modes in the cells in the Methods section, which is now part of the main text.

2) I am a bit worried about some of the traction force patterns, where the main contributions are on the boundary or even slightly outside of the cell (which would be unphysical) . Could the authors comment on this? Did the they verify their TFM analysis using an alternative method?

We thank the Reviewer for this comment. To obtain a traction force map from a map of bead displacements one needs to solve an inverse problem. One technique to solve this inverse problem, traction reconstruction with point forces (TRPF), can be used for mammalian cells and uses information about the discrete locations of focal adhesion complexes. This method specifies that the traction forces are applied at these locations and then sets the traction forces everywhere else to zero. Unlike mammalian cells, however, Dictyostelium cells do not have focal adhesion complexes. Therefore, it is unclear at which discrete sites the cell transmit forces to the substrate, necessitating a different approach than TRPF. Following previous studies (Sabass et al. 2008, Han et al. 2015), we decided to use the boundary element method (BEM), which treats surface forces and displacements as continuous fields. It is important to note that this, and any other method that not explicitly identifies the locations of force sites, unavoidably produces forces that extend slightly outside the physical footprint of the cells.. In some publications, only forces within the cell's footprint are shown, artificially setting all forces outside the cell to zero. In our study, however, we report the entire traction force map, including any "bleeding" into areas outside the cell. Please note that our approach affords us a natural consistency check: the extent of our

traction force map should be well correlated with the cell's footprint. In other words, the computed traction force map should be consistent with the cell footprint. We have validated this consistency check is satisfied for our maps. Finally, we note that we have also computed the traction force map using an alternative method, the Fourier Transform Traction Cytometry method (FTTC) (Butler et al, 2002). The results are qualitatively consistent with the ones presented in the paper, as can be seen from the new Appendix Fig. S2B.

To add in the text:
In our force maps, cells appear to exert traction forces in areas outside their physical boundaries. This appearance of non-zero forces outside the cells is due to the finite spatial resolutions of both the tracer particle displacement map and the conversion of the displacement map into the traction force map and is inherent to force reconstruction methods that do not have any constraints on where traction forces are exerted. Thus, unlike some methods explicitly postulating that traction forces are only applied at the adhesion complexes within the cell footprint, e.g, Traction Reconstruction with Point Forces [58], our procedure will always result in traction force maps with non-zero forces just outside of the cell footprint. We should also point out that a different computational technique of obtaining the traction force map, the Fourier Transform Traction Cytometry method (FTTC)[60], gives qualitatively similar results (Appendix Fig. S2B).

3) Concerning the modeling: I can understand that the model is not fully dynamic, i.e. the actin/myosin is basically prescribed. However, I do not get some of the choices: - when myosin and actin regions are seperated (cf. fan-shape type), this biologically makes not much sense to me: myosin alone (without actin) does not do anything. Do the authors in fact mean actomyosin, when they write myosin, and bare actin if they write actin ?

We apologize for the possible confusion and thank the Reviewer for this suggestion. Our marker for actin (LimE) indicates the location for freshly polymerized actin. Therefore, the absence of this marker does not necessarily mean that that there is no actin present at that location. This is also evident from our Life-Act results, which visualize all actin (Appendix Figs. S1, S16, S20). Furthermore, myosin is concentrated in the back of the fan-shaped cells, but is also present in the remainder of the cell body (see Fig. 2).
We intended our model to be as simple as possible. To this end, we assumed that the LimE-labeled actin distribution is able to generate protrusive forces while myosin can produce contractile forces, even where these is no LimE-labeled actin, because actin is actually present in the entire cell. We have now made this modification in the main text. Specifically, we have added:

"Our actin distribution represents freshly polymerized actin, visualized in the experiments using LimE, but we assume that actin filaments are distributed over the entire cell, providing a substrate for myosin."

- in the experimental section, if I did get it correctly, the difference of type 1 and type 2 concerning actin is that the actin ring is directly underneath the membrane vs. further away from the membrane? In the model implementation this is not reflected, why?

The Reviewer is correctly pointing out that there is a subtle difference in the actin localization in the two cell types. In our simplified model, the membrane is driven by actin protrusions and therefore follows the distribution of actin. The subtle difference between type 1 and 2 cells suggests that in type 2 cells the freshly polymerized actin ring is likely separated from the membrane by a rigid cytoskeletal network. This detail is not included in the model.

- clearly the oscillatory mode with area changes of 400% can not be described in a 2D model. There are 3D models available (even by the same authors, see Cao et al JRS INterface 2019, and also Winkler et al Commun. Phys 2019) that should be mentioned as future verification of the proposed minimal approach

We fully agree with the Reviewer and have now added these references, along with a short description in the Discussion:

"A further extension of the model that could potentially verify some of our results is to render cells as three dimensional objects, as was carried in recent studies [16,54]."

4) The modeling reminds me of work by Alonso's group [Moreno et al (BTW, cited as 15 and 45)] and also of Aransons group (Reeves et al Commun. Phys 2018). The former is fully dynamic, but does not implement adhesion/traction forces. The latter does include traction and in fact the shape waves in there (and the effective Burgers-equation) are related to protrusion and concomittant traction forces. The authors should discuss the relation of their simple prescribe oscillatory actomyosin and more refinde models.

We agree with the Reviewer. We have now added these references and have included a discussion of their results. Specifically, we have added:

"Thus, our modeling approach is different from previous studies that solve reaction-diffusion equations to obtain the distributions of signaling components [15,16,46]. However, since these previous studies have demonstrated that the essential wave dynamics of these distributions can be obtained using computational models we are able to use them as inputs [15,16,46]. Future work could include combining these models with the framework we have presented here. A further extension of the model that could potentially verify some of our results is to render cells as three dimensional objects, as was carried in recent studies [16,54]. Also note that we have not incorporated the explicit dynamics of adhesion bonds as in some previous results [41,55]"

Reviewer #2:

Elisabeth Ghabache and colleagues investigate the dynamics of actin and traction forces in parallel to cell shape changes in three distinct models of migration recapitulating keratocyte gliding motion, oscillatory and amoeboid migration. While the mechanisms controlling each of these different modes of migration have been analyzed in numerous studies, their direct comparison is likely to bring a better understanding of the cell properties that control the mode of motion and the potential switch between each of these modes. In this context, this study is a nice attempt a precisely characterizing the characteristics of the three types of motion. This gives the opportunity to the authors to unravel two subtypes of gliding motions. The description of the actin waves, the dynamics of traction forces is done very carefully. While it is very unlikely that theses two parameters are the only ones involved in the different cell motility, it does show the similarities and differences between each migration types. The authors then use these data in a computational model that predicts the distribution of traction forces in response to the pattern of actin and myosin dynamics. Unfortunately, both the description of the cell dynamics and the computational model leave out the migration, which in principle was the goal of the study. I believe that this is a major problem that precludes publication. The authors may want to reword the text so that it is clear that it does touch on the migration but only on the membrane dynamics and cell motility, with the risk that the paper will severely loose impact.

We thank the Reviewer for this comment. In our revised version, we have now included a better description of the migration properties of the cells. Please see our detailed answers below. In addition, we have included a new figure (Fig. 6), which schematically "summarizes" our experimental results. This figure is also described in the text:

"Our experiments suggest the following scenario, shown schematically in Fig. 6. Actin polymerization is responsible for membrane protrusions and is controlled by the wave dynamics: stable waves propagating with

the speed of the cell for fan-shaped cells (Fig. 6B), target waves propagating outwardly for oscillatory cells (Fig. 6C), and unstable waves in the case of amoeboid cells (Fig. 6D). For all migration modes, once an actin wave reaches the cell membrane, it "pushes off" against it, generating a cytoskeletal flow that is directed inward. Due to friction with the substrate, this flow creates traction forces that are also directed inward (Fig. 6A). Myosin is responsible for contraction and pulls on the membrane. As a result, traction forces are generated that are also pointing inward (Fig. 6B).
For fan-shaped cells, myosin is along most of the nearly straight membrane at the back of the cell (Fig. 6C). Since myosin contracts along this entire band, the traction forces are largest at the end points, located at the rear corners of the cell. The generated cytoskeletal flow created by the contractile myosin and the protrusive actin then leads to the cell-wide traction force patterns that is different for the two types of cells. Specifically, when myosin is dominant, contractile forces generate a swirling flow pattern and push the cytoskeleton forward in the entire cell (type 1 cell). For type 2 cells, myosin creates forward directed flow at the rear while actin polymerization results in backward oriented flow at the front. For oscillatory cells, the contractile forces generated by myosin start after the actin ring has moved away from the basal plane, contracting the cell at the basal surface (Fig. 6C). Finally, for amoeboid cells, myosin is creating contractions that retract pseudopods, which result in traction forces at the base of pseudopods (Fig. 6D)."

General comments:

1- While the study aims at determining how actin dynamics and traction forces are coupled in different modes of migration, the parameters describing the migration in each case are not shown. In figures 2, 3 and 4 which respectively describe the keratocyte-like migration, the oscillatory and the amoeboid modes of migration, the authors should show the speed, the directionality and persistence of migration (showing, for instance, data similar to that of figure S1 with the quantification of the migration characteristics). The migration direction should be indicated for each of cell shown in figure 2, 3 and 4.

To make the migration parameters clearer we have incorporated Fig. S1 from our original submission, showing the outlines of the cells presented in Figs. 2-4 at different times, into the updated Fig.1. Furthermore, we have indicated the migration direction for each of the cells in Figs. 2-4. In addition, we now state the speed of these particular cells in the legends and refer to a supplemental figure (Appendix Fig. S21) for the ensemble average of speeds. Please note that the migration characteristics of the different cell types were already reported by Miao et al, 2017. Specifically, they reported the cell migration speed as well as the directedness for the different cell types in their Fig. 1.

How do the distribution of actin dynamics, cell contractility and traction forces correlate with cell speed. For instance in the keratocyte-like migration, do the types I and II migrate with the same speed? We can expect the position of the actin ring in the type I to dictate the direction of migration. In the type 2, where the actin ring do not present a clear polarity, is it possible to detect a difference that may explain the localization of traction forces and the direction of migration? In figure 3 and 4, it is not even clear that the cells shown as example are effectively migrating.

We thank the Reviewer for this comment. We have now created graphs detailing the speed of the cells as a function of basal area and total force for both types of fan-shaped as well as for the amoeboid and oscillatory cells (Appendix Fig. S4A). For all migration modes, neither the area nor the total force appears to be strongly correlated with the cell speed. Please note, however, that the speed of type 1 and type 2 fan shaped cells were significantly different (as can be seen from panel A and B). We also appreciate the comment that the actin ring in the type 2 cells does not show a clear polarization. However, actin is not the only biochemical component

that determines the migration direction, and the intracellular distribution of myosin shows a clear polarization. We have added a sentence to make this clearer:

"Furthermore, the GFP-myo kymographs showed a region of high fluorescence at the back of the cell (Fig. 2C, lower row), thus indicating a clear symmetry breaking and polarization in the cell."

Finally, we agree that the motion of cells in figure 3 and 4 was not very clear. This was because each snapshot was centered on the center of mass of the cell at each given time. We have now changed the presentation, using the laboratory reference frame instead. We also added white arrows to show the direction of motion of the cell in each snapshot. In addition to showing the direction of motion for the amoeboid cell, this new presentation highlights the fact that the oscillatory cell does not exhibit appreciable migration during the contraction/extension phase and only briefly migrates between the two cycles of contraction/extension. These migration dynamics are also evident from the outlines of the cells in Fig. 1 and is consistent with earlier work (Miao et al, 2017).

2- The authors show a correlation between the intensity of traction forces and the cell size. Does cell size also correlates with cell speed?

Please see our response to point #1. The plot showing the cell speed as a function of cell size (i.e., area) for both the amoeboid and the keratocyte-like cells is now included as Appendix Fig. S4.

3- The graphs showing the total amount of forces (Figure 1E for instance) seems to indicate the mean of the absolute values of the forces. Is the mean of the relative values always null? Could the authors show graph of the sum of the relative values of Tx as a function of Y for type I and type2 as well as in the corresponding models? The total amount of forces is shown to increase with the cell area. Does this mean that the total forces normalized by the area (pressure) is identical for all cell types? In figure 1B, it seems that the traction forces in type 2 cells are localized very close to the extremities ('left' and 'right') of the cell, while they appear more concentrated at the rear edge of the cell (assuming cells are migrating towards to the top of the figure). Is this observed for all type 1 and type 2 cells or is it only the case for the 2 cells shown here (in which case, the authors should show another more representative example)

We thank the Reviewer for this question. The sum of the relative values of the force in our maps is very close to zero, which is expected from the basic mechanical considerations and is consistent with previous traction force studies (e.g., Bastounis et al, 2014). For example, comparing the total force F defined using the absolute values of the stress to the total force f defined as the relative values of the stress for the two cells displayed in figure 2B&D, we found a difference of more than 1 order of magnitude: 3.7nN instead of 0.3nN for the cell type 1 (fig. 2B) and 21.6nN instead of 1.8nN for the cell type 2 (fig2D). (The value of f is not exactly zero because of the numerical noise, but our signal to noise ratio F/f is >10.)
As requested, we now include in the Appendix a graph of the sum of T_x (and T_y) as a function of y for these two cells (Appendix Fig. S3).

We have also determined the average pressure (characteristic stress) of the cell as a function of the area (Appendix Fig. S5). For each of the three migration modes, the pressure is largely independent of the area, indicating that the total amount of forces increases with the area. The average values of the pressure are somewhat different for different modes.

The Reviewer is right in observing that the traction forces of the type 2 cell presented in the paper are more concentrated at the extremities of the cells. This is representative for all type 2 cells as can be deduced from the ratio of the pole-to-pole distance and the cell's length. This measurement, reported in the legend of fig. 2, is

significantly different for type 1 and type 2 cells: “the median ratio between the pole-pole distance and the cell's length was 0.75 (0.70/0.79, N=161) for type 1 cells and 0.84 (0.77/0.90, N=12) for type 2 cells ($p=2.2\ 10^{-3}$)”

4- The authors use GFP-myosin as an indicator of the localization of contractile forces in cells. Phosphorylation of myosin is a key indicator of the activity of the myosin motor but is not shown here. For each type of motion, the authors should show high resolution (no need for superresolution) images of the actin, phosphor-myosin and focal adhesion staining to better document the organization of the stress fibers.

Please note that Dictyostelium cells do not contain focal adhesion sites nor do these cells display stress fibers. In fact, adhesion between Dictyostelium cells and the substrate is thought to be non-specific (see Loomis et al, 2012). Hence, it is not feasible to document the organization of stress fibers.

5- Do the type I and type II of keratocyte-like migration correspond to two distinct population of cells or can the same cells switch from one type to the other with time? If it is correlated with cell size, could it be associated with different phases of the cell cycle?

This is an interesting question. In our experiments, we have never observed a switch from one type to another. However, this does not mean that such a switch cannot occur. After all, our experimental field of view only allows us to follow a fast-moving keratocyte for approximately 10 min. Thus, we can only follow these cells for a limited amount of time and would not able to observe a switch occurring outside the field of view. Please also note that these cells are deprived of food and are no longer dividing.
We have added the following text:

“Although we have never observed a transition between the two cell types, we cannot rule it out since we can only follow cells for up to approximately 10 minutes.”

6- I am not sure I clearly understood what information does the model bring. It does recapitulate some of the observations made in cells, but it seems to use very well known facts (actin polymerization leads to membrane protrusion, acto-myosin activity leads to cell contraction and traction forces) and by adding the parameters observed in cells, it does show similar motility. It is possible that a more detailed explanation of what the new findings may be sufficient. The authors should also show the cell edge velocity predicted by the model and recapitulating the data of the figure 5A.

The aim of the model is to see if it is possible to reproduce the traction force patterns for all three migration modes by simply changing the wave dynamics and spatial location of actin and myosin. Please note that our model can fully recapitulate the experimental data and can provide insights into the balance between protrusive and contractile forces for the fan-shaped models (see above). We have now added text that summarizes the experimental data, which is addressed by the model. Following the suggestion of the Reviewer, the migration speed and edge velocity obtained from the model are now reported in more detail and are consistent with experimental results (see legend of Fig. 7).

7- Can the model show cell migration? And, if so, does the migration have the same characteristics to that of cells? The purpose of this study being to better understand the different types of migration and how cells can switch from one to another, it seems that the model should be able to show migration and determine which key parameter (localization, intensity of contractility, temporal correlation function...) would allow the switch between different types of motion.

Absolutely, the model does show migration. To make this clearer, we have now added movies of our simulations, which show the dynamics of the cell (Movie EV9-12). These movies show that the simulated migration is consistent with the experimentally observed migration.

8- The model is in fact quite different from the observations in type 2 keratocyte-like type of motion. In this case, the actin ring should be detached from the membrane all around the cell and not only at the back. The traction forces oriented towards the front at the back of the cell should not be localized along the central axis but rather on each side of the read edge (as shown in Figure 1B, Tx).

The first part of this comment is similar to the comment of Reviewer 1 and we refer to our reply to point #2 of this Reviewer. To further compare the traction force patterns obtained in the experiments and in the model, we have now plotted T_x for both simulated cell types. The resulting graph (Appendix Fig. S22) is in good qualitative agreement with the experiments. Specifically, the orientation of the traction forces shown in Fig. S22 are consistent with the ones shown in Fig. 1.

9- The model is also quite different from the observations in the case of the oscillatory motion, for which it predicts a homogenous contractility all along the cell periphery while it localizes in discrete spots in the cell shown in figure 3A.

We agree with the Reviewer that the distributions obtained from our model are simpler and "cleaner" than those in the actual experiments. However, the main goal of the model is to show how a plausible distribution of actin and myosin can lead to the observed traction force pattern. Our model is intended be simple, and can be easily modified to include a more complex distribution of actin. However, including a less homogeneous version of the actin distribution will not result in qualitatively different results. This is shown in the new Appendix Fig. S23, where we have introduced several spatially heterogeneous spots of actin. As long as the actin and myosin are synchronized, the simulated cell still shows cyclic retraction and extension phases, together with relatively strong traction force during retraction and weaker inward directed traction forces during expansion.

Minor comments

1- In figures 1E, 4L, 4M. It is difficult to distinguish the shape of the dots.

We thank the Reviewer for pointing this out. We have now simplified these figures and no longer differentiate between different strains. Therefore, all symbols are the same and different colors represent the different cell types. The complex figures showing all strains are now included in the Appendix (Appendix Fig. S4-6), which can be easily enlarged on the reader's computer.

2- Figure 3, there is problem with the panel numbers. The authors should check the legend of the figure.

Thank you. We have corrected this.

3- Figure 3, the oscillation period seems to vary between different cells (for instance in 3B and 3C). How much does it vary from cell to cell? Does it vary in time or as a function of the cell area?

The Reviewer is correct in pointing out that the oscillation period varies from cell to cell. This variation is captured in the Appendix Fig. S7: the mean period is 3.5 (2.6/4.4) min. However, it does not systematically vary with time for a given cell, as can be see from the examples of cells oscillating over extended time intervals (Appendix Fig. S8). We have now added an additional figure (Appendix Fig. S7D), illustrating that this period

does not have a strong dependence on the cell area (defined as the maximum area achieved during an oscillation).

Reviewer #3:

Summary:
In this paper, the authors study the correlation between force patterns generated by cells displaying different types of migratory behaviors and the corresponding actomyosin spatiotemporal distribution. To this end, they use Dictyostelium discoideum cells, which can undergo different modes of migration depending on their culture condition: keratocyte-like, oscillatory, or amoeboid modes. They use a combination of imaging techniques to follow both actomyosin labels and traction force overtime, and modelling to try and simulate the traction force patterns generated by the cells in the different modes.
They first show that Fan-shaped cells display constant actomyosin waves propagating during migration and that forces exhibit two main poles at the back of the cells. Among Fan-shaped cells, they characterize two distinct types, for which the actin distribution pattern is different, leading to a differential effect on cell protrusion and thus traction stress pattern. This difference also results in a significant change in cell size between the two types. They then demonstrate that cell oscillatory behavior occurs following the initial presence of stable actin waves propagating along the membranes and creating cell expansion followed by myosin waves preceding cell retraction. This spatiotemporal sequence results in oscillations of the inward directed forces and the cell size. The authors next study the amoeboid cell migration behavior and show that in this case random and non-persistent actin waves occur and disappear leading to non-synchronized phases of expansion and retraction. Finally, the authors extract spatiotemporal information on the cell edge velocity, actomyosin intensities and distribution from kymographs and compute all this information in a mathematical model aiming at reproducing the traction force patterns of all 3 types of migrating cells. By modelling the spatiotemporal distribution of actin and myosin and varying the strength of protrusion, the authors could reproduce the force patterns corresponding to the different migratory behaviors.

General comments:

Overall, the manuscript is good, well written and clearly displays the results. Although not entirely novel, as previous work already studied traction force patterns in cell displaying different types of migration, the work performed in this study offers a full characterization of both the cellular actomyosin spatiotemporal distribution and the force patterns generated by these cells, providing a comprehensive understanding of their relationship. The conclusions of the work are well supported by the data presented. In addition, it provides significance that this study is conducted using one common cellular model to study the different types of migratory behaviors, avoiding the heterogeneity due to the use of different cell types. Finally, the model displaying a rather limited number of parameters is also in good agreement with the experimental conclusions of the work, supporting the hypothesis that both the force pattern and cell size rely on the spatiotemporal distribution of actin and myosin in cells and on the resulting balance between protrusive and contractile forces. As such, the manuscript can be published with only minor revisions detailed below.

We thank the Reviewer for this positive assessment!

Minor points that should be addressed or discussed:

• From a technical point of view, the authors use silicone gels of 1kPa having a very thin width (3-15 µm), on which they measure displacements in a range of 300 nm (which represent 10% of the gel thickness). Due to this small thickness and the use of a very soft gel, the gel may not display a linear elastic response and the glass

below probably affects the actual stiffness of the gel, resulting in a loss of accuracy of the traction force measured. This error probably doesn't have a major effect on the work conclusions as the measures for the 3 different types of migrating cells are performed in the same conditions, however, the authors could try to measure the elasticity of the gels using some complementary methods like AFM to ensure that they have the expected 1 kPa.

We characterized the mechanical properties of the silicone gel used in our experiments with a special device, a centrifugal microscopy-based rheometer, which was previously designed and extensively tested in the Groisman lab (Ronan, E., "Centrifugal Rheometry and Rapid Stimulation of Dinoflagellate Bioluminescence in a Microfluidic Device", PhD Dissertation, UC San Diego, 2018). The tests included comparisons with the established techniques for measurements of the mechanical properties of soft materials (Gutierrez, E., and Groisman A., "Measurements of Elastic Moduli of Silicone Gel Substrates with a Microfluidic Device", PLoS One (2011)). The centrifugal rheometer applies a known shear stress, τ, to a uniform layer of gel and measures the resulting shear of the gel, γ, making it possible to calculate the shear modulus of the gel, G, as $G = \tau/\gamma$. The elastic modulus (Young's modulus) of the gel is then calculated as $E=2(1+ \mu)G$ where μ is the Poisson ratio, which is equal to ~0.5 for soft silicone gels, leading to $E{\sim}3G=3\ \tau/\gamma$. Please note that measuring the elastic modulus using AFM is more challenging and often produces artifacts because of the adhesion and Van der Waals interactions between the AFM tip and the gel. Furthermore, unlike hydrogels, silicone gels tend to stick to AFM tips, making silicone gels generally less suitable for AFM measurements than hydrogels.
Our measurements of the elastic modulus of the silicone gel (now included as Appendix Fig. S24) indicate a linear elastic response in the entire measurement range, even for values of γ as high as 48%, far above the maximal shear in our experiments. It is worth noting that the type of data in Appendix Fig. S24 (a linear dependence of γ on τ for γ of up to 48%) would be impossible to obtain with an AFM.
The Reviewer is absolutely correct that cells may deform our thin gel layers significantly less than they would have deformed a layer with a very large thickness. Please note that to account for the finite thickness of our gel layers, we used a modified version of the algorithm that converts bead displacement maps into traction stress maps, proposed by Merkel et al. (Merkel, R., Kirchgeßner, N., Cesa, C. M. and Hoffmann, B. "Cell Force Microscopy on Elastic Layers of Finite Thickness", Biophysical Journal 93, 3314-33-23 (2007)).

• Displaying data originating from cells in which both actin and myosin are concomitantly labelled would facilitate the understanding of the spatiotemporal distribution of the two proteins and their correlation with the stress patterns (for example, the two cells taken as an example in Fig3B and C display very different oscillation periods). Along the same line, it would be helpful to display the force map and the myosin content from the same cells (Figure 3D kymograph is associated to the cell for which actin only is displayed in Fig3B so it makes things difficult to compare the pattern of myosin with the pattern of force). If technically not possible to perform dual labelling in the cells together with TFM, the authors could display instead one example of a cell (and associated kymographs) with actin and force and another example of cell with myosin and force as they did in Figure 4F to K.

We agree with the Reviewer that data showing the concomitant labeling of actin, myosin, and traction force would be very useful. Unfortunately, as the Reviewer already guessed, it is technically not possible to carry this type of experiments. Nevertheless, we have followed the Reviewer's suggestion and now show examples of myosin labeled cells, together with their force maps (Fig. EV2 and Fig. EV4).

• Type 1 and 2 descriptors are inverted in movies S1/S2

We thank the Reviewer for pointing out this mistake, which is now corrected.

• The authors describe that the forces at the front of the cells are directed forward (in the direction of the

movement) but this is not really visible on the different illustrations shown (Figure 2B and S4A and C). Forces seem rather to orient in a parallel fashion to the membrane, which is consistent with the vortices described by the authors.

Please note that the important quantity is the x-component of T in Fig. 2. This x-component is, on average, pointing in the direction opposite of the motion, although the data can show some variability. To make this clearer, we have chosen different frames in the figure, which now clearly shows that the forces are pointing backwards (please see also Fig. EV1).

• In Fig 2E: the symbol colors are inverted as compared to the figure caption

Corrected. Thank you!

• Line 144: the sentence is unclear (a verb is missing)

Corrected. Thank you!

• In the conclusion (line 376), the authors state that the temporal correlation between actomyosin and traction force was conserved across the different modes, suggesting that the modes employ the same migration mechanisms. This could be true only because the authors used the same cellular model for assessing all types of migration. It may be different in different cell types.

We fully agree with the Reviewer. We have now included that this is true for Dictyostelium cells (and therefore not necessarily for other cell types).

• Several plots displaying total force as a function of cell area show that they increase with cell area. However, the authors show that in different types of migration, the traction stresses are maximal during cell retraction phases, which appears to be in apparent contradiction with previous data. Could the authors comment on that?

We apologize for this confusion. The plots of the total force as a function of cell area display the time-averaged total force and not the instantaneous force. The former increases with cell area while the latter is largest during retraction. We have made this clearer in the text by explicitly mentioning that the total force in these plots is time-averaged.

Thank you for sending us your revised manuscript. We have now heard back from the three reviewers who were asked to evaluate your study. As you will see below, the reviewers are overall satisfied with the modifications made and think that the study is now suitable for publication.

Before we can formally accept your manuscript, we would ask you to address the following issues.

Reviewer #1:

The authors have answered to all my criticism/comments in an adequate way. The manuscript has been extended and clarified. I recommend publication now.

Reviewer #2:

I thank the authors for their informative answers. They have satisfactorily answered all my comments. The only remaining concern is the absence of focal adhesion and stress fibers in Dictyostelium. I suppose acto-myosin cables can be seen, is that correct? In any case, can the authors speculate at how traction forces are generated and transmitted onto the substrate? It seems to be a critical point to understand their study.

Reviewer #3:

Following a first round of revision, the authors successfully clarified the minor points I raised, notably by explaining their choice and method for the use of very thin silicone gels of 1kPa. As suggested, they also agreed to display more examples of myosin labelled cells together with their force maps. They finally explained better their force data whenever required and made the appropriate corrections in the text/figure legends.
Regarding the other referees' points, it seems to me that the authors satisfactorily took into account their two main concerns. First, they spent some time clarifying their modelling part and the added value of such a model regarding what was already known in the litterature. To me, even if the model remains simple as it displays a rather limited number of parameters, it is in good agreement with the experimental conclusions of the work, supporting the hypothesis that both the force pattern and cell size rely on the spatiotemporal distribution of actin and myosin in cells and on the resulting balance between protrusive and contractile forces. Second, they also made a special effort to detail further the migration parameters in their study, which was one of the major concern of reviewer #2. Notably, they included some data where they compared cell speed as a function of area and total force for the different types of migrating cells.
To me, the work, although not entirely novel, offers a full characterization of both the cellular actomyosin spatiotemporal distribution and the force patterns generated by the same cells in the context of different types of migrations, providing a comprehensive understanding of the relationship between all these parameters. I am thus in favor of the publication of the manuscript.

We thank the Reviewers and you for your continued consideration of our study. We are excited to see that the Reviewers consider our study to be suitable for publication. Reviewer #2 has one remaining concern:

The only remaining concern is the absence of focal adhesion and stress fibers in Dictyostelium. I suppose acto-myosin cables can be seen, is that correct? In any case, can the authors speculate at how traction forces are generated and transmitted onto the substrate? It seems to be a critical point to understand their study.

We thank the Reviewer for this comment. Acto-myosin cables are not clearly visible in *Dictyostelium* cells. Furthermore, the precise nature of the adhesive forces required for the generation of traction forces is still not clear. Our previous studies have indicated that these cells can adhere to a wide variety of surfaces and that adhesions are transient that are marked with paxillin. These results suggest that the adhesion may be mediated by van der Waals and electrostatic interactions. To clarify this, we have added additional text and an additional reference into the text (line 489-495). Specifically, we have added:

Contrary to keratocytes, however, *Dictyostelium* cells do not exhibit stable focal adhesion complexes linked to stress fibers. Like neutrophils, they display transient adhesions marked with paxillin, although a specific integrin-extracellular matrix interaction has not been identified. *Dictyostelium* cells can adhere to a wide variety of surfaces (Bukharova et al, 2005; Loomis et al., 2012) and it is believed that non-specific van der Waals and electrostatic interactions play a role (Loomis et al., 2012; Tarantola et al., 2014). Therefore, it is likely that these forces, together with cytoskeletal flow, provide the required traction forces.

Thank you again for sending us your revised manuscript. We are now satisfied with the modifications made and I am pleased to inform you that your paper has been accepted for publication.

EMBO PRESS

YOU MUST COMPLETE ALL CELLS WITH A PINK BACKGROUND ➔

PLEASE NOTE THAT THIS CHECKLIST WILL BE PUBLISHED ALONGSIDE YOUR PAPER

Corresponding Author Name: Wouter-Jan Rappel
Journal Submitted to: Molecular Systems Biology
Manuscript Number: MSB-2021-10505

Reporting Checklist For Life Sciences Articles (Rev. June 2017)

This checklist is used to ensure good reporting standards and to improve the reproducibility of published results. These guidelines are consistent with the Principles and Guidelines for Reporting Preclinical Research issued by the NIH in 2014. Please follow the journal's authorship guidelines in preparing your manuscript.

A- Figures

1. Data

The data shown in figures should satisfy the following conditions:

- ➔ the data were obtained and processed according to the field's best practice and are presented to reflect the results of the experiments in an accurate and unbiased manner.
- ➔ figure panels include only data points, measurements or observations that can be compared to each other in a scientifically meaningful way.
- ➔ graphs include clearly labeled error bars for independent experiments and sample sizes. Unless justified, error bars should not be shown for technical replicates.
- ➔ if n< 5, the individual data points from each experiment should be plotted and any statistical test employed should be justified
- ➔ Source Data should be included to report the data underlying graphs. Please follow the guidelines set out in the author ship guidelines on Data Presentation.

2. Captions

Each figure caption should contain the following information, for each panel where they are relevant:

- ➔ a specification of the experimental system investigated (eg cell line, species name).
- ➔ the assay(s) and method(s) used to carry out the reported observations and measurements
- ➔ an explicit mention of the biological and chemical entity(ies) that are being measured.
- ➔ an explicit mention of the biological and chemical entity(ies) that are altered/varied/perturbed in a controlled manner.

- ➔ the exact sample size (n) for each experimental group/condition, given as a number, not a range;
- ➔ a description of the sample collection allowing the reader to understand whether the samples represent technical or biological replicates (including how many animals, litters, cultures, etc.).
- ➔ a statement of how many times the experiment shown was independently replicated in the laboratory.
- ➔ definitions of statistical methods and measures:
  - common tests, such as t-test (please specify whether paired vs. unpaired), simple χ2 tests, Wilcoxon and Mann-Whitney tests, can be unambiguously identified by name only, but more complex techniques should be described in the methods section;
  - are tests one-sided or two-sided?
  - are there adjustments for multiple comparisons?
  - exact statistical test results, e.g., P values = x but not P values < x;
  - definition of 'center values' as median or average;
  - definition of error bars as s.d. or s.e.m.

Any descriptions too long for the figure legend should be included in the methods section and/or with the source data.

In the pink boxes below, please ensure that the answers to the following questions are reported in the manuscript itself. Every question should be answered. If the question is not relevant to your research, please write NA (non applicable). We encourage you to include a specific subsection in the methods section for statistics, reagents, animal models and human subjects.

USEFUL LINKS FOR COMPLETING THIS FORM

http://www.antibodypedia.com
http://1degreebio.org
http://www.equator-network.org/reporting-guidelines/improving-bioscience-research-repor
http://grants.nih.gov/grants/olaw/olaw.htm
http://www.mrc.ac.uk/Ourresearch/Ethicsresearchguidance/Useofanimals/index.htm
http://ClinicalTrials.gov
http://www.consort-statement.org
http://www.consort-statement.org/checklists/view/32-consort/66-title
http://www.equator-network.org/reporting-guidelines/reporting-recommendations-for-tumo
http://datadryad.org
http://figshare.com
http://www.ncbi.nlm.nih.gov/gap
http://www.ebi.ac.uk/ega
http://biomodels.net/
http://biomodels.net/miriam/
http://jjj.biochem.sun.ac.za
https://osp.od.nih.gov/biosafety-biosecurity-and-emerging-biotechnology/
http://www.selectagents.gov/

B- Statistics and general methods

Please fill out these boxes ➔ (Do not worry if you cannot see all your text once you press return)

1.a. How was the sample size chosen to ensure adequate power to detect a pre-specified effect size?	N/A
1.b. For animal studies, include a statement about sample size estimate even if no statistical methods were used.	N/A
2. Describe inclusion/exclusion criteria if samples or animals were excluded from the analysis. Were the criteria pre-established?	N/A
3. Were any steps taken to minimize the effects of subjective bias when allocating animals/samples to treatment (e.g. randomization procedure)? If yes, please describe.	N/A
For animal studies, include a statement about randomization even if no randomization was used.	N/A
4.a. Were any steps taken to minimize the effects of subjective bias during group allocation or/and when assessing results (e.g. blinding of the investigator)? If yes please describe.	N/A
4.b. For animal studies, include a statement about blinding even if no blinding was done	N/A
5. For every figure, are statistical tests justified as appropriate?	Yes
Do the data meet the assumptions of the tests (e.g., normal distribution)? Describe any methods used to assess it.	Experiments were performed on at least two or three different days for each type of cells and for each type of motion. For data that was not normally distributed, data is reported as median (inter quartile 1/inter quartile 3) and the significance was evaluated with the Wilcoxon–Mann–Whitney test using the ranksum function in MATLAB. P-values higher that 0.05 are considered not significant, one star corresponds to 0.05>p>0.01, two stars to 0.01>p>0.001, three stars to 0.001>p>0.0001 and four stars to p<0.0001.
Is there an estimate of variation within each group of data?	N/A

Is the variance similar between the groups that are being statistically compared?	N/A

C- Reagents

6. To show that antibodies were profiled for use in the system under study (assay and species), provide a citation, catalog number and/or clone number, supplementary information or reference to an antibody validation profile. e.g., Antibodypedia (see link list at top right), 1DegreeBio (see link list at top right).	
7. Identify the source of cell lines and report if they were recently authenticated (e.g., by STR profiling) and tested for mycoplasma contamination.	

* for all hyperlinks, please see the table at the top right of the document

D- Animal Models

8. Report species, strain, gender, age of animals and genetic modification status where applicable. Please detail housing and husbandry conditions and the source of animals.	N/A
9. For experiments involving live vertebrates, include a statement of compliance with ethical regulations and identify the committee(s) approving the experiments.	N/A
10. We recommend consulting the ARRIVE guidelines (see link list at top right) (PLoS Biol. 8(6), e1000412, 2010) to ensure that other relevant aspects of animal studies are adequately reported. See author guidelines, under 'Reporting Guidelines'. See also: NIH (see link list at top right) and MRC (see link list at top right) recommendations. Please confirm compliance.	N/A

E- Human Subjects

11. Identify the committee(s) approving the study protocol.	N/A
12. Include a statement confirming that informed consent was obtained from all subjects and that the experiments conformed to the principles set out in the WMA Declaration of Helsinki and the Department of Health and Human Services Belmont Report.	N/A
13. For publication of patient photos, include a statement confirming that consent to publish was obtained.	N/A
14. Report any restrictions on the availability (and/or on the use) of human data or samples.	N/A
15. Report the clinical trial registration number (at ClinicalTrials.gov or equivalent), where applicable.	N/A
16. For phase II and III randomized controlled trials, please refer to the CONSORT flow diagram (see link list at top right) and submit the CONSORT checklist (see link list at top right) with your submission. See author guidelines, under 'Reporting Guidelines'. Please confirm you have submitted this list.	N/A
17. For tumor marker prognostic studies, we recommend that you follow the REMARK reporting guidelines (see link list at top right). See author guidelines, under 'Reporting Guidelines'. Please confirm you have followed these guidelines.	N/A

F- Data Accessibility

18: Provide a "Data Availability" section at the end of the Materials & Methods, listing the accession codes for data generated in this study and deposited in a public database (e.g. RNA-Seq data: Gene Expression Omnibus GSE39462, Proteomics data: PRIDE PXD000208 etc.) Please refer to our author guidelines for 'Data Deposition'.<br><br>Data deposition in a public repository is mandatory for:<br>a. Protein, DNA and RNA sequences<br>b. Macromolecular structures<br>c. Crystallographic data for small molecules<br>d. Functional genomics data<br>e. Proteomics and molecular interactions	Yes. The data of this study is available in the following database: dx.doi.org/10.6084/m9.figshare.16826740
19. Deposition is strongly recommended for any datasets that are central and integral to the study; please consider the journal's data policy. If no structured public repository exists for a given data type, we encourage the provision of datasets in the manuscript as a Supplementary Document (see author guidelines under 'Expanded View' or in unstructured repositories such as Dryad (see link list at top right) or Figshare (see link list at top right).	The datasets of the images in this study are available in the following database: dx.doi.org/10.6084/m9.figshare.16826740
20. Access to human clinical and genomic datasets should be provided with as few restrictions as possible while respecting ethical obligations to the patients and relevant medical and legal issues. If practically possible and compatible with the individual consent agreement used in the study, such data should be deposited in one of the major public access-controlled repositories such as dbGAP (see link list at top right) or EGA (see link list at top right).	N/A
21. Computational models that are central and integral to a study should be shared without restrictions and provided in a machine-readable form. The relevant accession numbers or links should be provided. When possible, standardized format (SBML, CellML) should be used instead of scripts (e.g. MATLAB). Authors are strongly encouraged to follow the MIRIAM guidelines (see link list at top right) and deposit their model in a public database such as Biomodels (see link list at top right) or JWS Online (see link list at top right). If computer source code is provided with the paper, it should be deposited in a public repository or included in supplementary information.	Computational code is deposited on https://github.com/Rappel-lab/Traction_force

G- Dual use research of concern

22. Could your study fall under dual use research restrictions? Please check biosecurity documents (see link list at top right) and list of select agents and toxins (APHIS/CDC) (see link list at top right). According to our biosecurity guidelines, provide a statement only if it could.	N/A
